# Automated risk scoring for venous thromboembolism using large language models with expert knowledge-augmented prompting: a multicenter validation study

**DOI:** 10.1038/s41746-026-02929-3

**Published:** 2026-07-09

**Authors:** Jing Ma, Dingyi Wang, Yaqian Zhang, Zhaofei Chen, Kaiyuan Zhen, Mengyao Li, Jingrui Li, Yunfan Shi, Xiaomeng Zhang, Rui Liang, Feiya Xu, Siqi Ma, Jintao Chen, Yanshuang Lyu, Yuanhua Yang, Zhe Cheng, Yutao Guo, Lei Xia, Wei Wang, Bing Liu, Cunbo Jia, Shuai Zhang, Qian Gao, Yunxia Zhang, Zhu Zhang, Wanmu Xie, Guohui Fan, Shengfeng Wang, Zhenguo Zhai

**Affiliations:** 1 Department of Respiratory and Critical Care Medicine, The Second Affiliated Hospital of Harbin Medical University, Harbin, China; 2 National Center for Respiratory Medicine; State Key Laboratory of Respiratory Health and Multimorbidity; National Clinical Research Center for Respiratory Diseases; Institute of Respiratory Medicine, Chinese Academy of Medical Sciences & Peking Union Medical College; Department of Clinical research and Data management, Center of Respiratory Medicine, China-Japan Friendship Hospital, Beijing, China; 3 Department of Respiratory and Critical Care Medicine, China-Japan Friendship Institute of Clinical Medicine, Beijing, China; 4 China-Japan Friendship Hospital, Chinese Academy of Medical Sciences & Peking Union Medical College, Beijing, China; 5 Respiratory Department, Center for Pulmonary Vascular Diseases, Fuwai Hospital, National Center for Cardiovascular Diseases, Chinese Academy of Medical Sciences and Peking Union Medical College, State Key Laboratory of Cardiovascular Disease, National Center for Cardiovascular Diseases, Beijing, China; 6 Peking University China-Japan Friendship School of Clinical Medicine, Beijing, China; 7 National Center for Respiratory Medicine; State Key Laboratory of Respiratory Health and Multimorbidity; National Clinical Research Center for Respiratory Diseases; Institute of Respiratory Medicine, Chinese Academy of Medical Sciences & Peking Union Medical College; Department of Pulmonary and Critical Care Medicine, Center of Respiratory Medicine, China-Japan Friendship Hospital, Beijing, China; 8 Capital Medical University, Beijing, China; 9 Peking University China-Japan Friendship School of Clinical Medicine; Department of Pulmonary and Critical Care Medicine; Center of Respiratory Medicine; National Center for Respiratory Medicine; Institute of Respiratory Medicine, Chinese Academy of Medical Sciences; National Clinical Research Center for Respiratory Diseases, China-Japan Friendship Hospital, Beijing, China; 10 Beijing University of Chinese Medicine, Beijing, China; 11 China-Japan Friendship Hospital (Institute of Clinical Medical Sciences), Chinese Academy of Medical Sciences & Peking Union Medical College, Beijing, China; 12 BioMap Co., Ltd, Beijing, China; 13 Zhejiang University, Hangzhou, China; 14 Department of Physiology, Institute of Basic Medical Sciences, Chinese Academy of Medical Sciences & Peking Union Medical College, Beijing, China; 15 Department of Pulmonary and Critical Care Medicine, Beijing Chao-Yang Hospital, Capital Medical University, Beijing, China; 16 Department of Pulmonary and Critical Care Medicine, The First Affiliated Hospital of Zhengzhou University, Zhengzhou, China; 17 Department of Pulmonary Vessel and Thrombotic Disease, Sixth Medical Center, Chinese PLA General Hospital, Beijing, China; 18 Medical Affairs Department of China-Japan Friendship Hospital, Beijing, China; 19 Department of Nursing, China-Japan Friendship Hospital, Beijing, China; 20 Key Laboratory of Epidemiology of Major Diseases (Peking University), Ministry of Education, Beijing, China; 21 China-Japan Friendship Hospital, Beijing, China; 22 Department of Epidemiology and Biostatistics, School of Public Health, Peking University, Beijing, China; 23 School of Population Medicine and Public Health, Chinese Academy of Medical Sciences & Peking Union Medical College; State Key Laboratory of Respiratory Health and Multimorbidity; Key Laboratory of Pathogen Infection Prevention and Control (Peking Union Medical College), Ministry of Education, Beijing, China

**Keywords:** Diseases, Health care, Medical research, Risk factors

## Abstract

Venous thromboembolism (VTE) is a common but preventable complication in hospitalized patients, yet standardized risk scoring using unstructured electronic health records (EHRs) remains challenging. We retrospectively analyzed anonymized EHRs from a nationwide multicenter cohort across 30 hospitals. Using stratified random sampling, 50 cases from 10 hospitals were used for development and 200 cases from the remaining 20 hospitals for testing. Expert knowledge-augmented prompts for the Padua and Caprini scores were developed through a Delphi process involving 19 specialists and compared with basic and complex prompts. Performance was evaluated using six open-source large language models (LLMs), with prevalence-adjusted bias-adjusted kappa (PABAK) and F1 score as primary metrics. Expert knowledge-augmented prompts showed the highest performance in both datasets. In the test dataset, mean item-level PABAK and F1 scores were 0.97 and 0.96 for Padua, and 0.97 and 0.93 for Caprini; most items showed PABAK and F1 scores above 0.90. Risk stratification was better for Padua (PABAK 0.92; F1 score 0.96) than for Caprini (PABAK 0.73; F1 score 0.64), with processing times of 6.07 s and 12.82 s per case, respectively. These findings indicated that LLMs with expert knowledge-augmented prompting could efficiently support automated Padua and Caprini scoring and risk stratification in thrombosis prevention workflows.

## Introduction

Venous thromboembolism (VTE) is associated with high mortality, disability, and recurrence rates^1^, and hospital-associated VTE is a common yet preventable complication among hospitalized patients^2^. Systematic risk assessment and appropriate prophylaxis can markedly reduce VTE incidence^3^. To guide clinical decision-making, the Padua and Caprini scores are widely used for VTE risk assessment in medical and surgical populations^4,5^. However, a recent study showed that VTE risk assessment was documented in only 42% of admitted patients, mainly because physicians were not aware that the assessment needed to be completed at admission, formal monitoring mechanisms were lacking, and the tool was not integrated into the routine admission workflow^6^. Moreover, the clinical information required to calculate these scores is often recorded in heterogeneous and unstructured formats within electronic health records (EHRs), highlighting the need for more efficient approaches to support timely and standardized score-based VTE risk stratification in real-world clinical practice.

A major challenge in automated VTE risk scoring is the lack of standardization in EHR documentation. Clinicians differ considerably in their recording styles and terminology, resulting in essential clinical information being stored as fragmented and heterogeneous free-text^7,8^. This makes manual scoring time-consuming and labor-intensive^9^. As a result, traditional natural language processing (NLP) techniques have been applied to extract structured information from EHR text, but previous studies have reported challenges in complex clinical environments. Traditional NLP approaches typically rely on rule construction or extensive manual annotation, have limited generalization across tasks, and struggle with terminology variation and context-dependent expressions^10,11^. The rapid development of generative large language models (LLMs) has provided new possibilities for clinical information extraction and automated risk scoring from unstructured EHRs. With strong capabilities in language understanding, contextual reasoning, and instruction following^12,13^, LLMs have demonstrated considerable potential for handling unstructured EHR data in previous studies^14,15^. To optimize their performance for specific tasks, fine-tuning and prompt engineering have been widely used in the medical field^16–18^. However, some studies have indicated that fine-tuning requires substantial computational and data resources while yielding relatively limited performance improvement^19,20^, which restricts its promotion in clinical settings. In contrast, well-designed prompt engineering holds immense potential in practical applications due to its flexibility across diverse scenarios and lower resource requirements compared with fine-tuning^18,21^. These advantages suggest that processing EHR data using LLMs with expert knowledge-augmented prompting may be reliable in VTE risk scoring in hospitalized patients.

Based on this, this study aimed to evaluate whether LLMs with expert knowledge-augmented prompting could accurately extract the clinical items required for Padua and Caprini scoring from real-world inpatient EHRs, thereby providing reliable clues and reference for downstream VTE risk stratification.

## Results

### Study workflow and cohort characteristics

**Fig. 1:**
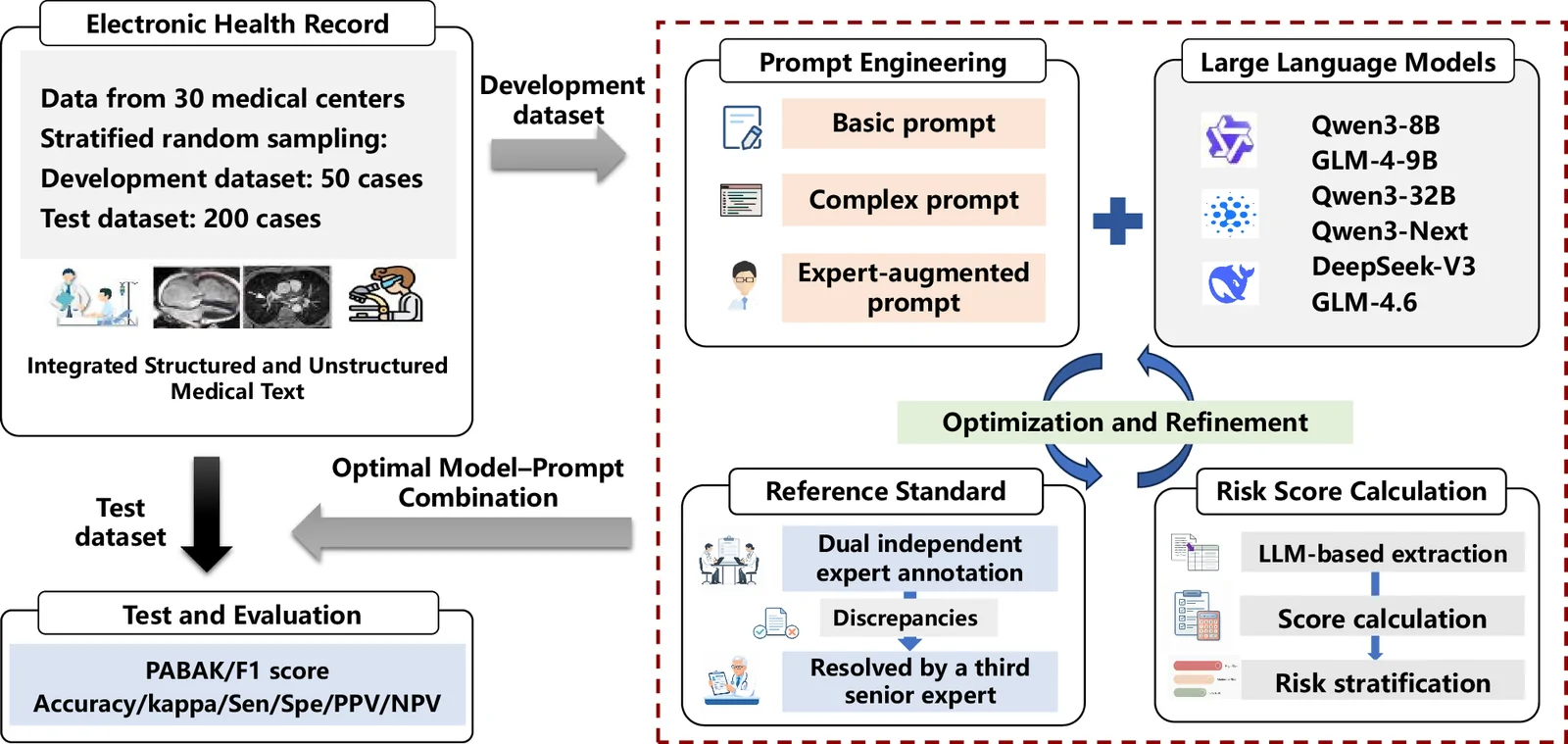
Workflow of LLM-based extraction and evaluation for Padua and Caprini risk scoring. Electronic health record data from 30 medical centers were stratified by center to construct a development dataset (50 cases) and a test dataset (200 cases). In the development dataset, different prompting strategies were combined with multiple large language models and underwent preliminary evaluation and iterative prompt optimization against a reference standard established by dual independent expert annotation with adjudication by a third senior expert. The optimal model-prompt combination was then applied to the test dataset and evaluated against the reference standard. Extracted items were further used for risk score calculation and risk stratification. Performance was primarily assessed using PABAK and F1 score, with accuracy, kappa, sensitivity, specificity, positive predictive value, and negative predictive value reported as supplementary metrics. PABAK prevalence-adjusted bias-adjusted kappa, Sen sensitivity, Spe specificity, PPV positive predictive value, NPV negative predictive value. Created by the authors using Microsoft PowerPoint.

This study adopted a two-stage design to evaluate whether LLMs can reliably achieve automated VTE risk scoring from unstructured clinical text (Fig. 1). In the development cohort (*n* = 50), three prompting strategies were applied across six LLMs and compared against an expert-annotated reference standard. Based on performance evaluation and error pattern analysis, the expert knowledge–augmented prompts were iteratively optimized, and the optimal model configuration was determined. In the test cohort (*n* = 200), the optimized prompting strategy was applied to the selected model, and the automatically generated Padua and Caprini scoring results were compared with the expert-annotated reference standard to evaluate its performance and generalizability in real-world clinical text.

Supplementary Table S1 summarizes characteristics of all patients enrolled from the 30 participating centers, stratified by center grouping (10 centers vs. 20 centers), and Supplementary Table S2 presents characteristics of development cohort (*n* = 50) and test cohort (*n* = 200) in this study. Comparable characteristics were observed between the random samples and totality.

Inter-rater reliability was high for all evaluable items. PABAK exceeded 0.85 for all items and was above 0.90 for most items (Supplementary Table S3).

### Performance in the development dataset

In the development dataset, performance metrics were used to compare prompting strategies and guide model selection. Expert knowledge–augmented prompting consistently showed higher performance than both basic and complex prompting strategies across all LLMs in both the Padua and Caprini scoring tasks (Supplementary Tables S4, 5). The full prompts we developed for all three strategies are provided in the Supplementary Information. Under this prompting strategy, performance varied across LLMs, with Qwen3-Next demonstrating the highest overall performance. For Padua, Qwen3-Next achieved a PABAK of 0.96 (95% CI: 0.94–0.98) and an F1 score of 0.93 (95% CI: 0.85–0.99). For Caprini, it also showed strong performance, with a PABAK of 0.94 (95% CI: 0.91–0.97) and an F1 score of 0.90 (95% CI: 0.84–0.95). Inference was completed within 20 s per case. Considering both performance and computational efficiency, Qwen3-Next was selected for subsequent testing. Detailed results for all models and prompting strategies are provided in Supplementary Tables S6–17.

### Performance in the test dataset

In the test dataset, expert knowledge–augmented prompting achieved the highest mean performance across all items for both Padua and Caprini scoring tasks (Table 1). For Padua, the mean PABAK was 0.97 (95% CI: 0.94–0.99), and the mean F1 score was 0.96 (95% CI: 0.94–0.99). For Caprini, the mean PABAK was 0.97 (95% CI: 0.95–0.98), and the mean F1 score was 0.93 (95% CI: 0.90–0.96), outperforming both basic and complex prompting strategies. The mean inference time was 6.07 s per case for Padua and 12.82 s per case for Caprini. The mean input token usage was 11,274.2 tokens per case for Padua and 20,187.0 tokens per case for Caprini.

**Table 1:** Macro-averaged performance of the Padua and Caprini scores across different prompting strategies in the test dataset

Risk scores	Prompt type	PABAK	F1-score	Accuracy	Kappa	Time per case (s)	Tokens per case
Padua	Basic prompt	0.83 (95% CI 0.62–0.97)	0.80 (95% CI 0.61–0.93)	0.91 (95% CI 0.79–0.98)	0.62 (95% CI 0.37–0.83)	5.21	6829.4
	Complex prompt	0.83 (95% CI 0.64–0.96)	0.78 (95% CI 0.60–0.92)	0.91 (95% CI 0.82–0.98)	0.61 (95% CI 0.37–0.82)	5.45	7519.0
	Expert prompt	0.97 (95% CI 0.94–0.99)	0.96 (95% CI 0.94–0.99)	0.99 (95% CI 0.98–0.99)	0.95 (95% CI 0.91–0.98)	6.07	11274.2
Caprini	Basic prompt	0.90 (95% CI 0.85–0.94)	0.75 (95% CI 0.67–0.82)	0.95 (95% CI 0.92–0.97)	0.66 (95% CI 0.56–0.76)	8.04	8133.0
	Complex prompt	0.93 (95% CI 0.88–0.96)	0.80 (95% CI 0.73–0.86)	0.96 (95% CI 0.94–0.98)	0.69 (95% CI 0.57–0.79)	8.39	10365.9
	Expert prompt	0.97 (95% CI 0.95–0.98)	0.93 (95% CI 0.90–0.96)	0.98 (95% CI 0.97–0.99)	0.88 (95% CI 0.81–0.94)	12.82	20187.0

Accuracy, kappa, PABAK, and F1-score were first calculated for each item and then summarized across items using macro-averaging.

*LLM* Large Language Model, *Kappa* Cohen’s Kappa coefficient, *PABAK* Prevalence-Adjusted Bias-Adjusted Kappa, *Time per case (s)* Average inference time per patient, *Tokens per case* average number of input tokens per patient.

### Item-level performance and error patterns analysis

For the Padua score, most items showed high agreement, with PABAK and F1 scores exceeding 0.90 for the majority of items. The item “reduced mobility” showed relatively lower agreement (PABAK 0.84; F1 score 0.90) (Table 2). For the Caprini score, high agreement was also observed across most items, with PABAK generally above 0.90. Lower performance was primarily observed in items dependent on temporal information or clinical context, including “minor surgery planned” (PABAK 0.87; F1 score 0.75), “major surgery >45 min” (PABAK 0.83; F1 score 0.83), “medical patient currently at bed rest” (PABAK 0.80; F1 score 0.62), and “patient confined to bed (>72 h)” (PABAK 0.77; F1 score 0.88) (Table 3). Results for the basic and complex prompts are provided in Supplementary Tables S18, 19. In addition, results from other LLMs are provided in Supplementary Tables S20–21. These findings further support the strong performance of Qwen3-Next.

**Table 2:** Item-level performance of expert knowledge-augmented prompting for Padua score extraction in the test dataset

Features	TP	FP	TN	FN	PABAK	F1	Accuracy	Kappa	Sensitivity	Specificity	PPV	NPV
Active cancer	44	1	155	0	0.99	0.99	0.99	0.99	1.00	0.99	0.98	1.00
Previous VTE	0	0	200	0	1.00	NA	1.00	NA	NA	1.00	NA	1.00
Elderly age (≥70 years)	63	3	134	0	0.97	0.98	0.98	0.97	1.00	0.98	0.95	1.00
Heart and/or respiratory failure	36	4	160	0	0.96	0.95	0.98	0.94	1.00	0.98	0.90	1.00
Acute infection and/or rheumatologic disorder	69	3	126	2	0.95	0.97	0.97	0.95	0.97	0.98	0.96	0.98
Obesity (BMI ≥ 30)	11	0	189	0	1.00	1.00	1.00	1.00	1.00	1.00	1.00	1.00
Ongoing hormonal treatment	2	0	198	0	1.00	1.00	1.00	1.00	1.00	1.00	1.00	1.00
Recent (≤1 month) trauma and/or surgery	10	0	188	2	0.98	0.91	0.99	0.90	0.83	1.00	1.00	0.99
Reduced mobility	75	3	109	13	0.84	0.90	0.92	0.84	0.85	0.97	0.96	0.89
Acute myocardial infarction or ischemic stroke	14	0	186	0	1.00	1.00	1.00	1.00	1.00	1.00	1.00	1.00
Already known thrombophilic condition	0	0	200	0	1.00	NA	1.00	NA	NA	1.00	NA	1.00

*TP* true positive; *FP*, false positive; *TN*, true negative; *FN*, false negative; *Kappa*, Cohen’s Kappa coefficient; *PABAK*, Prevalence-Adjusted Bias-Adjusted Kappa; *PPV*, positive predictive value; *NPV*, negative predictive value; *F1*, F1-score, *VTE* venous thromboembolism, *BMI* body mass index.

**Table 3:** Item-level performance of expert knowledge-augmented prompting for Caprini score extraction in the test dataset

Feature	TP	FP	TN	FN	PABAK	F1	Accuracy	Kappa	Sensitivity	specificity	PPV	NPV
Age (41–60 years)	36	1	157	6	0.93	0.91	0.96	0.89	0.86	0.99	0.97	0.96
Age (60–74 years)	69	11	117	3	0.86	0.91	0.93	0.85	0.96	0.91	0.86	0.97
Age (≥75 years)	42	6	151	1	0.93	0.92	0.96	0.90	0.98	0.96	0.88	0.99
Minor surgery planned	20	1	167	12	0.87	0.75	0.94	0.72	0.62	0.99	0.95	0.93
Obesity (BMI > 25)	44	0	156	0	1.00	1.00	1.00	1.00	1.00	1.00	1.00	1.00
Swollen legs (current)	25	0	175	0	1.00	1.00	1.00	1.00	1.00	1.00	1.00	1.00
Varicose veins	6	0	194	0	1.00	1.00	1.00	1.00	1.00	1.00	1.00	1.00
Pregnancy or postpartum (<1 month)	4	0	196	0	1.00	1.00	1.00	1.00	1.00	1.00	1.00	1.00
History of unexplained stillborn infant, recurrent spontaneous abortion (≥3), premature birth with toxemia or growth-restricted infant	0	0	200	0	1.00	NA	1.00	NA	NA	1.00	NA	1.00
Oral contraceptives or hormone replacement therapy	2	0	198	0	1.00	1.00	1.00	1.00	1.00	1.00	1.00	1.00
Sepsis (<1 month)	8	0	192	0	1.00	1.00	1.00	1.00	1.00	1.00	1.00	1.00
Serious lung disease incl.pneumonia (<1 month)	63	3	134	0	0.97	0.98	0.98	0.97	1.00	0.98	0.95	1.00
Abnormal pulmonary function	30	1	166	3	0.96	0.94	0.98	0.93	0.91	0.99	0.97	0.98
Acute myocardial infarction	3	0	197	0	1.00	1.00	1.00	1.00	1.00	1.00	1.00	1.00
Congestive heart failure (<1 month)	21	0	179	0	1.00	1.00	1.00	1.00	1.00	1.00	1.00	1.00
History of inflammatory bowel disease	0	0	200	0	1.00	NA	1.00	NA	NA	1.00	NA	1.00
Medical patient currently at bed rest	16	0	164	20	0.80	0.62	0.90	0.57	0.44	1.00	1.00	0.89
Arthroscopic surgery	2	0	198	0	1.00	1.00	1.00	1.00	1.00	1.00	1.00	1.00
Major surgery (>45 min)	43	4	140	13	0.83	0.83	0.92	0.78	0.77	0.97	0.91	0.92
Laparoscopic surgery (>45 min)	12	0	185	3	0.97	0.89	0.98	0.88	0.80	1.00	1.00	0.98
Malignancy (present or previous)	51	7	142	0	0.93	0.94	0.96	0.91	1.00	0.95	0.88	1.00
Patient confined to bed (>72 h)	85	20	92	3	0.77	0.88	0.88	0.77	0.97	0.82	0.81	0.97
Immobilizing plaster cast (<1 month)	4	0	195	1	0.99	0.89	0.99	0.89	0.80	1.00	1.00	0.99
central venous access	26	1	171	2	0.97	0.95	0.98	0.94	0.93	0.99	0.96	0.99
History of DVT/PE	0	0	200	0	1.00	NA	1.00	NA	NA	1.00	NA	1.00
Family history of thrombosis	0	0	200	0	1.00	NA	1.00	NA	NA	1.00	NA	1.00
Positive Factor V Leiden	0	0	200	0	1.00	NA	1.00	NA	NA	1.00	NA	1.00
Positive Prothrombin 20210 A	0	0	200	0	1.00	NA	1.00	NA	NA	1.00	NA	1.00
Positive lupus anticoagulant	0	1	199	0	0.99	NA	0.99	0.00	NA	0.99	0.00	1.00
Elevated anticardiolipin antibodies	1	1	198	0	0.99	0.67	0.99	0.66	1.00	0.99	0.50	1.00
Elevated serum homocysteine	5	0	195	0	1.00	1.00	1.00	1.00	1.00	1.00	1.00	1.00
Heparin-induced thrombocytopenia (HIT)	0	0	200	0	1.00	NA	1.00	NA	NA	1.00	NA	1.00
Other congenital or acquired thrombophilia	1	0	199	0	1.00	1.00	1.00	1.00	1.00	1.00	1.00	1.00
Stroke (<1 month)	19	1	180	0	0.99	0.97	0.99	0.97	1.00	0.99	0.95	1.00
Elective major lower extremity arthroplasty	4	0	196	0	1.00	1.00	1.00	1.00	1.00	1.00	1.00	1.00
Hip, pelvis or leg fracture (<1 month)	14	0	186	0	1.00	1.00	1.00	1.00	1.00	1.00	1.00	1.00
Acute spinal cord injury (paralysis) (<1 month)	0	0	200	0	1.00	NA	1.00	NA	NA	1.00	NA	1.00
Multiple trauma (<1 month)	7	3	190	0	0.97	0.82	0.98	0.82	1.00	0.98	0.70	1.00

*TP* true positive, *FP* false positive, *TN* true negative, *FN* false negative, *Kappa* Cohen’s Kappa coefficient, *PABAK* Prevalence-Adjusted Bias-Adjusted Kappa, *PPV* positive predictive value, *NPV* negative predictive value, *F1* F1-score, *DVT* deep vein thrombosis, *PE* pulmonary embolism, *BMI* body mass index.

We reviewed the CoT reasoning in a subset of misclassified cases and found that errors were not randomly distributed but mainly occurred under several specific conditions (Supplementary Table S22).

First, when explicit documentation of relevant items was lacking, the model relied on indirect information for inference. In this process, because the prompts could not fully cover all possible clinical scenarios, the model was prone to making incorrect inferences. For example, in the item “Patient confined to bed (>72 h)”, the original records did not explicitly document the patient’s activity status, yet the model inferred prolonged bed rest based on postoperative information such as “elevation of the affected limb” and “limb dressing”, resulting in reasoning mistake.

Second, some items require explicit diagnostic or specific laboratory evidence, and this requirement has been clearly specified during prompt construction. However, when this key evidence was missing and much relevant but confusing information existed in EHRs, reasoning errors occurred. For example, the LLM classified the “malignancy” item as true based on orders for tumor-related examinations or tests and some positive clues, despite the absence of explicit documentation confirming or excluding malignancy; similarly, the LLM classified the “positive lupus anticoagulant” item as positive based on related syndromes, even in the absence of explicit documentation.

Third, some errors were related to insufficient understanding of medical terminology. For example, procedures such as tracheostomy were classified as “major surgery (>45 min)”, and supportive respiratory measures such as “nasal oxygen therapy” and “routine respiratory care” were misinterpreted as evidence of respiratory failure.

### Risk stratification performance and generalizability

**Fig. 2:**
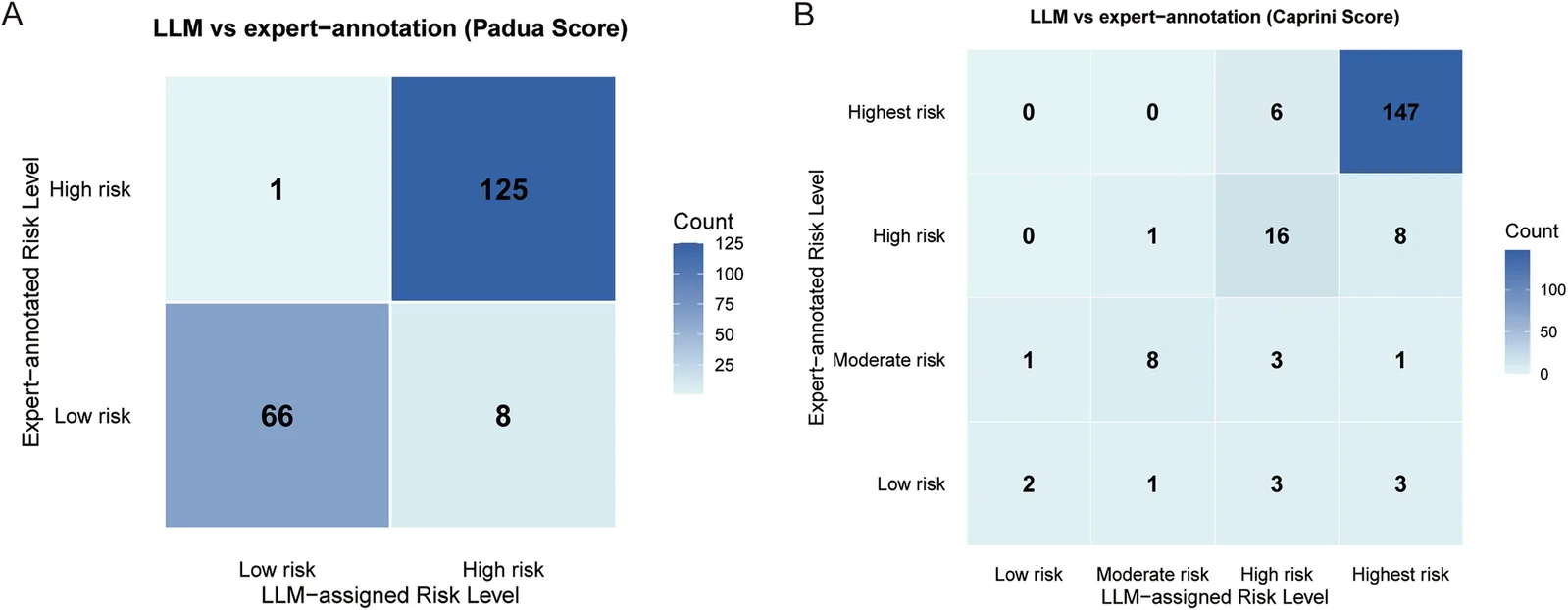
Confusion matrices of LLM vs. the expert-annotated reference standard for VTE risk stratification. Confusion matrices comparing the LLM and the expert-annotated reference standard for Padua and Caprini risk stratification: **A** Padua; **B** Caprini. Created by the authors using R (version 4.3.2).

Table 4 shows the performance of risk stratification after aggregating all item-level factors at the patient level. For the Padua score, the LLM showed high agreement with the expert-annotated reference standard across all risk stratification. The low-risk group showed 0.99 sensitivity and 0.89 specificity, while the high-risk group achieved 0.89 sensitivity and 0.99 specificity. The overall PABAK reached 0.92, and the F1 score was 0.96. As shown in Fig. 2A, only a small number of cases were misclassified, including 8 low-risk cases that were labeled as high-risk and 1 high-risk case that was labeled as low-risk. In contrast, the Caprini score showed considerable performance variation across different risk stratifications: its sensitivity was merely 0.22 in the low-risk group, while reaching 0.96 in the highest-risk group; the specificity remained generally high across all strata (except 0.75 in the highest-risk group). The model demonstrated an overall PABAK of 0.73, with an F1 score of 0.64. As shown in Fig. 2B, the model had an ability to identify high-risk and highest-risk patients, and a small number of moderate-low risk cases were overestimated as higher risk levels.

**Table 4:** Performance comparison with expert knowledge-augmented prompting versus the expert-annotated reference standard for Padua and Caprini scores in the test dataset

Risk score	Risk Level	Sensitivity	Specificity	PPV	NPV	F1	PABAK	Accuracy	Kappa
Padua	Low risk	0.99	0.89	0.94	0.99	0.97			
	High risk	0.89	0.99	0.99	0.94	0.94			
	Overall*					0.96	0.92	0.96	0.90
Caprini	Low risk	0.22	0.99	0.67	0.96	0.33			
	Moderate risk	0.62	0.99	0.80	0.97	0.70			
	High risk	0.64	0.93	0.57	0.95	0.60			
	Highest risk	0.96	0.75	0.93	0.85	0.94			
	Overall*					0.64	0.73	0.86	0.60

*Overall indicates the overall performance across all risk levels for each risk score. Kappa Cohen’s Kappa coefficient, *PABAK* Prevalence-Adjusted Bias-Adjusted Kappa, *PPV* positive predictive value, *NPV* negative predictive value, *F1* F1-score.

To further assess the consistency of model performance under expert knowledge-augmented prompting, we analyzed the mean item-level performance of the Padua and Caprini scores and the variability of these metrics across centers in both the development and test datasets (Supplementary Tables S23–26). Overall, performance across centers was excellent, and variability was low for most metrics, including PABAK and F1 score (SD < 0.1; CV < 0.1) (Supplementary Tables S23–26).

## Discussion

This study shows that LLMs with expert knowledge-augmented prompting can automatically extract VTE risk scores from multicenter real-world EHRs, supporting Padua and Caprini scoring with high agreement with expert annotations. By comparing six LLMs of different parameter scales, Qwen3-Next achieved the best balance of agreement and computational efficiency and was therefore selected for subsequent testing. In the test dataset, Qwen3-Next approached high agreement for the Padua score and maintained moderate agreement for the more complex Caprini score. The inference time for both scoring tasks remained within 20 s per case, indicating efficient computation. This study provides evidence that LLMs, without fine-tuning, can extract VTE risk scoring items directly from heterogeneous EHR text using expert-augmented prompting, thereby supporting automated Padua and Caprini risk stratification. This approach demonstrates favorable accuracy and computational efficiency, supporting its scalability to other disease-specific risk scoring and its potential translation into real-world clinical practice.

In Caprini stratification, the LLM showed high sensitivity for the highest-risk group (0.96) but low sensitivity for the low-risk group (0.22), reflecting a tendency toward risk overestimation, which reduced the likelihood of missing high-risk patients. This phenomenon was mainly caused by several factors. First, the absence of explicit documentation had a major impact on LLM performance. For many items, the EHR did not contain direct statements sufficient for definite classification, so the LLM had to rely on indirect information and contextual clues. This increased the likelihood of incorrect inferences. Second, the Caprini score includes nearly forty individual items, and misclassification of even single item, particularly those with higher weights, could alter the final risk category (low risk: 0–1, moderate risk: 2, high risk: 3–4, and highest risk: ≥5). Third, many items were expressed in highly variable and non-standardized ways in clinical documentation. Fourth, missing data further increased the difficulty of classification. When key laboratory results or examination findings were not fully available in the EHR text, the LLM had less evidence to support reliable classification and was more likely to make incorrect inferences. Overall, the LLM performed more stably on items with sufficient and clearly documented information, whereas errors were more likely to occur in items characterized by incomplete information, strong contextual dependence, or strict diagnostic criteria. Therefore, given the potential for clinical alarm fatigue or interference with prophylaxis workflows resulting from risk overestimation, this approach may be more appropriately positioned as a supportive tool for high-risk patient screening and clinical prompting, rather than as a stand-alone tool for confidently ruling out low-risk patients.

Automated Padua and Caprini scoring have been hindered by heavy reliance on manual review, inconsistent documentation practices, and variation in clinicians’ familiarity with scoring tools. Traditional methods have mostly been based on manual annotation and rule matching^22,23^, which may require additional adaptation and validation across institutions. Deep learning models have shown some performance in single-center Padua risk assessment, but their dependence on multiple medical knowledge bases restricts broader applicability^24^. Another study has used knowledge retrieval and LLM fine-tuning to extract risk factors, achieving accuracy of 79.56% and 88.32% for Caprini and Padua, respectively^25^, yet these approaches still require substantial labeled data and computational resources. Our approach relies on prompt optimization without model fine-tuning or external knowledge bases. It shows good performance across multicenter real-world EHRs and may provide a resource-friendly, scalable pathway for standardized VTE risk scoring.

Our study is consistent with the emerging shift toward “less fine-tuning and more prompt engineering”^26^. Prior studies have also shown that well-designed prompts can achieve performance comparable to or better than that of model fine-tuning in certain medical tasks^27^. With well-constructed prompts and expert knowledge, the model can extract clinical information without additional computational cost, supporting its potential integration into routine clinical workflows. As no prompt can fully cover the full range of clinical scenarios, expressions, and contextual variations encountered in real-world EHRs, further improvement is likely to depend not only on better prompt design, but also on continued advances in the underlying model itself. In addition, selecting an appropriate model is important for balancing performance and computational efficiency. Medium-parameter models with mixture-of-experts architectures activate only a subset of parameters during inference, offering strong reasoning capability while substantially reducing computational demands compared with very large models^28^. When combined with expert knowledge-augmented prompt engineering, such models have the potential for practical deployment in real-world hospital environments.

This study has several limitations. First, due to hardware constraints and data privacy considerations, we were not able to deploy closed-source LLMs such as GPT, and therefore could not assess whether they would perform better on the same task. Second, although the prompts were refined through multiple rounds of optimization, some items still had room for further improvement according to LLM performance. Third, missing data and non-standardized clinical documentation may lead to misclassification of certain items by influencing the information extraction performance of the LLM. Fourth, we did not directly compare the LLM approach with rule-based information extraction systems or conventional NLP methods; therefore, further work is needed to determine the incremental value and advantages of the LLM-based framework compared with conventional approaches. Fifth, the development dataset was relatively small and was used for both prompt refinement and model selection. This may have introduced selection bias in the final prompt strategy, although additional validation was performed using an independent test dataset. Finally, both the model and the data in this study were all based on Chinese language, and their applicability in English or other languages required context-specific adaptation. Despite these limitations, this study provides an important foundation for large-scale structured extraction of complex clinical records and offers useful experience for improving the standardization of clinical documentation.

In conclusion, general LLMs guided by expert knowledge-augmented prompting supported automated Padua and Caprini scoring and subsequent VTE risk stratification from multicenter EHRs with high efficiency. Our study indicated the potential to improve routine clinical workflows by introducing an approach to standardized automated VTE risk scoring and reducing manual workload.

## Methods

### Data source and preprocessing

This study was a post-hoc analysis of EHR data from the Chinese Prevention Strategy for Venous Thromboembolism (CHIPS-VTE), a prospective multicenter quality-improvement cohort conducted across 123 tertiary hospitals in China. Patients were consecutively screened for eligibility between April 1, 2024, and March 31, 2025. The source cohort included more than 60,000 adult inpatients at risk of VTE, with an expected hospital stay of ≥72 h, receiving medical or surgical care from multiple clinical departments. Exclusion criteria were admission solely for diagnostic procedures or hemodialysis, VTE present at admission, and hospitalization primarily for chronic non-acute conditions. This study was approved by the Ethics Committee of China-Japan Friendship Hospital, which served as the lead site (approval number: 2024-KY-033). Written informed consent was obtained from all participants or their legal representatives before enrollment.

In our study, 30 hospitals from 17 provinces, comprising more than 16,000 cases, were selected from CHIPS-VTE based on the availability of complete EHR data. All EHR data were de-identified locally at each participating hospital before analysis.

EHR preprocessing included the standardization of variable names, aligning date formats, and harmonizing file encodings. Records from different sources were merged by the patient’s unique identifier using key–value matching, so that information for the same patient was combined into a single text. Template-like or duplicated content was removed without altering any clinical information, ensuring that the medical narrative remained complete and coherent.

### Prompt engineering

In this study, the Padua and Caprini VTE risk scores were selected as the targets for information extraction. Item definitions and criteria were compiled from the original literature and relevant validation studies and were reviewed by 19 senior clinicians in respiratory and pulmonary vascular medicine across China to ensure alignment with clinical practice and common expressions in Chinese EHRs. A Delphi approach was adopted to establish expert consensus. All experts participated in the evaluation of all prompt versions across three rounds of review and discussion, with revisions made after each round. If disagreement persisted after the third round, the final decision was made by an independent senior expert. Based on these inputs, the core prompting framework of expert knowledge-augmented prompts was developed, including the task specification, evaluation rules, typical EHR expressions for each item and the requirements for structured outputs (reasoning traces and JSON format), and then iterated according to the actual performance and error pattern analysis in development dataset.

To demonstrate the advantage of these expert knowledge-augmented prompts and achieve an optimal design, another two types of prompts were also constructed:

1. Basic prompts: only provide the task objective and output format;
2. Complex prompts: add medical definitions and manually constructed input–output examples.

In the development dataset, the three prompting strategies were applied across six LLMs for comparative evaluation. The best-performing model–prompt combination was then applied to an independent test dataset.

### LLM deployment and inference

To ensure data security, context length requirements, and the comparability of models with different parameter scales, we deployed six open-source LLMs in a local secure environment. The models covered small, medium, and large parameter ranges to evaluate performance differences in this task. The small parameter models included Qwen3-8B^29^ and GLM-4-9B^30^; the medium parameter models included Qwen3-32B^29^ and Qwen3-Next-80B-A3B-Instruct (Qwen3-Next)^28^; and large parameter models included DeepSeek-V3^31^ and GLM-4.6^32^.

All models were accessed through an OpenAI-compatible API on the local server in full-precision inference versions rather than quantized ones. *YaRN* was used to extend the context window in order to accommodate long EHR inputs when necessary. Inference used the same configuration: temperature was set to 0, other parameters remained at default settings, and chain-of-thought (CoT) generation was enabled so that each model produced intermediate reasoning before the final structured output. These reasoning traces were retained for error analysis.

### Expert-annotated reference standard

An expert-annotated reference standard was established for this study. Two attending physicians independently reviewed each preprocessed EHR and assigned Padua and Caprini item-level annotations and corresponding scores; and any discrepancies were resolved by a third senior physician. All expert evaluations were based on the same preprocessed and anonymized EHR text provided to the LLMs, without access to any additional clinical information or source records. Reviewers were blinded to all LLM outputs throughout the annotation process.

### Statistical analysis

The 30 participating centers were randomly assigned to a development set (10 centers) and a test set (20 centers). A stratified random sampling strategy was then used, with center as the stratification factor. Within each center, cases were randomly sampled. The development dataset included 50 cases (5 cases per center) and was used for model selection, prompt refinement, and preliminary performance evaluation; the test dataset included 200 cases (10 cases per center) and was reserved for independent performance evaluation.

To determine an appropriate size for the independent test dataset, we estimated the minimum required sample size for stable evaluation of sensitivity and specificity using preliminary results from the development dataset. Both the normal approximation of the binomial distribution and the arcsine-transformed method were applied. At a significance level of α = 0.05 (Z₁₋α/₂ = 1.96), the allowable error for the expected sensitivity and specificity was set at 0.10. The formulas were as follows:

1 $$n=\frac{{Z}_{1-\alpha /2}^{2}p\left(1-p\right)}{{d}^{2}}$$

Here, *p* denotes the sensitivity or specificity estimated from the development dataset, *d* represents the allowable error (Z_₁₋α/₂_ = 1.96). For proportions close to 0 or 1, an arcsine transformation–based method was additionally applied to improve estimation stability. Based on these calculations, and taking into account the stratified sampling design across participating centers, the final test sample size was determined to be 200.

Each item in the Padua and Caprini scores was extracted from EHRs through the LLM inference in our study and returned in a structured format. Afterwards, the Padua and Caprini total scores, as well as the final risk stratification, were calculated using predefined scoring rules and then performing summation, rather than being directly generated by the LLM.

Using manual scoring as the expert-annotated reference standard, we evaluated the performance of LLMs in identifying Padua and Caprini score items and deriving the corresponding scores from EHRs. The primary performance metrics were prevalence-adjusted bias-adjusted kappa (PABAK) and F1 score. Cohen’s kappa was reported as a supplementary agreement metric. Accuracy, sensitivity, specificity, positive predictive value, and negative predictive value were also reported as supplementary metrics. Overall performance was summarized using macro-averaging across all items, so that each item contributed equally regardless of its prevalence. Because some items had very low positive prevalence, Cohen’s kappa can underestimate true agreement due to its dependence on prevalence and marginal imbalance. Therefore, PABAK was used as a primary index of agreement, calculated as^33^:

2 $${PABAK}=2* {Po}-1$$

Where Po denotes the proportion of observed agreement.

To quantify the uncertainty of overall performance estimates, 95% confidence intervals (CI) for overall accuracy, overall Cohen’s kappa, and overall PABAK were computed using a bootstrap resampling procedure (*n* = 2000), with the 2.5th and 97.5th percentiles of the resampling distribution used to define the CI. To describe variability in performance across centers, we calculated the mean, standard deviation (SD), and coefficient of variation (CV) for each hospital-level metric. The CV was defined as the SD divided by the mean across centers and was used as a descriptive measure of relative variability. All statistical analyses were conducted using R (version 4.3.2).

## Supplementary information

Supplementary Information

## Data Availability

The data underlying this article cannot be shared publicly due to privacy concerns but are available from the corresponding author on reasonable request with ethics committee approval.

## Code availability

The code used in this study can be obtained from the corresponding author upon reasonable request. The core components of the method (the prompt templates) are provided in the Supplementary Information.
