# Supplementary material for: Automated risk scoring for venous thromboembolism using large language models with expert knowledge-augmented prompting: a multicenter validation study
Source: NPJ Digit Med. 2026 Jul 9;9:840. doi: 10.1038/s41746-026-02929-3 (PMC13728113; doi:10.1038/s41746-026-02929-3)
Supplement: Supplementary file 1 [file 41746_2026_2929_MOESM1_ESM.pdf]

Supplementary Information

Automated Risk Scoring for Venous Thromboembolism Using Large Language Models with Expert Knowledge-Augmented Prompting: A Multicenter Validation Study

Supplementary Tables

Supplementary Table S1. Baseline Characteristics of the Overall Cohort Used for Sample Selection

Supplementary Table S2. Baseline Characteristics of the Study Population in the Development and Test Datasets

Supplementary Table S3. Item-Level Inter-Rater Agreement Metrics for the Padua and Caprini Scores

Supplementary Table S4. Macro-Averaged Item-Level Performance of the Padua Score Across Different LLMs and Prompting Strategies in the Development Dataset

Supplementary Table S5. Macro-Averaged Item-Level Performance of the Caprini Score Across Different LLMs and Prompting Strategies in the Development Dataset

Supplementary Table S6. Performance of Qwen3-8B with Different Prompting Strategies for Item-Level Padua Score Extraction in the Development Dataset

Supplementary Table S7. Performance of GLM-4-9B with Different Prompting Strategies for Item-Level Padua Score Extraction in the Development Dataset

Supplementary Table S8. Performance of Qwen3-32B with Different Prompting Strategies for Item-Level Padua Score Extraction in the Development Dataset

Supplementary Table S9. Performance of Qwen3-Next with Different Prompting Strategies for Item-Level Padua Score Extraction in the Development Dataset

Supplementary Table S10. Performance of Deepseek-V3 with Different Prompting Strategies for Item-Level Padua Score Extraction in the Development Dataset

Supplementary Table S11. Performance of GLM-4.6 with Different Prompting Strategies for Item-Level Padua Score Extraction in the Development Dataset

Supplementary Table S12. Performance of Qwen3-8B with Different Prompting Strategies for Item-Level Caprini Score Extraction in the Development Dataset

Supplementary Table S13. Performance of GLM-4-9B with Different Prompting Strategies for Item-Level Caprini Score Extraction in the Development Dataset

Supplementary Table S14. Performance of Qwen3-32B with Different Prompting Strategies for Item-Level Caprini Score Extraction in the Development Dataset

Supplementary Table S15. Performance of Qwen3-Next with Different Prompting Strategies for Item-Level Caprini Score Extraction in the Development Dataset

Supplementary Table S16. Performance of Deepseek-V3 with Different Prompting Strategies for Item-Level Caprini Score Extraction in the Development Dataset

Supplementary Table S17. Performance of GLM-4.6 with Different Prompting Strategies for Item-Level Caprini Score Extraction in the Development Dataset

Supplementary Table S18. Performance of Qwen3-Next with Basic and Complex Prompting Strategies for Item-Level Padua Score Extraction in the Test Dataset

Supplementary Table S19. Performance of Qwen3-Next with Basic and Complex Prompting Strategies for Item-Level Caprini Score Extraction in the Test Dataset

Supplementary Table S20. Macro-Averaged Item-Level Performance of the Padua Score Across Different LLMs and Prompting Strategies in the Test Dataset

Supplementary Table S21. Macro-Averaged Item-Level Performance of the Caprini Score Across Different LLMs and Prompting Strategies in the Test Dataset

Supplementary Table S22. Representative error cases in LLM-based risk factor extraction

Supplementary Table S23. Hospital-Level Performance and Inter-hospital Variability of the LLM for the Padua Score in the Development Dataset

Supplementary Table S24. Hospital-level performance and inter-hospital variability of the LLM for the Caprini Score in the Development Dataset

Supplementary Table S25. Hospital-level performance and inter-hospital variability of the LLM for the Padua score in the test Dataset

Supplementary Table S26. Hospital-level performance and inter-hospital variability of the LLM for the Caprini score in the test Dataset

Supplementary Information

Prompt templates for Padua score extraction

Prompt templates for Caprini score extraction

Supplementary Collaborating Centers

Supplementary Table S1. Baseline Characteristics of the Overall Cohort Used for Sample Selection

Characteristics	10 centers (N=5747)	20 centers (N=10910)
Age, years	61.0 (50.0–71.0)	59.0 (48.0–70.0)
BMI, kg/m^2	23.0 (20.9–25.2)	23.4 (21.3–25.8)
Male sex	2982 (51.9%)	5592 (51.3%)
Department type		
Medical	2780 (48.4%)	5127 (47.0%)
Surgical	2967 (51.6%)	5783 (53.0%)
Malignancy	1721 (30.7%)	2601 (24.1%)
Heart failure	638 (11.2%)	904 (8.3%)
Respiratory failure	327 (5.7%)	587 (5.4%)
Recent trauma	696 (12.4%)	1267 (11.9%)
Recent surgery	308 (5.5%)	507 (4.7%)

Note: Data are presented as median (interquartile range) for continuous variables and as number (percentage) for categorical variables.

Abbreviations: BMI, body mass index.

Supplementary Table S2. Baseline Characteristics of the Study Population in the Development and Test Datasets

Characteristics	Development set (N=50)	Test set (N=200)
Age, years	64.0 (52.0–70.8)	64.0 (52.0–74.0)
BMI, kg/m^2	23.6 (21.0–25.5)	23.2 (21.0–25.0)
Male sex	25 (50.0%)	98 (49.0%)
Department type		
Medical	29 (58.0%)	101 (50.5%)
Surgical	21 (42.0%)	99 (49.5%)
Malignancy	18 (36.0%)	51 (25.5%)
Heart failure	13 (26.0%)	21 (10.5%)
Respiratory failure	21 (42.0%)	13 (6.5%)
Recent trauma	11 (22.0%)	27 (13.7%)
Recent surgery	7 (14.0%)	12 (6.1%)

Note: Data are presented as median (interquartile range) for continuous variables and as number (percentage) for categorical variables.

Abbreviations: BMI, body mass index.

Supplementary Table S3. Item-Level Inter-Rater Agreement Metrics for the Padua and Caprini Scores

Items	sample size	PABAK	Agreement Rate	Kappa
**Padua score**				
Active cancer	250	0.99	1.00	0.99
Previous VTE	250	1.00	1.00	1.00
Elderly age (≥70 years)	250	0.96	0.98	0.95
Heart and/or respiratory failure	250	0.96	0.98	0.95
Acute infection and/or rheumatologic disorder	250	0.97	0.98	0.97
Obesity (BMI ≥30)	250	0.99	1.00	0.96
Ongoing hormonal treatment	250	1.00	1.00	1.00
Recent (≤1 month) trauma and/or surgery	250	0.98	0.99	0.93
Reduced mobility	250	0.88	0.94	0.88
Acute myocardial infarction or ischemic stroke	250	0.98	0.99	0.92
Already known thrombophilic condition	250	1.00	1.00	NA
**Caprini score**				
Age (41-60 years)	250	0.98	0.99	0.96
Age (60-74 years)	250	0.91	0.96	0.91
Age (≥75 years)	250	0.96	0.98	0.94
Minor surgery planned	250	0.88	0.94	0.80
Obesity (BMI>25)	250	1.00	1.00	1.00
Swollen legs (current)	250	0.99	1.00	0.99
Varicose veins	250	1.00	1.00	1.00
Pregnancy or postpartum (<1 month)	250	1.00	1.00	1.00
History of unexplained stillborn infant, recurrent spontaneous abortion (≥3), premature birth with toxemia or growth-restricted infant	250	1.00	1.00	NA
Oral contraceptives or hormone replacement therapy	250	0.99	1.00	0.86
Sepsis (<1 month)	250	0.93	0.96	0.81

Items	sample size	PABAK	Agreement Rate	Kappa
Serious lung disease incl.pneumonia (<1 month)	250	0.93	0.96	0.92
Abnormal pulmonary function	250	0.87	0.94	0.83
Acute myocardial infarction	250	0.99	1.00	0.89
Congestive heart failure (<1 month)	250	0.94	0.97	0.88
History of inflammatory bowel disease	250	1.00	1.00	NA
Medical patient currently at bed rest	250	0.94	0.97	0.84
Arthroscopic surgery	250	1.00	1.00	1.00
Major surgery (>45 minutes)	250	0.86	0.93	0.84
Laparoscopic surgery (>45 minutes)	250	0.95	0.98	0.85
Malignancy (present or previous)	250	0.98	0.99	0.97
Patient confined to bed (>72 hours)	250	0.88	0.94	0.88
Immobilizing plaster cast (<1 month)	250	1.00	1.00	1.00
central venous access	250	0.95	0.98	0.94
History of DVT/PE	250	1.00	1.00	NA
Family history of thrombosis	250	1.00	1.00	NA
Positive Factor V Leiden	250	1.00	1.00	NA
Positive Prothrombin 20210A	250	1.00	1.00	NA
Positive lupus anticoagulant	250	1.00	1.00	NA
Elevated anticardiolipin antibodies	250	1.00	1.00	1.00
Elevated serum homocysteine	250	1.00	1.00	1.00
Heparin-induced thrombocytopenia (HIT)	250	1.00	1.00	NA
Other congenital or acquired thrombophilia	250	1.00	1.00	1.00
Stroke (<1 month)	250	0.94	0.97	0.88
Elective major lower extremity arthroplasty	250	1.00	1.00	1.00
Hip, pelvis or leg fracture (<1 month)	250	0.99	1.00	0.98
Acute spinal cord injury (paralysis) (<1 month)	250	0.98	0.99	NA

Items	sample size	PABAK	Agreement Rate	Kappa
Multiple trauma (<1 month)	250	0.99	1.00	0.97

Notes: Agreement rate was defined as the proportion of items with concordant ratings between the two raters. Cohen's kappa was calculated to assess inter-rater reliability beyond chance agreement. Analyses were performed on all 250 cases, including the development dataset (N=50) and test dataset (N=200).

Abbreviations: Kappa, Cohen's kappa coefficient; PABAK, prevalence-adjusted bias-adjusted kappa.

Supplementary Table S4. Macro-Averaged Item-Level Performance of the Padua Score Across Different LLMs and Prompting Strategies in the Development Dataset

Prompt type	LLMs	PABAK	F1-score	Accuracy	Kappa	Time per Case (s)	Tokens per Case
**Basic prompt**	Qwen3-8B	0.65 (95%CI 0.36–0.88)	0.65 (95%CI 0.43–0.85)	0.82 (95%CI 0.67–0.94)	0.39 (95%CI 0.16–0.65)	21.15	20823.9
	GLM-4-9B	0.59 (95%CI 0.21–0.89)	0.59 (95%CI 0.35–0.81)	0.79 (95%CI 0.60–0.95)	0.40 (95%CI 0.16–0.66)	7.11	18046.7
	Qwen3-32B*	0.72 (95%CI 0.46–0.91)	0.76 (95%CI 0.56–0.93)	0.86 (95%CI 0.72–0.96)	0.50 (95%CI 0.24–0.74)	21.47	21660.3
	Qwen3-Next*	0.80 (95%CI 0.60–0.94)	0.76 (95%CI 0.55–0.96)	0.90 (95%CI 0.80–0.97)	0.57 (95%CI 0.34–0.78)	8.00	21203.1
	Deepseek-V3	0.65 (95%CI 0.37–0.88)	0.68 (95%CI 0.47–0.87)	0.83 (95%CI 0.69–0.94)	0.47 (95%CI 0.24–0.71)	12.68	17418.0
	GLM-4.6	0.73 (95%CI 0.51–0.91)	0.71 (95%CI 0.50–0.90)	0.86 (95%CI 0.76–0.96)	0.52 (95%CI 0.28–0.77)	21.50	18676.8
**Complex prompt**	Qwen3-8B	0.68 (95%CI 0.47–0.88)	0.72 (95%CI 0.53–0.89)	0.84 (95%CI 0.74–0.94)	0.42 (95%CI 0.17–0.68)	22.72	21486.2
	GLM-4-9B	0.56 (95%CI 0.17–0.87)	0.58 (95%CI 0.35–0.81)	0.78 (95%CI 0.61–0.93)	0.38 (95%CI 0.14–0.65)	8.18	18715.7
	Qwen3-32B*	0.67 (95%CI 0.42–0.88)	0.72 (95%CI 0.49–0.91)	0.83 (95%CI 0.70–0.94)	0.44 (95%CI 0.19–0.71)	23.54	22619.4
	Qwen3-Next*	0.80 (95%CI 0.61–0.93)	0.75 (95%CI 0.51–0.94)	0.90 (95%CI 0.81–0.97)	0.57 (95%CI 0.34–0.80)	8.00	21884.8
	Deepseek-V3	0.72 (95%CI 0.52–0.88)	0.69 (95%CI 0.48–0.89)	0.86 (95%CI 0.76–0.94)	0.46 (95%CI 0.23–0.71)	12.69	17963.4
	GLM-4.6	0.72 (95%CI 0.49–0.92)	0.71 (95%CI 0.49–0.90)	0.86 (95%CI 0.74–0.96)	0.51 (95%CI 0.27–0.75)	22.61	18687.7
**Expert prompt**	Qwen3-8B	0.74 (95%CI 0.55–0.92)	0.72 (95%CI 0.51–0.91)	0.87 (95%CI 0.77–0.95)	0.43 (95%CI 0.19–0.68)	25.25	25093.8
	GLM-4-9B	0.53 (95%CI 0.10–0.89)	0.55 (95%CI 0.32–0.79)	0.77 (95%CI 0.56–0.94)	0.38 (95%CI 0.15–0.64)	7.05	21874.2
	Qwen3-32B*	0.86 (95%CI 0.72–0.96)	0.88 (95%CI 0.80–0.96)	0.93 (95%CI 0.85–0.98)	0.58 (95%CI 0.33–0.81)	24.96	25938.1
	Qwen3-Next*	0.96 (95%CI 0.94–0.98)	0.93 (95%CI 0.85–0.99)	0.98 (95%CI 0.97–0.99)	0.79 (95%CI 0.58–0.94)	9.25	25605.7
	Deepseek-V3	0.81 (95%CI 0.68–0.94)	0.83 (95%CI 0.66–0.95)	0.91 (95%CI 0.84–0.97)	0.60 (95%CI 0.36–0.82)	14.31	21169.7
	GLM-4.6	0.90 (95%CI 0.82–0.97)	0.92 (95%CI 0.87–0.97)	0.95 (95%CI 0.91–0.98)	0.62 (95%CI 0.37–0.84)	24.80	22017.2

Note: * indicates thinking mode; Metrics including Accuracy, Kappa, PABAK, and F1-score were calculated at the item level for each individual Pauda variable and then summarized using macro-averaging across all items.

Abbreviations: LLM, Large Language Model; Kappa, Cohen's Kappa coefficient; CI, Confidence Interval; PABAK, Prevalence-Adjusted Bias-Adjusted Kappa; Time per case (s), Average inference time per patient; Tokens per case, average number of input tokens per patient.

Supplementary Table S5. Macro-Averaged Item-Level Performance of the Caprini Score Across Different LLMs and Prompting Strategies in the Development Dataset

Prompt type	LLMs	PABAK	F1-score	Accuracy	Kappa	Time per Case (s)	Tokens per Case
**Basic prompt**	Qwen3-8B	0.84 (95%CI: 0.74–0.92)	0.76 (95%CI: 0.66–0.84)	0.92 (95%CI: 0.87–0.96)	0.56 (95%CI: 0.42–0.69)	38.69	24301.0
	GLM-4-9B	0.74 (95%CI: 0.62–0.85)	0.57 (95%CI: 0.49–0.64)	0.87 (95%CI: 0.80–0.92)	0.36 (95%CI: 0.25–0.47)	10.78	19061.7
	Qwen3-32B*	0.84 (95%CI: 0.74–0.92)	0.77 (95%CI: 0.66–0.86)	0.92 (95%CI: 0.87–0.96)	0.54 (95%CI: 0.39–0.68)	61.80	22927.2
	Qwen3-Next*	0.86 (95%CI: 0.78–0.92)	0.78 (95%CI: 0.67–0.87)	0.93 (95%CI: 0.89–0.96)	0.57 (95%CI: 0.41–0.71)	11.99	22774.8
	Deepseek-V3	0.85 (95%CI: 0.77–0.91)	0.68 (95%CI: 0.56–0.79)	0.92 (95%CI: 0.88–0.96)	0.52 (95%CI: 0.40–0.66)	16.19	18234.3
	GLM-4.6	0.87 (95%CI: 0.81–0.92)	0.82 (95%CI: 0.72–0.90)	0.93 (95%CI: 0.90–0.96)	0.56 (95%CI: 0.40–0.70)	95.41	19806.4
**Complex prompt**	Qwen3-8B	0.85 (95%CI: 0.76–0.92)	0.76 (95%CI: 0.67–0.85)	0.92 (95%CI: 0.88–0.96)	0.55 (95%CI: 0.41–0.69)	36.79	26312.9
	GLM-4-9B	0.72 (95%CI: 0.59–0.84)	0.45 (95%CI: 0.33–0.58)	0.86 (95%CI: 0.79–0.92)	0.26 (95%CI: 0.15–0.39)	12.69	20313.7
	Qwen3-32B*	0.85 (95%CI: 0.76–0.93)	0.78 (95%CI: 0.69–0.86)	0.92 (95%CI: 0.88–0.96)	0.57 (95%CI: 0.43–0.70)	66.49	25152.6
	Qwen3-Next*	0.90 (95%CI: 0.85–0.93)	0.82 (95%CI: 0.73–0.89)	0.95 (95%CI: 0.93–0.97)	0.64 (95%CI: 0.50–0.75)	12.66	25065.9
	Deepseek-V3	0.86 (95%CI: 0.80–0.92)	0.81 (95%CI: 0.74–0.87)	0.93 (95%CI: 0.90–0.96)	0.55 (95%CI: 0.41–0.70)	18.27	20318.1
	GLM-4.6	0.88 (95%CI: 0.82–0.93)	0.88 (95%CI: 0.83–0.93)	0.94 (95%CI: 0.91–0.97)	0.58 (95%CI: 0.42–0.74)	103.44	22082.2
**Expert prompt**	Qwen3-8B	0.84 (95%CI: 0.73–0.92)	0.76 (95%CI: 0.68–0.84)	0.92 (95%CI: 0.87–0.96)	0.59 (95%CI: 0.45–0.72)	34.22	35172.7
	GLM-4-9B	0.72 (95%CI: 0.57–0.85)	0.51 (95%CI: 0.39–0.63)	0.86 (95%CI: 0.79–0.92)	0.30 (95%CI: 0.18–0.42)	9.69	29582.6
	Qwen3-32B*	0.84 (95%CI: 0.74–0.92)	0.74 (95%CI: 0.65–0.82)	0.92 (95%CI: 0.87–0.96)	0.56 (95%CI: 0.43–0.69)	60.58	34500.7
	Qwen3-Next*	0.94 (95%CI: 0.91–0.97)	0.90 (95%CI: 0.84–0.95)	0.97 (95%CI: 0.96–0.98)	0.76 (95%CI: 0.64–0.87)	15.77	34652.3
	Deepseek-V3	0.88 (95%CI: 0.82–0.93)	0.78 (95%CI: 0.68–0.86)	0.94 (95%CI: 0.91–0.97)	0.59 (95%CI: 0.44–0.72)	26.68	28629.1
	GLM-4.6	0.91 (95%CI: 0.86–0.95)	0.85 (95%CI: 0.76–0.92)	0.95 (95%CI: 0.93–0.98)	0.66 (95%CI: 0.51–0.79)	109.22	28280.2

Note: * indicates thinking mode; Metrics including Accuracy, Kappa, PABAK, and F1-score were calculated at the item level for each individual Caprini variable and then summarized using macro-averaging across all items.

Abbreviations: LLM, Large Language Model; Kappa, Cohen's Kappa coefficient; CI, Confidence Interval; PABAK, Prevalence-Adjusted Bias-Adjusted Kappa; Time per case (s), Average inference time per patient; Tokens per case, average number of input tokens per patient.

Supplementary Table S6. Performance of Qwen3-8B with Different Prompting Strategies for Item-Level Padua Score Extraction in the Development Dataset

prompt	Items	TP	FP	TN	FN	PABAK	F1	Accuracy	Kappa	Sensitivity	Specificity	PPV	NPV
Basic prompt	Active cancer	16	0	33	1	0.96	0.97	0.98	0.95	0.94	1.00	1.00	0.97
	Previous VTE	0	1	48	1	0.92	NA	0.96	-0.02	0.00	0.98	0.00	0.98
	Elderly age (≥70 years)	14	0	36	0	1.00	1.00	1.00	1.00	1.00	1.00	1.00	1.00
	Heart and/or respiratory failure	17	0	19	14	0.44	0.71	0.72	0.48	0.55	1.00	1.00	0.58
	Acute infection and/or rheumatologic disorder	28	0	4	18	0.28	0.76	0.64	0.20	0.61	1.00	1.00	0.18
	Obesity (BMI ≥30)	3	1	46	0	0.96	0.86	0.98	0.85	1.00	0.98	0.75	1.00
	Ongoing hormonal treatment	0	0	48	2	0.92	NA	0.96	0.00	0.00	1.00	NA	0.96
	Recent (≤1 month) trauma and/or surgery	1	14	33	2	0.36	0.11	0.68	0.01	0.33	0.70	0.07	0.94
	Reduced mobility	10	0	2	38	-0.52	0.34	0.24	0.02	0.21	1.00	1.00	0.05
	Acute myocardial infarction or ischemic stroke	2	1	43	4	0.80	0.44	0.90	0.40	0.33	0.98	0.67	0.91
	Already known thrombophilic condition	0	0	50	0	1.00	NA	1.00	NA	NA	1.00	NA	1.00
Complex prompt	Active cancer	17	0	33	0	1.00	1.00	1.00	1.00	1.00	1.00	1.00	1.00
	Previous VTE	0	2	47	1	0.88	NA	0.94	-0.03	0.00	0.96	0.00	0.98
	Elderly age (≥70 years)	14	0	36	0	1.00	1.00	1.00	1.00	1.00	1.00	1.00	1.00
	Heart and/or respiratory failure	16	0	19	15	0.40	0.68	0.70	0.45	0.52	1.00	1.00	0.56
	Acute infection and/or rheumatologic disorder	26	0	4	20	0.20	0.72	0.60	0.17	0.57	1.00	1.00	0.17
	Obesity (BMI ≥30)	3	0	47	0	1.00	1.00	1.00	1.00	1.00	1.00	1.00	1.00
	Ongoing hormonal treatment	0	0	48	2	0.92	NA	0.96	0.00	0.00	1.00	NA	0.96
	Recent (≤1 month) trauma and/or surgery	3	19	28	0	0.24	0.24	0.62	0.15	1.00	0.60	0.14	1.00
	Reduced mobility	26	0	2	22	0.12	0.70	0.56	0.09	0.54	1.00	1.00	0.08
	Acute myocardial infarction or ischemic stroke	2	2	42	4	0.76	0.40	0.88	0.34	0.33	0.95	0.50	0.91
	Already known thrombophilic condition	0	0	50	0	1.00	NA	1.00	NA	NA	1.00	NA	1.00
Expert prompt	Active cancer	17	0	33	0	1.00	1.00	1.00	1.00	1.00	1.00	1.00	1.00
	Previous VTE	0	0	49	1	0.96	NA	0.98	0.00	0.00	1.00	NA	0.98

prompt	Items	TP	FP	TN	FN	PABAK	F1	Accuracy	Kappa	Sensitivity	Specificity	PPV	NPV
	Elderly age (≥70 years)	14	1	35	0	0.96	0.97	0.98	0.95	1.00	0.97	0.93	1.00
	Heart and/or respiratory failure	15	0	19	16	0.36	0.65	0.68	0.42	0.48	1.00	1.00	0.54
	Acute infection and/or rheumatologic disorder	22	0	4	24	0.04	0.65	0.52	0.13	0.48	1.00	1.00	0.14
	Obesity (BMI ≥30)	3	0	47	0	1.00	1.00	1.00	1.00	1.00	1.00	1.00	1.00
	Ongoing hormonal treatment	0	0	48	2	0.92	NA	0.96	0.00	0.00	1.00	NA	0.96
	Recent (≤1 month) trauma and/or surgery	1	12	35	2	0.44	0.12	0.72	0.03	0.33	0.74	0.08	0.95
	Reduced mobility	39	0	2	9	0.64	0.90	0.82	0.26	0.81	1.00	1.00	0.18
	Acute myocardial infarction or ischemic stroke	2	0	44	4	0.84	0.50	0.92	0.47	0.33	1.00	1.00	0.92
	Already known thrombophilic condition	0	0	50	0	1.00	NA	1.00	NA	NA	1.00	NA	1.00

Abbreviations: TP, true positive; FP, false positive; TN, true negative; FN, false negative; Kappa, Cohen's Kappa coefficient; PABAK, Prevalence-Adjusted Bias-Adjusted Kappa; PPV, positive predictive value; NPV, negative predictive value; F1, F1-score; VTE, venous thromboembolism; BMI, body mass index.

Supplementary Table S7. Performance of GLM-4-9B with Different Prompting Strategies for Item-Level Padua Score Extraction in the Development Dataset

prompt	Items	TP	FP	TN	FN	PABAK	F1	Accuracy	Kappa	Sensitivity	Specificity	PPV	NPV
**Basic prompt**	Active cancer	17	0	33	0	1.00	1.00	1.00	1.00	1.00	1.00	1.00	1.00
	Previous VTE	0	0	49	1	0.96	NA	0.98	0.00	0.00	1.00	NA	0.98
	Elderly age (≥70 years)	11	0	36	3	0.88	0.88	0.94	0.84	0.79	1.00	1.00	0.92
	Heart and/or respiratory failure	18	0	19	13	0.48	0.73	0.74	0.51	0.58	1.00	1.00	0.59
	Acute infection and/or rheumatologic disorder	11	0	4	35	-0.40	0.39	0.30	0.05	0.24	1.00	1.00	0.10
	Obesity (BMI ≥30)	3	0	47	0	1.00	1.00	1.00	1.00	1.00	1.00	1.00	1.00
	Ongoing hormonal treatment	0	0	48	2	0.92	NA	0.96	0.00	0.00	1.00	NA	0.96
	Recent (≤1 month) trauma and/or surgery	1	7	40	2	0.64	0.18	0.82	0.10	0.33	0.85	0.12	0.95
	Reduced mobility	2	0	2	46	-0.84	0.08	0.08	0.00	0.04	1.00	1.00	0.04
	Acute myocardial infarction or ischemic stroke	2	0	44	4	0.84	0.50	0.92	0.47	0.33	1.00	1.00	0.92
	Already known thrombophilic condition	0	0	50	0	1.00	NA	1.00	NA	NA	1.00	NA	1.00
**Complex prompt**	Active cancer	17	0	33	0	1.00	1.00	1.00	1.00	1.00	1.00	1.00	1.00
	Previous VTE	0	0	49	1	0.96	NA	0.98	0.00	0.00	1.00	NA	0.98
	Elderly age (≥70 years)	13	1	35	1	0.92	0.93	0.96	0.90	0.93	0.97	0.93	0.97
	Heart and/or respiratory failure	15	0	19	16	0.36	0.65	0.68	0.42	0.48	1.00	1.00	0.54
	Acute infection and/or rheumatologic disorder	8	0	4	38	-0.52	0.30	0.24	0.03	0.17	1.00	1.00	0.10
	Obesity (BMI ≥30)	3	0	47	0	1.00	1.00	1.00	1.00	1.00	1.00	1.00	1.00
	Ongoing hormonal treatment	0	0	48	2	0.92	NA	0.96	0.00	0.00	1.00	NA	0.96
	Recent (≤1 month) trauma and/or surgery	2	13	34	1	0.44	0.22	0.72	0.14	0.67	0.72	0.13	0.97
	Reduced mobility	6	0	2	42	-0.68	0.22	0.16	0.01	0.12	1.00	1.00	0.05
	Acute myocardial infarction or ischemic stroke	2	3	41	4	0.72	0.36	0.86	0.29	0.33	0.93	0.40	0.91
	Already known thrombophilic condition	0	0	50	0	1.00	NA	1.00	NA	NA	1.00	NA	1.00
**Expert prompt**	Active cancer	15	0	33	2	0.92	0.94	0.96	0.91	0.88	1.00	1.00	0.94
	Previous VTE	0	0	49	1	0.96	NA	0.98	0.00	0.00	1.00	NA	0.98

prompt	Items	TP	FP	TN	FN	PABAK	F1	Accuracy	Kappa	Sensitivity	Specificity	PPV	NPV
	Elderly age (≥70 years)	13	0	36	1	0.96	0.96	0.98	0.95	0.93	1.00	1.00	0.97
	Heart and/or respiratory failure	10	0	19	21	0.16	0.49	0.58	0.27	0.32	1.00	1.00	0.47
	Acute infection and/or rheumatologic disorder	4	0	4	42	-0.68	0.16	0.16	0.02	0.09	1.00	1.00	0.09
	Obesity (BMI ≥30)	3	0	47	0	1.00	1.00	1.00	1.00	1.00	1.00	1.00	1.00
	Ongoing hormonal treatment	0	0	48	2	0.92	NA	0.96	0.00	0.00	1.00	NA	0.96
	Recent (≤1 month) trauma and/or surgery	2	7	40	1	0.68	0.33	0.84	0.27	0.67	0.85	0.22	0.98
	Reduced mobility	2	0	2	46	-0.84	0.08	0.08	0.00	0.04	1.00	1.00	0.04
	Acute myocardial infarction or ischemic stroke	2	1	43	4	0.80	0.44	0.90	0.40	0.33	0.98	0.67	0.91
	Already known thrombophilic condition	0	0	50	0	1.00	NA	1.00	NA	NA	1.00	NA	1.00

Abbreviations: TP, true positive; FP, false positive; TN, true negative; FN, false negative; Kappa, Cohen's Kappa coefficient; PABAK, Prevalence-Adjusted Bias-Adjusted Kappa; PPV, positive predictive value; NPV, negative predictive value; F1, F1-score; VTE, venous thromboembolism; BMI, body mass index.

Supplementary Table S8. Performance of Qwen3-32B with Different Prompting Strategies for Item-Level Padua Score Extraction in the Development Dataset

prompt	Items	TP	FP	TN	FN	PABAK	F1	Accuracy	Kappa	Sensitivity	Specificity	PPV	NPV
Basic prompt	Active cancer	17	0	33	0	1.00	1.00	1.00	1.00	1.00	1.00	1.00	1.00
	Previous VTE	0	4	45	1	0.80	NA	0.90	-0.03	0.00	0.92	0.00	0.98
	Elderly age (≥70 years)	14	0	36	0	1.00	1.00	1.00	1.00	1.00	1.00	1.00	1.00
	Heart and/or respiratory failure	20	0	19	11	0.56	0.78	0.78	0.58	0.65	1.00	1.00	0.63
	Acute infection and/or rheumatologic disorder	41	0	4	5	0.80	0.94	0.90	0.57	0.89	1.00	1.00	0.44
	Obesity (BMI ≥30)	3	0	47	0	1.00	1.00	1.00	1.00	1.00	1.00	1.00	1.00
	Ongoing hormonal treatment	0	0	48	2	0.92	NA	0.96	0.00	0.00	1.00	NA	0.96
	Recent (≤1 month) trauma and/or surgery	3	12	35	0	0.52	0.33	0.76	0.26	1.00	0.74	0.20	1.00
	Reduced mobility	11	0	2	37	-0.48	0.37	0.26	0.02	0.23	1.00	1.00	0.05
	Acute myocardial infarction or ischemic stroke	4	2	42	2	0.84	0.67	0.92	0.62	0.67	0.95	0.67	0.95
	Already known thrombophilic condition	0	0	50	0	1.00	NA	1.00	NA	NA	1.00	NA	1.00
Complex prompt	Active cancer	16	0	33	1	0.96	0.97	0.98	0.95	0.94	1.00	1.00	0.97
	Previous VTE	0	7	42	1	0.68	NA	0.84	-0.04	0.00	0.86	0.00	0.98
	Elderly age (≥70 years)	14	0	36	0	1.00	1.00	1.00	1.00	1.00	1.00	1.00	1.00
	Heart and/or respiratory failure	22	0	19	9	0.64	0.83	0.82	0.65	0.71	1.00	1.00	0.68
	Acute infection and/or rheumatologic disorder	40	0	4	6	0.76	0.93	0.88	0.52	0.87	1.00	1.00	0.40
	Obesity (BMI ≥30)	3	0	47	0	1.00	1.00	1.00	1.00	1.00	1.00	1.00	1.00
	Ongoing hormonal treatment	0	0	48	2	0.92	NA	0.96	0.00	0.00	1.00	NA	0.96
	Recent (≤1 month) trauma and/or surgery	2	21	26	1	0.12	0.15	0.56	0.05	0.67	0.55	0.09	0.96
	Reduced mobility	15	0	2	33	-0.32	0.48	0.34	0.04	0.31	1.00	1.00	0.06
	Acute myocardial infarction or ischemic stroke	3	7	37	3	0.60	0.37	0.80	0.26	0.50	0.84	0.30	0.92
	Already known thrombophilic condition	0	0	50	0	1.00	NA	1.00	NA	NA	1.00	NA	1.00
Expert prompt	Active cancer	16	0	33	1	0.96	0.97	0.98	0.95	0.94	1.00	1.00	0.97
	Previous VTE	0	0	49	1	0.96	NA	0.98	0.00	0.00	1.00	NA	0.98

prompt	Items	TP	FP	TN	FN	PABAK	F1	Accuracy	Kappa	Sensitivity	Specificity	PPV	NPV
	Elderly age (≥70 years)	14	0	36	0	1.00	1.00	1.00	1.00	1.00	1.00	1.00	1.00
	Heart and/or respiratory failure	28	2	17	3	0.80	0.92	0.90	0.79	0.90	0.89	0.93	0.85
	Acute infection and/or rheumatologic disorder	40	0	4	6	0.76	0.93	0.88	0.52	0.87	1.00	1.00	0.40
	Obesity (BMI ≥30)	3	0	47	0	1.00	1.00	1.00	1.00	1.00	1.00	1.00	1.00
	Ongoing hormonal treatment	0	0	48	2	0.92	NA	0.96	0.00	0.00	1.00	NA	0.96
	Recent (≤1 month) trauma and/or surgery	3	3	44	0	0.88	0.67	0.94	0.64	1.00	0.94	0.50	1.00
	Reduced mobility	29	0	2	19	0.24	0.75	0.62	0.11	0.60	1.00	1.00	0.10
	Acute myocardial infarction or ischemic stroke	5	1	43	1	0.92	0.83	0.96	0.81	0.83	0.98	0.83	0.98
	Already known thrombophilic condition	0	0	50	0	1.00	NA	1.00	NA	NA	1.00	NA	1.00

Abbreviations: TP, true positive; FP, false positive; TN, true negative; FN, false negative; Kappa, Cohen's Kappa coefficient; PABAK, Prevalence-Adjusted Bias-Adjusted Kappa; PPV, positive predictive value; NPV, negative predictive value; F1, F1-score; VTE, venous thromboembolism; BMI, body mass index.

Supplementary Table S9. Performance of Qwen3-Next with Different Prompting Strategies for Item-Level Padua Score Extraction in the Development Dataset

prompt	Items	TP	FP	TN	FN	PABAK	F1	Accuracy	Kappa	Sensitivity	Specificity	PPV	NPV
**Basic prompt**	Active cancer	17	0	33	0	1.00	1.00	1.00	1.00	1.00	1.00	1.00	1.00
	Previous VTE	1	6	43	0	0.76	0.25	0.88	0.22	1.00	0.88	0.14	1.00
	Elderly age (≥70 years)	14	0	36	0	1.00	1.00	1.00	1.00	1.00	1.00	1.00	1.00
	Heart and/or respiratory failure	25	0	19	6	0.76	0.89	0.88	0.76	0.81	1.00	1.00	0.76
	Acute infection and/or rheumatologic disorder	43	0	4	3	0.88	0.97	0.94	0.70	0.93	1.00	1.00	0.57
	Obesity (BMI ≥30)	3	0	47	0	1.00	1.00	1.00	1.00	1.00	1.00	1.00	1.00
	Ongoing hormonal treatment	0	0	48	2	0.92	NA	0.96	0.00	0.00	1.00	NA	0.96
	Recent (≤1 month) trauma and/or surgery	3	27	20	0	-0.08	0.18	0.46	0.08	1.00	0.43	0.10	1.00
	Reduced mobility	42	0	2	6	0.76	0.93	0.88	0.36	0.88	1.00	1.00	0.25
	Acute myocardial infarction or ischemic stroke	5	5	39	1	0.76	0.62	0.88	0.56	0.83	0.89	0.50	0.97
	Already known thrombophilic condition	0	0	50	0	1.00	NA	1.00	NA	NA	1.00	NA	1.00
**Complex prompt**	Active cancer	17	0	33	0	1.00	1.00	1.00	1.00	1.00	1.00	1.00	1.00
	Previous VTE	1	7	42	0	0.72	0.22	0.86	0.19	1.00	0.86	0.12	1.00
	Elderly age (≥70 years)	14	0	36	0	1.00	1.00	1.00	1.00	1.00	1.00	1.00	1.00
	Heart and/or respiratory failure	24	0	19	7	0.72	0.87	0.86	0.72	0.77	1.00	1.00	0.73
	Acute infection and/or rheumatologic disorder	45	0	4	1	0.96	0.99	0.98	0.88	0.98	1.00	1.00	0.80
	Obesity (BMI ≥30)	3	0	47	0	1.00	1.00	1.00	1.00	1.00	1.00	1.00	1.00
	Ongoing hormonal treatment	0	0	48	2	0.92	NA	0.96	0.00	0.00	1.00	NA	0.96
	Recent (≤1 month) trauma and/or surgery	3	24	23	0	0.04	0.20	0.52	0.10	1.00	0.49	0.11	1.00
	Reduced mobility	40	0	2	8	0.68	0.91	0.84	0.29	0.83	1.00	1.00	0.20
	Acute myocardial infarction or ischemic stroke	4	4	40	2	0.76	0.57	0.88	0.50	0.67	0.91	0.50	0.95
	Already known thrombophilic condition	0	0	50	0	1.00	NA	1.00	NA	NA	1.00	NA	1.00
**Expert prompt**	Active cancer	17	0	33	0	1.00	1.00	1.00	1.00	1.00	1.00	1.00	1.00
	Previous VTE	1	0	49	0	1.00	1.00	1.00	1.00	1.00	1.00	1.00	1.00

prompt	Items	TP	FP	TN	FN	PABAK	F1	Accuracy	Kappa	Sensitivity	Specificity	PPV	NPV
	Elderly age (≥70 years)	14	0	36	0	1.00	1.00	1.00	1.00	1.00	1.00	1.00	1.00
	Heart and/or respiratory failure	29	0	19	2	0.92	0.97	0.96	0.92	0.94	1.00	1.00	0.90
	Acute infection and/or rheumatologic disorder	45	0	4	1	0.96	0.99	0.98	0.88	0.98	1.00	1.00	0.80
	Obesity (BMI ≥30)	3	0	47	0	1.00	1.00	1.00	1.00	1.00	1.00	1.00	1.00
	Ongoing hormonal treatment	0	0	48	2	0.92	NA	0.96	0.00	0.00	1.00	NA	0.96
	Recent (≤1 month) trauma and/or surgery	2	1	46	1	0.92	0.67	0.96	0.65	0.67	0.98	0.67	0.98
	Reduced mobility	46	0	2	2	0.92	0.98	0.96	0.65	0.96	1.00	1.00	0.50
	Acute myocardial infarction or ischemic stroke	4	0	44	2	0.92	0.80	0.96	0.78	0.67	1.00	1.00	0.96
	Already known thrombophilic condition	0	0	50	0	1.00	NA	1.00	NA	NA	1.00	NA	1.00

Abbreviations: TP, true positive; FP, false positive; TN, true negative; FN, false negative; Kappa, Cohen's Kappa coefficient; PABAK, Prevalence-Adjusted Bias-Adjusted Kappa; PPV, positive predictive value; NPV, negative predictive value; F1, F1-score; VTE, venous thromboembolism; BMI, body mass index.

Supplementary Table S10. Performance of Deepseek-V3 with Different Prompting Strategies for Item-Level Padua Score Extraction in the Development Dataset

prompt	Items	TP	FP	TN	FN	PABAK	F1	Accuracy	Kappa	Sensitivity	Specificity	PPV	NPV
Basic prompt	Active cancer	17	0	33	0	1.00	1.00	1.00	1.00	1.00	1.00	1.00	1.00
	Previous VTE	1	2	47	0	0.92	0.50	0.96	0.48	1.00	0.96	0.33	1.00
	Elderly age (≥70 years)	14	0	36	0	1.00	1.00	1.00	1.00	1.00	1.00	1.00	1.00
	Heart and/or respiratory failure	20	0	19	11	0.56	0.78	0.78	0.58	0.65	1.00	1.00	0.63
	Acute infection and/or rheumatologic disorder	31	0	4	15	0.40	0.81	0.70	0.25	0.67	1.00	1.00	0.21
	Obesity (BMI ≥30)	3	0	47	0	1.00	1.00	1.00	1.00	1.00	1.00	1.00	1.00
	Ongoing hormonal treatment	0	0	48	2	0.92	NA	0.96	0.00	0.00	1.00	NA	0.96
	Recent (≤1 month) trauma and/or surgery	2	21	26	1	0.12	0.15	0.56	0.05	0.67	0.55	0.09	0.96
	Reduced mobility	13	0	2	35	-0.40	0.43	0.30	0.03	0.27	1.00	1.00	0.05
	Acute myocardial infarction or ischemic stroke	3	5	39	3	0.68	0.43	0.84	0.34	0.50	0.89	0.38	0.93
	Already known thrombophilic condition	0	0	50	0	1.00	NA	1.00	NA	NA	1.00	NA	1.00
Complex prompt	Active cancer	17	0	33	0	1.00	1.00	1.00	1.00	1.00	1.00	1.00	1.00
	Previous VTE	1	7	42	0	0.72	0.22	0.86	0.19	1.00	0.86	0.12	1.00
	Elderly age (≥70 years)	14	0	36	0	1.00	1.00	1.00	1.00	1.00	1.00	1.00	1.00
	Heart and/or respiratory failure	22	0	19	9	0.64	0.83	0.82	0.65	0.71	1.00	1.00	0.68
	Acute infection and/or rheumatologic disorder	36	1	3	10	0.56	0.87	0.78	0.26	0.78	0.75	0.97	0.23
	Obesity (BMI ≥30)	3	0	47	0	1.00	1.00	1.00	1.00	1.00	1.00	1.00	1.00
	Ongoing hormonal treatment	0	0	48	2	0.92	NA	0.96	0.00	0.00	1.00	NA	0.96
	Recent (≤1 month) trauma and/or surgery	2	17	30	1	0.28	0.18	0.64	0.09	0.67	0.64	0.11	0.97
	Reduced mobility	25	0	2	23	0.08	0.68	0.54	0.08	0.52	1.00	1.00	0.08
	Acute myocardial infarction or ischemic stroke	3	5	39	3	0.68	0.43	0.84	0.34	0.50	0.89	0.38	0.93
	Already known thrombophilic condition	0	0	50	0	1.00	NA	1.00	NA	NA	1.00	NA	1.00
Expert prompt	Active cancer	16	0	33	1	0.96	0.97	0.98	0.95	0.94	1.00	1.00	0.97
	Previous VTE	1	0	49	0	1.00	1.00	1.00	1.00	1.00	1.00	1.00	1.00

prompt	Items	TP	FP	TN	FN	PABAK	F1	Accuracy	Kappa	Sensitivity	Specificity	PPV	NPV
	Elderly age (≥70 years)	14	0	36	0	1.00	1.00	1.00	1.00	1.00	1.00	1.00	1.00
	Heart and/or respiratory failure	22	0	19	9	0.64	0.83	0.82	0.65	0.71	1.00	1.00	0.68
	Acute infection and/or rheumatologic disorder	36	0	4	10	0.60	0.88	0.80	0.37	0.78	1.00	1.00	0.29
	Obesity (BMI ≥30)	3	0	47	0	1.00	1.00	1.00	1.00	1.00	1.00	1.00	1.00
	Ongoing hormonal treatment	0	0	48	2	0.92	NA	0.96	0.00	0.00	1.00	NA	0.96
	Recent (≤1 month) trauma and/or surgery	2	8	39	1	0.64	0.31	0.82	0.24	0.67	0.83	0.20	0.97
	Reduced mobility	31	0	2	17	0.32	0.78	0.66	0.13	0.65	1.00	1.00	0.11
	Acute myocardial infarction or ischemic stroke	3	0	44	3	0.88	0.67	0.94	0.64	0.50	1.00	1.00	0.94
	Already known thrombophilic condition	0	0	50	0	1.00	NA	1.00	NA	NA	1.00	NA	1.00

Abbreviations: TP, true positive; FP, false positive; TN, true negative; FN, false negative; Kappa, Cohen's Kappa coefficient; PABAK, Prevalence-Adjusted Bias-Adjusted Kappa; PPV, positive predictive value; NPV, negative predictive value; F1, F1-score; VTE, venous thromboembolism; BMI, body mass index.

Supplementary Table S11. Performance of GLM-4.6 with Different Prompting Strategies for Item-Level Padua Score Extraction in the Development Dataset

prompt	Items	TP	FP	TN	FN	Accuracy	Kappa	PABAK	Sensitivity	Specificity	PPV	NPV	F1
Basic prompt	Active cancer	17	0	33	0	1.00	1.00	1.00	1.00	1.00	1.00	1.00	1.00
	Previous VTE	1	5	44	0	0.90	0.26	0.80	1.00	0.90	0.17	1.00	0.29
	Elderly age (≥70 years)	14	0	36	0	1.00	1.00	1.00	1.00	1.00	1.00	1.00	1.00
	Heart and/or respiratory failure	20	0	19	11	0.78	0.58	0.56	0.65	1.00	1.00	0.63	0.78
	Acute infection and/or rheumatologic disorder	43	0	4	3	0.94	0.70	0.88	0.93	1.00	1.00	0.57	0.97
	Obesity (BMI ≥30)	3	0	47	0	1.00	1.00	1.00	1.00	1.00	1.00	1.00	1.00
	Ongoing hormonal treatment	0	0	48	2	0.96	0.00	0.92	0.00	1.00	NA	0.96	NA
	Recent (≤1 month) trauma and/or surgery	2	22	25	1	0.54	0.05	0.08	0.67	0.53	0.08	0.96	0.15
	Reduced mobility	23	0	2	25	0.50	0.07	0.00	0.48	1.00	1.00	0.07	0.65
	Acute myocardial infarction or ischemic stroke	4	4	40	2	0.88	0.50	0.76	0.67	0.91	0.50	0.95	0.57
	Already known thrombophilic condition	0	0	50	0	1.00	NA	1.00	NA	1.00	NA	1.00	NA
Complex prompt	Active cancer	17	0	33	0	1.00	1.00	1.00	1.00	1.00	1.00	1.00	1.00
	Previous VTE	1	5	44	0	0.90	0.26	0.80	1.00	0.90	0.17	1.00	0.29
	Elderly age (≥70 years)	14	0	36	0	1.00	1.00	1.00	1.00	1.00	1.00	1.00	1.00
	Heart and/or respiratory failure	19	0	19	12	0.76	0.55	0.52	0.61	1.00	1.00	0.61	0.76
	Acute infection and/or rheumatologic disorder	42	0	4	4	0.92	0.63	0.84	0.91	1.00	1.00	0.50	0.95
	Obesity (BMI ≥30)	3	0	47	0	1.00	1.00	1.00	1.00	1.00	1.00	1.00	1.00
	Ongoing hormonal treatment	0	0	48	2	0.96	0.00	0.92	0.00	1.00	NA	0.96	NA
	Recent (≤1 month) trauma and/or surgery	2	25	22	1	0.48	0.03	-0.04	0.67	0.47	0.07	0.96	0.13
	Reduced mobility	24	0	2	24	0.52	0.07	0.04	0.50	1.00	1.00	0.08	0.67
	Acute myocardial infarction or ischemic stroke	4	3	41	2	0.90	0.56	0.80	0.67	0.93	0.57	0.95	0.62
	Already known thrombophilic condition	0	0	50	0	1.00	NA	1.00	NA	1.00	NA	1.00	NA
Expert prompt	Active cancer	17	0	33	0	1.00	1.00	1.00	1.00	1.00	1.00	1.00	1.00
	Previous VTE	0	0	49	1	0.98	0.00	0.96	0.00	1.00	NA	0.98	NA

prompt	Items	TP	FP	TN	FN	Accuracy	Kappa	PABAK	Sensitivity	Specificity	PPV	NPV	F1
	Elderly age (≥70 years)	14	0	36	0	1.00	1.00	1.00	1.00	1.00	1.00	1.00	1.00
	Heart and/or respiratory failure	27	1	18	4	0.90	0.79	0.80	0.87	0.95	0.96	0.82	0.92
	Acute infection and/or rheumatologic disorder	40	0	4	6	0.88	0.52	0.76	0.87	1.00	1.00	0.40	0.93
	Obesity (BMI ≥30)	3	0	47	0	1.00	1.00	1.00	1.00	1.00	1.00	1.00	1.00
	Ongoing hormonal treatment	0	0	48	2	0.96	0.00	0.92	0.00	1.00	NA	0.96	NA
	Recent (≤1 month) trauma and/or surgery	3	1	46	0	0.98	0.85	0.96	1.00	0.98	0.75	1.00	0.86
	Reduced mobility	38	0	2	10	0.80	0.23	0.60	0.79	1.00	1.00	0.17	0.88
	Acute myocardial infarction or ischemic stroke	4	0	44	2	0.96	0.78	0.92	0.67	1.00	1.00	0.96	0.80
	Already known thrombophilic condition	0	0	50	0	1.00	NA	1.00	NA	1.00	NA	1.00	NA

Abbreviations: TP, true positive; FP, false positive; TN, true negative; FN, false negative; Kappa, Cohen's Kappa coefficient; PABAK, Prevalence-Adjusted Bias-Adjusted Kappa; PPV, positive predictive value; NPV, negative predictive value; F1, F1-score; VTE, venous thromboembolism; BMI, body mass index.

Supplementary Table S12. Performance of Qwen3-8B with Different Prompting Strategies for Item-Level Caprini Score Extraction in the Development Dataset

prompt	Items	TP	FP	TN	FN	PABAK	F1	Accuracy	Kappa	Sensitivity	Specificity	PPV	NPV
Basic prompt	Age (41-60 years)	11	1	38	0	0.96	0.96	0.98	0.94	1.00	0.97	0.92	1.00
	Age (60-74 years)	21	0	27	2	0.92	0.95	0.96	0.92	0.91	1.00	1.00	0.93
	Age (≥75 years)	6	1	43	0	0.96	0.92	0.98	0.91	1.00	0.98	0.86	1.00
	Minor surgery planned	4	3	36	7	0.60	0.44	0.80	0.33	0.36	0.92	0.57	0.84
	Obesity (BMI>25)	12	1	36	1	0.92	0.92	0.96	0.90	0.92	0.97	0.92	0.97
	Swollen legs (current)	8	1	33	8	0.64	0.64	0.82	0.53	0.50	0.97	0.89	0.80
	Varicose veins	0	3	47	0	0.88	NA	0.94	0.00	NA	0.94	0.00	1.00
	Pregnancy or postpartum (<1 month)	0	1	49	0	0.96	NA	0.98	0.00	NA	0.98	0.00	1.00
	History of unexplained stillborn infant, recurrent spontaneous abortion (≥3), premature birth with toxemia or growth-restricted infant	0	0	50	0	1.00	NA	1.00	NA	NA	1.00	NA	1.00
	Oral contraceptives or hormone replacement therapy	0	0	50	0	1.00	NA	1.00	NA	NA	1.00	NA	1.00
	Sepsis (<1 month)	7	7	29	7	0.44	0.50	0.72	0.31	0.50	0.81	0.50	0.81
	Serious lung disease incl.pneumonia (<1 month)	15	0	18	17	0.32	0.64	0.66	0.39	0.47	1.00	1.00	0.51
	Abnormal pulmonary function	2	0	23	25	0.00	0.14	0.50	0.07	0.07	1.00	1.00	0.48
	Acute myocardial infarction	1	0	48	1	0.96	0.67	0.98	0.66	0.50	1.00	1.00	0.98
	Congestive heart failure (<1 month)	12	0	37	1	0.96	0.96	0.98	0.95	0.92	1.00	1.00	0.97
	History of inflammatory bowel disease	0	0	50	0	1.00	NA	1.00	NA	NA	1.00	NA	1.00
	Medical patient currently at bed rest	0	0	50	0	1.00	NA	1.00	NA	NA	1.00	NA	1.00
	Arthroscopic surgery	0	0	50	0	1.00	NA	1.00	NA	NA	1.00	NA	1.00
	Major surgery (>45 minutes)	19	6	24	1	0.72	0.84	0.86	0.72	0.95	0.80	0.76	0.96

prompt	Items	TP	FP	TN	FN	PABAK	F1	Accuracy	Kappa	Sensitivity	Specificity	PPV	NPV
	Laparoscopic surgery (>45 minutes)	4	2	44	0	0.92	0.80	0.96	0.78	1.00	0.96	0.67	1.00
	Malignancy (present or previous)	13	0	32	5	0.80	0.84	0.90	0.77	0.72	1.00	1.00	0.86
	Patient confined to bed (>72 hours)	17	0	2	31	-0.24	0.52	0.38	0.04	0.35	1.00	1.00	0.06
	Immobilizing plaster cast (<1 month)	4	0	45	1	0.96	0.89	0.98	0.88	0.80	1.00	1.00	0.98
	central venous access	33	3	10	4	0.72	0.90	0.86	0.65	0.89	0.77	0.92	0.71
	History of DVT/PE	0	2	48	0	0.92	NA	0.96	0.00	NA	0.96	0.00	1.00
	Family history of thrombosis	0	0	50	0	1.00	NA	1.00	NA	NA	1.00	NA	1.00
	Positive Factor V Leiden	0	0	50	0	1.00	NA	1.00	NA	NA	1.00	NA	1.00
	Positive Prothrombin 20210A	0	0	50	0	1.00	NA	1.00	NA	NA	1.00	NA	1.00
	Positive lupus anticoagulant	0	0	50	0	1.00	NA	1.00	NA	NA	1.00	NA	1.00
	Elevated anticardiolipin antibodies	0	0	50	0	1.00	NA	1.00	NA	NA	1.00	NA	1.00
	Elevated serum homocysteine	4	0	46	0	1.00	1.00	1.00	1.00	1.00	1.00	1.00	1.00
	Heparin-induced thrombocytopenia (HIT)	0	0	50	0	1.00	NA	1.00	NA	NA	1.00	NA	1.00
	Other congenital or acquired thrombophilia	0	1	49	0	0.96	NA	0.98	0.00	NA	0.98	0.00	1.00
	Stroke (<1 month)	8	1	36	5	0.76	0.73	0.88	0.65	0.62	0.97	0.89	0.88
	Elective major lower extremity arthroplasty	0	0	50	0	1.00	NA	1.00	NA	NA	1.00	NA	1.00
	Hip, pelvis or leg fracture (<1 month)	8	1	39	2	0.88	0.84	0.94	0.81	0.80	0.97	0.89	0.95
	Acute spinal cord injury (paralysis) (<1 month)	0	0	50	0	1.00	NA	1.00	NA	NA	1.00	NA	1.00
	Multiple trauma (<1 month)	8	2	38	2	0.84	0.80	0.92	0.75	0.80	0.95	0.80	0.95
Complex prompt	Age (41-60 years)	11	2	37	0	0.92	0.92	0.96	0.89	1.00	0.95	0.85	1.00
	Age (60-74 years)	22	0	27	1	0.96	0.98	0.98	0.96	0.96	1.00	1.00	0.96
	Age (≥75 years)	6	0	44	0	1.00	1.00	1.00	1.00	1.00	1.00	1.00	1.00
	Minor surgery planned	1	1	38	10	0.56	0.15	0.78	0.09	0.09	0.97	0.50	0.79
	Obesity (BMI>25)	12	0	37	1	0.96	0.96	0.98	0.95	0.92	1.00	1.00	0.97
	Swollen legs (current)	6	2	32	10	0.52	0.50	0.76	0.36	0.38	0.94	0.75	0.76

prompt	Items	TP	FP	TN	FN	PABAK	F1	Accuracy	Kappa	Sensitivity	Specificity	PPV	NPV
	Varicose veins	0	1	49	0	0.96	NA	0.98	0.00	NA	0.98	0.00	1.00
	Pregnancy or postpartum (<1 month)	0	0	50	0	1.00	NA	1.00	NA	NA	1.00	NA	1.00
	History of unexplained stillborn infant, recurrent spontaneous abortion (≥3), premature birth with toxemia or growth-restricted infant	0	0	50	0	1.00	NA	1.00	NA	NA	1.00	NA	1.00
	Oral contraceptives or hormone replacement therapy	0	1	49	0	0.96	NA	0.98	0.00	NA	0.98	0.00	1.00
	Sepsis (<1 month)	10	3	33	4	0.72	0.74	0.86	0.65	0.71	0.92	0.77	0.89
	Serious lung disease incl.pneumonia (<1 month)	21	1	17	11	0.52	0.78	0.76	0.54	0.66	0.94	0.95	0.61
	Abnormal pulmonary function	9	0	23	18	0.28	0.50	0.64	0.32	0.33	1.00	1.00	0.56
	Acute myocardial infarction	1	1	47	1	0.92	0.50	0.96	0.48	0.50	0.98	0.50	0.98
	Congestive heart failure (<1 month)	9	0	37	4	0.84	0.82	0.92	0.77	0.69	1.00	1.00	0.90
	History of inflammatory bowel disease	0	0	50	0	1.00	NA	1.00	NA	NA	1.00	NA	1.00
	Medical patient currently at bed rest	0	8	42	0	0.68	NA	0.84	0.00	NA	0.84	0.00	1.00
	Arthroscopic surgery	0	0	50	0	1.00	NA	1.00	NA	NA	1.00	NA	1.00
	Major surgery (>45 minutes)	16	3	27	4	0.72	0.82	0.86	0.71	0.80	0.90	0.84	0.87
	Laparoscopic surgery (>45 minutes)	4	3	43	0	0.88	0.73	0.94	0.70	1.00	0.93	0.57	1.00
	Malignancy (present or previous)	17	0	32	1	0.96	0.97	0.98	0.96	0.94	1.00	1.00	0.97
	Patient confined to bed (>72 hours)	17	0	2	31	-0.24	0.52	0.38	0.04	0.35	1.00	1.00	0.06
	Immobilizing plaster cast (<1 month)	4	0	45	1	0.96	0.89	0.98	0.88	0.80	1.00	1.00	0.98
	central venous access	34	1	12	3	0.84	0.94	0.92	0.80	0.92	0.92	0.97	0.80
	History of DVT/PE	0	2	48	0	0.92	NA	0.96	0.00	NA	0.96	0.00	1.00
	Family history of thrombosis	0	0	50	0	1.00	NA	1.00	NA	NA	1.00	NA	1.00
	Positive Factor V Leiden	0	0	50	0	1.00	NA	1.00	NA	NA	1.00	NA	1.00

prompt	Items	TP	FP	TN	FN	PABAK	F1	Accuracy	Kappa	Sensitivity	Specificity	PPV	NPV
	Positive Prothrombin 20210A	0	0	50	0	1.00	NA	1.00	NA	NA	1.00	NA	1.00
	Positive lupus anticoagulant	0	0	50	0	1.00	NA	1.00	NA	NA	1.00	NA	1.00
	Elevated anticardiolipin antibodies	0	0	50	0	1.00	NA	1.00	NA	NA	1.00	NA	1.00
	Elevated serum homocysteine	4	0	46	0	1.00	1.00	1.00	1.00	1.00	1.00	1.00	1.00
	Heparin-induced thrombocytopenia (HIT)	0	0	50	0	1.00	NA	1.00	NA	NA	1.00	NA	1.00
	Other congenital or acquired thrombophilia	0	1	49	0	0.96	NA	0.98	0.00	NA	0.98	0.00	1.00
	Stroke (<1 month)	9	1	36	4	0.80	0.78	0.90	0.72	0.69	0.97	0.90	0.90
	Elective major lower extremity arthroplasty	0	0	50	0	1.00	NA	1.00	NA	NA	1.00	NA	1.00
	Hip, pelvis or leg fracture (<1 month)	6	0	40	4	0.84	0.75	0.92	0.71	0.60	1.00	1.00	0.91
	Acute spinal cord injury (paralysis) (<1 month)	0	0	50	0	1.00	NA	1.00	NA	NA	1.00	NA	1.00
	Multiple trauma (<1 month)	7	1	39	3	0.84	0.78	0.92	0.73	0.70	0.97	0.88	0.93
	Age (41-60 years)	11	1	38	0	0.96	0.96	0.98	0.94	1.00	0.97	0.92	1.00
	Age (60-74 years)	21	0	27	2	0.92	0.95	0.96	0.92	0.91	1.00	1.00	0.93
	Age (≥75 years)	6	1	43	0	0.96	0.92	0.98	0.91	1.00	0.98	0.86	1.00
	Minor surgery planned	6	1	38	5	0.76	0.67	0.88	0.60	0.55	0.97	0.86	0.88
	Obesity (BMI>25)	10	0	37	3	0.88	0.87	0.94	0.83	0.77	1.00	1.00	0.92
	Swollen legs (current)	6	1	33	10	0.56	0.52	0.78	0.41	0.38	0.97	0.86	0.77
**Expert prompt**	Varicose veins	0	0	50	0	1.00	NA	1.00	NA	NA	1.00	NA	1.00
	Pregnancy or postpartum (<1 month)	0	0	50	0	1.00	NA	1.00	NA	NA	1.00	NA	1.00
	History of unexplained stillborn infant, recurrent spontaneous abortion (≥3), premature birth with toxemia or growth-restricted infant	0	0	50	0	1.00	NA	1.00	NA	NA	1.00	NA	1.00
	Oral contraceptives or hormone replacement therapy	0	1	49	0	0.96	NA	0.98	0.00	NA	0.98	0.00	1.00
	Sepsis (<1 month)	6	0	36	8	0.68	0.60	0.84	0.52	0.43	1.00	1.00	0.82

prompt	Items	TP	FP	TN	FN	PABAK	F1	Accuracy	Kappa	Sensitivity	Specificity	PPV	NPV
	Serious lung disease incl.pneumonia (<1 month)	12	0	18	20	0.20	0.55	0.60	0.30	0.38	1.00	1.00	0.47
	Abnormal pulmonary function	7	0	23	20	0.20	0.41	0.60	0.24	0.26	1.00	1.00	0.53
	Acute myocardial infarction	1	0	48	1	0.96	0.67	0.98	0.66	0.50	1.00	1.00	0.98
	Congestive heart failure (<1 month)	9	0	37	4	0.84	0.82	0.92	0.77	0.69	1.00	1.00	0.90
	History of inflammatory bowel disease	0	0	50	0	1.00	NA	1.00	NA	NA	1.00	NA	1.00
	Medical patient currently at bed rest	0	0	50	0	1.00	NA	1.00	NA	NA	1.00	NA	1.00
	Arthroscopic surgery	0	0	50	0	1.00	NA	1.00	NA	NA	1.00	NA	1.00
	Major surgery (>45 minutes)	17	1	29	3	0.84	0.89	0.92	0.83	0.85	0.97	0.94	0.91
	Laparoscopic surgery (>45 minutes)	4	1	45	0	0.96	0.89	0.98	0.88	1.00	0.98	0.80	1.00
	Malignancy (present or previous)	18	2	30	0	0.92	0.95	0.96	0.92	1.00	0.94	0.90	1.00
	Patient confined to bed (>72 hours)	11	0	2	37	-0.48	0.37	0.26	0.02	0.23	1.00	1.00	0.05
	Immobilizing plaster cast (<1 month)	4	0	45	1	0.96	0.89	0.98	0.88	0.80	1.00	1.00	0.98
	central venous access	27	1	12	10	0.56	0.83	0.78	0.53	0.73	0.92	0.96	0.55
	History of DVT/PE	0	3	47	0	0.88	NA	0.94	0.00	NA	0.94	0.00	1.00
	Family history of thrombosis	0	0	50	0	1.00	NA	1.00	NA	NA	1.00	NA	1.00
	Positive Factor V Leiden	0	0	50	0	1.00	NA	1.00	NA	NA	1.00	NA	1.00
	Positive Prothrombin 20210A	0	0	50	0	1.00	NA	1.00	NA	NA	1.00	NA	1.00
	Positive lupus anticoagulant	0	0	50	0	1.00	NA	1.00	NA	NA	1.00	NA	1.00
	Elevated anticardiolipin antibodies	0	0	50	0	1.00	NA	1.00	NA	NA	1.00	NA	1.00
	Elevated serum homocysteine	4	0	46	0	1.00	1.00	1.00	1.00	1.00	1.00	1.00	1.00
	Heparin-induced thrombocytopenia (HIT)	0	0	50	0	1.00	NA	1.00	NA	NA	1.00	NA	1.00
	Other congenital or acquired thrombophilia	0	1	49	0	0.96	NA	0.98	0.00	NA	0.98	0.00	1.00
	Stroke (<1 month)	6	2	35	7	0.64	0.57	0.82	0.47	0.46	0.95	0.75	0.83
	Elective major lower extremity arthroplasty	0	0	50	0	1.00	NA	1.00	NA	NA	1.00	NA	1.00

prompt	Items	TP	FP	TN	FN	PABAK	F1	Accuracy	Kappa	Sensitivity	Specificity	PPV	NPV
	Hip, pelvis or leg fracture (<1 month)	7	0	40	3	0.88	0.82	0.94	0.79	0.70	1.00	1.00	0.93
	Acute spinal cord injury (paralysis) (<1 month)	0	0	50	0	1.00	NA	1.00	NA	NA	1.00	NA	1.00
	Multiple trauma (<1 month)	9	2	38	1	0.88	0.86	0.94	0.82	0.90	0.95	0.82	0.97

Abbreviations: TP, true positive; FP, false positive; TN, true negative; FN, false negative; Kappa, Cohen's Kappa coefficient; PABAK, Prevalence-Adjusted Bias-Adjusted Kappa; PPV, positive predictive value; NPV, negative predictive value; F1, F1-score; DVT, deep vein thrombosis; PE, pulmonary embolism; BMI, body mass index.

Supplementary Table S13. Performance of GLM-4-9B with Different Prompting Strategies for Item-Level Caprini Score Extraction in the Development Dataset

prompt	Items	TP	FP	TN	FN	PABAK	F1	Accuracy	Kappa	Sensitivity	Specificity	PPV	NPV
Basic prompt	Age (41-60 years)	10	4	35	1	0.80	0.80	0.90	0.73	0.91	0.90	0.71	0.97
	Age (60-74 years)	13	2	25	10	0.52	0.68	0.76	0.50	0.57	0.93	0.87	0.71
	Age (≥75 years)	6	7	37	0	0.72	0.63	0.86	0.56	1.00	0.84	0.46	1.00
	Minor surgery planned	4	8	31	7	0.40	0.35	0.70	0.15	0.36	0.79	0.33	0.82
	Obesity (BMI>25)	9	8	29	4	0.52	0.60	0.76	0.43	0.69	0.78	0.53	0.88
	Swollen legs (current)	7	1	33	9	0.60	0.58	0.80	0.47	0.44	0.97	0.88	0.79
	Varicose veins	0	1	49	0	0.96	NA	0.98	0.00	NA	0.98	0.00	1.00
	Pregnancy or postpartum (<1 month)	0	0	50	0	1.00	NA	1.00	NA	NA	1.00	NA	1.00
	History of unexplained stillborn infant, recurrent spontaneous abortion (≥3), premature birth with toxemia or growth-restricted infant	0	0	50	0	1.00	NA	1.00	NA	NA	1.00	NA	1.00
	Oral contraceptives or hormone replacement therapy	0	0	50	0	1.00	NA	1.00	NA	NA	1.00	NA	1.00
	Sepsis (<1 month)	5	0	36	9	0.64	0.53	0.82	0.44	0.36	1.00	1.00	0.80
	Serious lung disease incl.pneumonia (<1 month)	11	3	15	21	0.04	0.48	0.52	0.15	0.34	0.83	0.79	0.42
	Abnormal pulmonary function	0	0	23	27	-0.08	NA	0.46	0.00	0.00	1.00	NA	0.46
	Acute myocardial infarction	1	1	47	1	0.92	0.50	0.96	0.48	0.50	0.98	0.50	0.98
	Congestive heart failure (<1 month)	7	0	37	6	0.76	0.70	0.88	0.63	0.54	1.00	1.00	0.86
	History of inflammatory bowel disease	0	0	50	0	1.00	NA	1.00	NA	NA	1.00	NA	1.00
	Medical patient currently at bed rest	0	0	50	0	1.00	NA	1.00	NA	NA	1.00	NA	1.00
	Arthroscopic surgery	0	0	50	0	1.00	NA	1.00	NA	NA	1.00	NA	1.00
	Major surgery (>45 minutes)	8	2	28	12	0.44	0.53	0.72	0.36	0.40	0.93	0.80	0.70

prompt	Items	TP	FP	TN	FN	PABAK	F1	Accuracy	Kappa	Sensitivity	Specificity	PPV	NPV
	Laparoscopic surgery (>45 minutes)	1	1	45	3	0.84	0.33	0.92	0.30	0.25	0.98	0.50	0.94
	Malignancy (present or previous)	15	1	31	3	0.84	0.88	0.92	0.82	0.83	0.97	0.94	0.91
	Patient confined to bed (>72 hours)	4	0	2	44	-0.76	0.15	0.12	0.01	0.08	1.00	1.00	0.04
	Immobilizing plaster cast (<1 month)	2	0	45	3	0.88	0.57	0.94	0.55	0.40	1.00	1.00	0.94
	central venous access	15	0	13	22	0.12	0.58	0.56	0.26	0.41	1.00	1.00	0.37
	History of DVT/PE	0	3	47	0	0.88	NA	0.94	0.00	NA	0.94	0.00	1.00
	Family history of thrombosis	0	0	50	0	1.00	NA	1.00	NA	NA	1.00	NA	1.00
	Positive Factor V Leiden	0	0	50	0	1.00	NA	1.00	NA	NA	1.00	NA	1.00
	Positive Prothrombin 20210A	0	0	50	0	1.00	NA	1.00	NA	NA	1.00	NA	1.00
	Positive lupus anticoagulant	0	0	50	0	1.00	NA	1.00	NA	NA	1.00	NA	1.00
	Elevated anticardiolipin antibodies	0	0	50	0	1.00	NA	1.00	NA	NA	1.00	NA	1.00
	Elevated serum homocysteine	0	0	46	4	0.84	NA	0.92	0.00	0.00	1.00	NA	0.92
	Heparin-induced thrombocytopenia (HIT)	0	0	50	0	1.00	NA	1.00	NA	NA	1.00	NA	1.00
	Other congenital or acquired thrombophilia	0	1	49	0	0.96	NA	0.98	0.00	NA	0.98	0.00	1.00
	Stroke (<1 month)	4	0	37	9	0.64	0.47	0.82	0.40	0.31	1.00	1.00	0.80
	Elective major lower extremity arthroplasty	0	0	50	0	1.00	NA	1.00	NA	NA	1.00	NA	1.00
	Hip, pelvis or leg fracture (<1 month)	8	1	39	2	0.88	0.84	0.94	0.81	0.80	0.97	0.89	0.95
	Acute spinal cord injury (paralysis) (<1 month)	0	0	50	0	1.00	NA	1.00	NA	NA	1.00	NA	1.00
	Multiple trauma (<1 month)	4	0	40	6	0.76	0.57	0.88	0.52	0.40	1.00	1.00	0.87
	Age (41-60 years)	11	6	33	0	0.76	0.79	0.88	0.71	1.00	0.85	0.65	1.00
	Age (60-74 years)	18	2	25	5	0.72	0.84	0.86	0.72	0.78	0.93	0.90	0.83
**Complex prompt**	Age (≥75 years)	6	4	40	0	0.84	0.75	0.92	0.71	1.00	0.91	0.60	1.00
	Minor surgery planned	0	4	35	11	0.40	NA	0.70	-0.13	0.00	0.90	0.00	0.76
	Obesity (BMI>25)	10	5	32	3	0.68	0.71	0.84	0.60	0.77	0.86	0.67	0.91
	Swollen legs (current)	3	0	34	13	0.48	0.32	0.74	0.24	0.19	1.00	1.00	0.72

prompt	Items	TP	FP	TN	FN	PABAK	F1	Accuracy	Kappa	Sensitivity	Specificity	PPV	NPV
	Varicose veins	0	0	50	0	1.00	NA	1.00	NA	NA	1.00	NA	1.00
	Pregnancy or postpartum (<1 month)	0	0	50	0	1.00	NA	1.00	NA	NA	1.00	NA	1.00
	History of unexplained stillborn infant, recurrent spontaneous abortion (≥3), premature birth with toxemia or growth-restricted infant	0	0	50	0	1.00	NA	1.00	NA	NA	1.00	NA	1.00
	Oral contraceptives or hormone replacement therapy	0	0	50	0	1.00	NA	1.00	NA	NA	1.00	NA	1.00
	Sepsis (<1 month)	1	0	36	13	0.48	0.13	0.74	0.10	0.07	1.00	1.00	0.73
	Serious lung disease incl.pneumonia (<1 month)	2	0	18	30	-0.20	0.12	0.40	0.05	0.06	1.00	1.00	0.38
	Abnormal pulmonary function	0	0	23	27	-0.08	NA	0.46	0.00	0.00	1.00	NA	0.46
	Acute myocardial infarction	0	0	48	2	0.92	NA	0.96	0.00	0.00	1.00	NA	0.96
	Congestive heart failure (<1 month)	1	0	37	12	0.52	0.14	0.76	0.11	0.08	1.00	1.00	0.76
	History of inflammatory bowel disease	0	0	50	0	1.00	NA	1.00	NA	NA	1.00	NA	1.00
	Medical patient currently at bed rest	0	0	50	0	1.00	NA	1.00	NA	NA	1.00	NA	1.00
	Arthroscopic surgery	0	0	50	0	1.00	NA	1.00	NA	NA	1.00	NA	1.00
	Major surgery (>45 minutes)	5	0	30	15	0.40	0.40	0.70	0.29	0.25	1.00	1.00	0.67
	Laparoscopic surgery (>45 minutes)	0	0	46	4	0.84	NA	0.92	0.00	0.00	1.00	NA	0.92
	Malignancy (present or previous)	15	1	31	3	0.84	0.88	0.92	0.82	0.83	0.97	0.94	0.91
	Patient confined to bed (>72 hours)	7	0	2	41	-0.64	0.25	0.18	0.01	0.15	1.00	1.00	0.05
	Immobilizing plaster cast (<1 month)	1	0	45	4	0.84	0.33	0.92	0.31	0.20	1.00	1.00	0.92
	central venous access	8	0	13	29	-0.16	0.36	0.42	0.13	0.22	1.00	1.00	0.31
	History of DVT/PE	0	3	47	0	0.88	NA	0.94	0.00	NA	0.94	0.00	1.00
	Family history of thrombosis	0	0	50	0	1.00	NA	1.00	NA	NA	1.00	NA	1.00
	Positive Factor V Leiden	0	0	50	0	1.00	NA	1.00	NA	NA	1.00	NA	1.00

prompt	Items	TP	FP	TN	FN	PABAK	F1	Accuracy	Kappa	Sensitivity	Specificity	PPV	NPV
	Positive Prothrombin 20210A	0	0	50	0	1.00	NA	1.00	NA	NA	1.00	NA	1.00
	Positive lupus anticoagulant	0	0	50	0	1.00	NA	1.00	NA	NA	1.00	NA	1.00
	Elevated anticardiolipin antibodies	0	0	50	0	1.00	NA	1.00	NA	NA	1.00	NA	1.00
	Elevated serum homocysteine	0	0	46	4	0.84	NA	0.92	0.00	0.00	1.00	NA	0.92
	Heparin-induced thrombocytopenia (HIT)	0	0	50	0	1.00	NA	1.00	NA	NA	1.00	NA	1.00
	Other congenital or acquired thrombophilia	0	0	50	0	1.00	NA	1.00	NA	NA	1.00	NA	1.00
	Stroke (<1 month)	2	0	37	11	0.56	0.27	0.78	0.21	0.15	1.00	1.00	0.77
	Elective major lower extremity arthroplasty	0	0	50	0	1.00	NA	1.00	NA	NA	1.00	NA	1.00
	Hip, pelvis or leg fracture (<1 month)	3	0	40	7	0.72	0.46	0.86	0.41	0.30	1.00	1.00	0.85
	Acute spinal cord injury (paralysis) (<1 month)	0	0	50	0	1.00	NA	1.00	NA	NA	1.00	NA	1.00
	Multiple trauma (<1 month)	3	0	40	7	0.72	0.46	0.86	0.41	0.30	1.00	1.00	0.85
**Expert prompt**	Age (41-60 years)	9	2	37	2	0.84	0.82	0.92	0.77	0.82	0.95	0.82	0.95
	Age (60-74 years)	18	2	25	5	0.72	0.84	0.86	0.72	0.78	0.93	0.90	0.83
	Age (≥75 years)	6	5	39	0	0.80	0.71	0.90	0.65	1.00	0.89	0.55	1.00
	Minor surgery planned	5	14	25	6	0.20	0.33	0.60	0.08	0.45	0.64	0.26	0.81
	Obesity (BMI>25)	10	1	36	3	0.84	0.83	0.92	0.78	0.77	0.97	0.91	0.92
	Swollen legs (current)	7	0	34	9	0.64	0.61	0.82	0.51	0.44	1.00	1.00	0.79
	Varicose veins	0	1	49	0	0.96	NA	0.98	0.00	NA	0.98	0.00	1.00
	Pregnancy or postpartum (<1 month)	0	0	50	0	1.00	NA	1.00	NA	NA	1.00	NA	1.00
	History of unexplained stillborn infant, recurrent spontaneous abortion (≥3), premature birth with toxemia or growth-restricted infant	0	0	50	0	1.00	NA	1.00	NA	NA	1.00	NA	1.00
	Oral contraceptives or hormone replacement therapy	0	0	50	0	1.00	NA	1.00	NA	NA	1.00	NA	1.00
	Sepsis (<1 month)	2	0	36	12	0.52	0.25	0.76	0.19	0.14	1.00	1.00	0.75

prompt	Items	TP	FP	TN	FN	PABAK	F1	Accuracy	Kappa	Sensitivity	Specificity	PPV	NPV
	Serious lung disease incl.pneumonia (<1 month)	1	0	18	31	-0.24	0.06	0.38	0.02	0.03	1.00	1.00	0.37
	Abnormal pulmonary function	0	0	23	27	-0.08	NA	0.46	0.00	0.00	1.00	NA	0.46
	Acute myocardial infarction	0	0	48	2	0.92	NA	0.96	0.00	0.00	1.00	NA	0.96
	Congestive heart failure (<1 month)	5	0	37	8	0.68	0.56	0.84	0.48	0.38	1.00	1.00	0.82
	History of inflammatory bowel disease	0	0	50	0	1.00	NA	1.00	NA	NA	1.00	NA	1.00
	Medical patient currently at bed rest	0	0	50	0	1.00	NA	1.00	NA	NA	1.00	NA	1.00
	Arthroscopic surgery	0	0	50	0	1.00	NA	1.00	NA	NA	1.00	NA	1.00
	Major surgery (>45 minutes)	5	1	29	15	0.36	0.38	0.68	0.25	0.25	0.97	0.83	0.66
	Laparoscopic surgery (>45 minutes)	0	0	46	4	0.84	NA	0.92	0.00	0.00	1.00	NA	0.92
	Malignancy (present or previous)	9	0	32	9	0.64	0.67	0.82	0.56	0.50	1.00	1.00	0.78
	Patient confined to bed (>72 hours)	1	0	2	47	-0.88	0.04	0.06	0.00	0.02	1.00	1.00	0.04
	Immobilizing plaster cast (<1 month)	1	0	45	4	0.84	0.33	0.92	0.31	0.20	1.00	1.00	0.92
	central venous access	12	0	13	25	0.00	0.49	0.50	0.20	0.32	1.00	1.00	0.34
	History of DVT/PE	0	0	50	0	1.00	NA	1.00	NA	NA	1.00	NA	1.00
	Family history of thrombosis	0	0	50	0	1.00	NA	1.00	NA	NA	1.00	NA	1.00
	Positive Factor V Leiden	0	0	50	0	1.00	NA	1.00	NA	NA	1.00	NA	1.00
	Positive Prothrombin 20210A	0	0	50	0	1.00	NA	1.00	NA	NA	1.00	NA	1.00
	Positive lupus anticoagulant	0	0	50	0	1.00	NA	1.00	NA	NA	1.00	NA	1.00
	Elevated anticardiolipin antibodies	0	0	50	0	1.00	NA	1.00	NA	NA	1.00	NA	1.00
	Elevated serum homocysteine	0	0	46	4	0.84	NA	0.92	0.00	0.00	1.00	NA	0.92
	Heparin-induced thrombocytopenia (HIT)	0	0	50	0	1.00	NA	1.00	NA	NA	1.00	NA	1.00
	Other congenital or acquired thrombophilia	0	0	50	0	1.00	NA	1.00	NA	NA	1.00	NA	1.00
	Stroke (<1 month)	0	0	37	13	0.48	NA	0.74	0.00	0.00	1.00	NA	0.74
	Elective major lower extremity arthroplasty	0	0	50	0	1.00	NA	1.00	NA	NA	1.00	NA	1.00

prompt	Items	TP	FP	TN	FN	PABAK	F1	Accuracy	Kappa	Sensitivity	Specificity	PPV	NPV
	Hip, pelvis or leg fracture (<1 month)	5	0	40	5	0.80	0.67	0.90	0.62	0.50	1.00	1.00	0.89
	Acute spinal cord injury (paralysis) (<1 month)	0	0	50	0	1.00	NA	1.00	NA	NA	1.00	NA	1.00
	Multiple trauma (<1 month)	4	0	40	6	0.76	0.57	0.88	0.52	0.40	1.00	1.00	0.87

Abbreviations: TP, true positive; FP, false positive; TN, true negative; FN, false negative; Kappa, Cohen's Kappa coefficient; PABAK, Prevalence-Adjusted Bias-Adjusted Kappa; PPV, positive predictive value; NPV, negative predictive value; F1, F1-score; DVT, deep vein thrombosis; PE, pulmonary embolism; BMI, body mass index.

Supplementary Table S14. Performance of Qwen3-32B with Different Prompting Strategies for Item-Level Caprini Score Extraction in the Development Dataset

prompt	Items	TP	FP	TN	FN	PABAK	F1	Accuracy	Kappa	Sensitivity	Specificity	PPV	NPV
Basic prompt	Age (41-60 years)	11	1	38	0	0.96	0.96	0.98	0.94	1.00	0.97	0.92	1.00
	Age (60-74 years)	22	0	27	1	0.96	0.98	0.98	0.96	0.96	1.00	1.00	0.96
	Age (≥75 years)	6	0	44	0	1.00	1.00	1.00	1.00	1.00	1.00	1.00	1.00
	Minor surgery planned	2	5	34	9	0.44	0.22	0.72	0.06	0.18	0.87	0.29	0.79
	Obesity (BMI>25)	12	0	37	1	0.96	0.96	0.98	0.95	0.92	1.00	1.00	0.97
	Swollen legs (current)	13	3	31	3	0.76	0.81	0.88	0.72	0.81	0.91	0.81	0.91
	Varicose veins	0	1	49	0	0.96	NA	0.98	NA	NA	0.98	0.00	1.00
	Pregnancy or postpartum (<1 month)	0	0	50	0	1.00	NA	1.00	NA	NA	1.00	NA	1.00
	History of unexplained stillborn infant, recurrent spontaneous abortion (≥3), premature birth with toxemia or growth-restricted infant	0	0	50	0	1.00	NA	1.00	NA	NA	1.00	NA	1.00
	Oral contraceptives or hormone replacement therapy	0	3	47	0	0.88	NA	0.94	NA	NA	0.94	0.00	1.00
	Sepsis (<1 month)	13	6	30	1	0.72	0.79	0.86	0.69	0.93	0.83	0.68	0.97
	Serious lung disease incl.pneumonia (<1 month)	23	2	16	9	0.56	0.81	0.78	0.56	0.72	0.89	0.92	0.64
	Abnormal pulmonary function	4	0	23	23	0.08	0.26	0.54	0.14	0.15	1.00	1.00	0.50
	Acute myocardial infarction	0	0	48	2	0.92	NA	0.96	NA	0.00	1.00	NA	0.96
	Congestive heart failure (<1 month)	11	1	36	2	0.88	0.88	0.94	0.84	0.85	0.97	0.92	0.95
	History of inflammatory bowel disease	0	0	50	0	1.00	NA	1.00	NA	NA	1.00	NA	1.00
	Medical patient currently at bed rest	0	0	50	0	1.00	NA	1.00	NA	NA	1.00	NA	1.00
	Arthroscopic surgery	0	0	50	0	1.00	NA	1.00	NA	NA	1.00	NA	1.00
	Major surgery (>45 minutes)	17	7	23	3	0.60	0.77	0.80	0.60	0.85	0.77	0.71	0.88

prompt	Items	TP	FP	TN	FN	PABAK	F1	Accuracy	Kappa	Sensitivity	Specificity	PPV	NPV
	Laparoscopic surgery (>45 minutes)	4	1	45	0	0.96	0.89	0.98	0.88	1.00	0.98	0.80	1.00
	Malignancy (present or previous)	18	0	32	0	1.00	1.00	1.00	1.00	1.00	1.00	1.00	1.00
	Patient confined to bed (>72 hours)	14	1	1	34	-0.40	0.44	0.30	-0.02	0.29	0.50	0.93	0.03
	Immobilizing plaster cast (<1 month)	2	0	45	3	0.88	0.57	0.94	0.55	0.40	1.00	1.00	0.94
	central venous access	36	3	10	1	0.84	0.95	0.92	0.78	0.97	0.77	0.92	0.91
	History of DVT/PE	0	3	47	0	0.88	NA	0.94	NA	NA	0.94	0.00	1.00
	Family history of thrombosis	0	0	50	0	1.00	NA	1.00	NA	NA	1.00	NA	1.00
	Positive Factor V Leiden	0	0	50	0	1.00	NA	1.00	NA	NA	1.00	NA	1.00
	Positive Prothrombin 20210A	0	0	50	0	1.00	NA	1.00	NA	NA	1.00	NA	1.00
	Positive lupus anticoagulant	0	0	50	0	1.00	NA	1.00	NA	NA	1.00	NA	1.00
	Elevated anticardiolipin antibodies	0	0	50	0	1.00	NA	1.00	NA	NA	1.00	NA	1.00
	Elevated serum homocysteine	3	0	46	1	0.96	0.86	0.98	0.85	0.75	1.00	1.00	0.98
	Heparin-induced thrombocytopenia (HIT)	0	0	50	0	1.00	NA	1.00	NA	NA	1.00	NA	1.00
	Other congenital or acquired thrombophilia	0	2	48	0	0.92	NA	0.96	NA	NA	0.96	0.00	1.00
	Stroke (<1 month)	8	3	34	5	0.68	0.67	0.84	0.56	0.62	0.92	0.73	0.87
	Elective major lower extremity arthroplasty	0	0	50	0	1.00	NA	1.00	NA	NA	1.00	NA	1.00
	Hip, pelvis or leg fracture (<1 month)	7	1	39	3	0.84	0.78	0.92	0.73	0.70	0.97	0.88	0.93
	Acute spinal cord injury (paralysis) (<1 month)	0	0	50	0	1.00	NA	1.00	NA	NA	1.00	NA	1.00
	Multiple trauma (<1 month)	6	0	40	4	0.84	0.75	0.92	0.71	0.60	1.00	1.00	0.91
	Age (41-60 years)	11	1	38	0	0.96	0.96	0.98	0.94	1.00	0.97	0.92	1.00
	Age (60-74 years)	22	0	27	1	0.96	0.98	0.98	0.96	0.96	1.00	1.00	0.96
**Complex prompt**	Age (≥75 years)	6	0	44	0	1.00	1.00	1.00	1.00	1.00	1.00	1.00	1.00
	Minor surgery planned	2	2	37	9	0.56	0.27	0.78	0.17	0.18	0.95	0.50	0.80
	Obesity (BMI>25)	12	1	36	1	0.92	0.92	0.96	0.90	0.92	0.97	0.92	0.97
	Swollen legs (current)	15	2	32	1	0.88	0.91	0.94	0.86	0.94	0.94	0.88	0.97

prompt	Items	TP	FP	TN	FN	PABAK	F1	Accuracy	Kappa	Sensitivity	Specificity	PPV	NPV
	Varicose veins	0	1	49	0	0.96	NA	0.98	0.00	NA	0.98	0.00	1.00
	Pregnancy or postpartum (<1 month)	0	0	50	0	1.00	NA	1.00	NA	NA	1.00	NA	1.00
	History of unexplained stillborn infant, recurrent spontaneous abortion (≥3), premature birth with toxemia or growth-restricted infant	0	0	50	0	1.00	NA	1.00	NA	NA	1.00	NA	1.00
	Oral contraceptives or hormone replacement therapy	0	0	50	0	1.00	NA	1.00	NA	NA	1.00	NA	1.00
	Sepsis (<1 month)	11	1	35	3	0.84	0.85	0.92	0.79	0.79	0.97	0.92	0.92
	Serious lung disease incl.pneumonia (<1 month)	17	0	18	15	0.40	0.69	0.70	0.45	0.53	1.00	1.00	0.55
	Abnormal pulmonary function	15	2	21	12	0.44	0.68	0.72	0.45	0.56	0.91	0.88	0.64
	Acute myocardial infarction	1	0	48	1	0.96	0.67	0.98	0.66	0.50	1.00	1.00	0.98
	Congestive heart failure (<1 month)	10	1	36	3	0.84	0.83	0.92	0.78	0.77	0.97	0.91	0.92
	History of inflammatory bowel disease	0	0	50	0	1.00	NA	1.00	NA	NA	1.00	NA	1.00
	Medical patient currently at bed rest	0	1	49	0	0.96	NA	0.98	0.00	NA	0.98	0.00	1.00
	Arthroscopic surgery	0	0	50	0	1.00	NA	1.00	NA	NA	1.00	NA	1.00
	Major surgery (>45 minutes)	18	7	23	2	0.64	0.80	0.82	0.64	0.90	0.77	0.72	0.92
	Laparoscopic surgery (>45 minutes)	3	0	46	1	0.96	0.86	0.98	0.85	0.75	1.00	1.00	0.98
	Malignancy (present or previous)	17	1	31	1	0.92	0.94	0.96	0.91	0.94	0.97	0.94	0.97
	Patient confined to bed (>72 hours)	10	0	2	38	-0.52	0.34	0.24	0.02	0.21	1.00	1.00	0.05
	Immobilizing plaster cast (<1 month)	2	0	45	3	0.88	0.57	0.94	0.55	0.40	1.00	1.00	0.94
	central venous access	34	1	12	3	0.84	0.94	0.92	0.80	0.92	0.92	0.97	0.80
	History of DVT/PE	0	10	40	0	0.60	NA	0.80	0.00	NA	0.80	0.00	1.00
	Family history of thrombosis	0	0	50	0	1.00	NA	1.00	NA	NA	1.00	NA	1.00
	Positive Factor V Leiden	0	0	50	0	1.00	NA	1.00	NA	NA	1.00	NA	1.00

prompt	Items	TP	FP	TN	FN	PABAK	F1	Accuracy	Kappa	Sensitivity	Specificity	PPV	NPV
	Positive Prothrombin 20210A	0	0	50	0	1.00	NA	1.00	NA	NA	1.00	NA	1.00
	Positive lupus anticoagulant	0	0	50	0	1.00	NA	1.00	NA	NA	1.00	NA	1.00
	Elevated anticardiolipin antibodies	0	0	50	0	1.00	NA	1.00	NA	NA	1.00	NA	1.00
	Elevated serum homocysteine	4	0	46	0	1.00	1.00	1.00	1.00	1.00	1.00	1.00	1.00
	Heparin-induced thrombocytopenia (HIT)	0	1	49	0	0.96	NA	0.98	0.00	NA	0.98	0.00	1.00
	Other congenital or acquired thrombophilia	0	1	49	0	0.96	NA	0.98	0.00	NA	0.98	0.00	1.00
	Stroke (<1 month)	6	1	36	7	0.68	0.60	0.84	0.51	0.46	0.97	0.86	0.84
	Elective major lower extremity arthroplasty	0	0	50	0	1.00	NA	1.00	NA	NA	1.00	NA	1.00
	Hip, pelvis or leg fracture (<1 month)	7	1	39	3	0.84	0.78	0.92	0.73	0.70	0.97	0.88	0.93
	Acute spinal cord injury (paralysis) (<1 month)	0	0	50	0	1.00	NA	1.00	NA	NA	1.00	NA	1.00
	Multiple trauma (<1 month)	7	1	39	3	0.84	0.78	0.92	0.73	0.70	0.97	0.88	0.93
**Expert prompt**	Age (41-60 years)	11	1	38	0	0.96	0.96	0.98	0.94	1.00	0.97	0.92	1.00
	Age (60-74 years)	22	1	26	1	0.92	0.96	0.96	0.92	0.96	0.96	0.96	0.96
	Age (≥75 years)	6	0	44	0	1.00	1.00	1.00	1.00	1.00	1.00	1.00	1.00
	Minor surgery planned	5	3	36	6	0.64	0.53	0.82	0.42	0.45	0.92	0.62	0.86
	Obesity (BMI>25)	12	0	37	1	0.96	0.96	0.98	0.95	0.92	1.00	1.00	0.97
	Swollen legs (current)	9	1	33	7	0.68	0.69	0.84	0.59	0.56	0.97	0.90	0.82
	Varicose veins	0	2	48	0	0.92	NA	0.96	0.00	NA	0.96	0.00	1.00
	Pregnancy or postpartum (<1 month)	0	0	50	0	1.00	NA	1.00	NA	NA	1.00	NA	1.00
	History of unexplained stillborn infant, recurrent spontaneous abortion (≥3), premature birth with toxemia or growth-restricted infant	0	0	50	0	1.00	NA	1.00	NA	NA	1.00	NA	1.00
	Oral contraceptives or hormone replacement therapy	0	1	49	0	0.96	NA	0.98	0.00	NA	0.98	0.00	1.00
	Sepsis (<1 month)	9	1	35	5	0.76	0.75	0.88	0.67	0.64	0.97	0.90	0.88

prompt	Items	TP	FP	TN	FN	PABAK	F1	Accuracy	Kappa	Sensitivity	Specificity	PPV	NPV
	Serious lung disease incl.pneumonia (<1 month)	11	0	18	21	0.16	0.51	0.58	0.27	0.34	1.00	1.00	0.46
	Abnormal pulmonary function	12	1	22	15	0.36	0.60	0.68	0.38	0.44	0.96	0.92	0.59
	Acute myocardial infarction	1	0	48	1	0.96	0.67	0.98	0.66	0.50	1.00	1.00	0.98
	Congestive heart failure (<1 month)	13	0	37	0	1.00	1.00	1.00	1.00	1.00	1.00	1.00	1.00
	History of inflammatory bowel disease	0	0	50	0	1.00	NA	1.00	NA	NA	1.00	NA	1.00
	Medical patient currently at bed rest	0	0	50	0	1.00	NA	1.00	NA	NA	1.00	NA	1.00
	Arthroscopic surgery	0	0	50	0	1.00	NA	1.00	NA	NA	1.00	NA	1.00
	Major surgery (>45 minutes)	17	2	28	3	0.80	0.87	0.90	0.79	0.85	0.93	0.89	0.90
	Laparoscopic surgery (>45 minutes)	2	0	46	2	0.92	0.67	0.96	0.65	0.50	1.00	1.00	0.96
	Malignancy (present or previous)	17	2	30	1	0.88	0.92	0.94	0.87	0.94	0.94	0.89	0.97
	Patient confined to bed (>72 hours)	12	0	2	36	-0.44	0.40	0.28	0.03	0.25	1.00	1.00	0.05
	Immobilizing plaster cast (<1 month)	2	1	44	3	0.84	0.50	0.92	0.46	0.40	0.98	0.67	0.94
	central venous access	34	3	10	3	0.76	0.92	0.88	0.69	0.92	0.77	0.92	0.77
	History of DVT/PE	0	6	44	0	0.76	NA	0.88	0.00	NA	0.88	0.00	1.00
	Family history of thrombosis	0	0	50	0	1.00	NA	1.00	NA	NA	1.00	NA	1.00
	Positive Factor V Leiden	0	0	50	0	1.00	NA	1.00	NA	NA	1.00	NA	1.00
	Positive Prothrombin 20210A	0	0	50	0	1.00	NA	1.00	NA	NA	1.00	NA	1.00
	Positive lupus anticoagulant	0	0	50	0	1.00	NA	1.00	NA	NA	1.00	NA	1.00
	Elevated anticardiolipin antibodies	0	0	50	0	1.00	NA	1.00	NA	NA	1.00	NA	1.00
	Elevated serum homocysteine	3	0	46	1	0.96	0.86	0.98	0.85	0.75	1.00	1.00	0.98
	Heparin-induced thrombocytopenia (HIT)	0	0	50	0	1.00	NA	1.00	NA	NA	1.00	NA	1.00
	Other congenital or acquired thrombophilia	0	0	50	0	1.00	NA	1.00	NA	NA	1.00	NA	1.00
	Stroke (<1 month)	5	1	36	8	0.64	0.53	0.82	0.43	0.38	0.97	0.83	0.82
	Elective major lower extremity arthroplasty	0	0	50	0	1.00	NA	1.00	NA	NA	1.00	NA	1.00

prompt	Items	TP	FP	TN	FN	PABAK	F1	Accuracy	Kappa	Sensitivity	Specificity	PPV	NPV
	Hip, pelvis or leg fracture (<1 month)	5	0	40	5	0.80	0.67	0.90	0.62	0.50	1.00	1.00	0.89
	Acute spinal cord injury (paralysis) (<1 month)	0	0	50	0	1.00	NA	1.00	NA	NA	1.00	NA	1.00
	Multiple trauma (<1 month)	4	3	37	6	0.64	0.47	0.82	0.37	0.40	0.92	0.57	0.86

Abbreviations: TP, true positive; FP, false positive; TN, true negative; FN, false negative; Kappa, Cohen's Kappa coefficient; PABAK, Prevalence-Adjusted Bias-Adjusted Kappa; PPV, positive predictive value; NPV, negative predictive value; F1, F1-score; DVT, deep vein thrombosis; PE, pulmonary embolism; BMI, body mass index.

Supplementary Table S15. Performance of Qwen3-Next with Different Prompting Strategies for Item-Level Caprini Score Extraction in the Development Dataset

prompt	Items	TP	FP	TN	FN	PABAK	F1	Accuracy	Kappa	Sensitivity	Specificity	PPV	NPV
**Basic prompt**	Age (41-60 years)	11	1	38	0	0.96	0.96	0.98	0.94	1.00	0.97	0.92	1.00
	Age (60-74 years)	22	0	27	1	0.96	0.98	0.98	0.96	0.96	1.00	1.00	0.96
	Age (≥75 years)	6	0	44	0	1.00	1.00	1.00	1.00	1.00	1.00	1.00	1.00
	Minor surgery planned	1	3	36	10	0.48	0.13	0.74	0.02	0.09	0.92	0.25	0.78
	Obesity (BMI>25)	12	1	36	1	0.92	0.92	0.96	0.90	0.92	0.97	0.92	0.97
	Swollen legs (current)	10	2	32	6	0.68	0.71	0.84	0.61	0.62	0.94	0.83	0.84
	Varicose veins	0	0	50	0	1.00	NA	1.00	NA	NA	1.00	NA	1.00
	Pregnancy or postpartum (<1 month)	0	0	50	0	1.00	NA	1.00	NA	NA	1.00	NA	1.00
	History of unexplained stillborn infant, recurrent spontaneous abortion (≥3), premature birth with toxemia or growth-restricted infant	0	0	50	0	1.00	NA	1.00	NA	NA	1.00	NA	1.00
	Oral contraceptives or hormone replacement therapy	0	2	48	0	0.92	NA	0.96	0.00	NA	0.96	0.00	1.00
	Sepsis (<1 month)	9	12	24	5	0.32	0.51	0.66	0.27	0.64	0.67	0.43	0.83
	Serious lung disease incl.pneumonia (<1 month)	17	0	18	15	0.40	0.69	0.70	0.45	0.53	1.00	1.00	0.55
	Abnormal pulmonary function	7	1	22	20	0.16	0.40	0.58	0.20	0.26	0.96	0.88	0.52
	Acute myocardial infarction	0	1	47	2	0.88	NA	0.94	-0.03	0.00	0.98	0.00	0.96
	Congestive heart failure (<1 month)	8	0	37	5	0.80	0.76	0.90	0.70	0.62	1.00	1.00	0.88
	History of inflammatory bowel disease	0	0	50	0	1.00	NA	1.00	NA	NA	1.00	NA	1.00
	Medical patient currently at bed rest	0	0	50	0	1.00	NA	1.00	NA	NA	1.00	NA	1.00
	Arthroscopic surgery	0	0	50	0	1.00	NA	1.00	NA	NA	1.00	NA	1.00
	Major surgery (>45 minutes)	18	1	29	2	0.88	0.92	0.94	0.87	0.90	0.97	0.95	0.94

prompt	Items	TP	FP	TN	FN	PABAK	F1	Accuracy	Kappa	Sensitivity	Specificity	PPV	NPV
	Laparoscopic surgery (>45 minutes)	3	2	44	1	0.88	0.67	0.94	0.63	0.75	0.96	0.60	0.98
	Malignancy (present or previous)	18	2	30	0	0.92	0.95	0.96	0.92	1.00	0.94	0.90	1.00
	Patient confined to bed (>72 hours)	42	1	1	6	0.72	0.92	0.86	0.17	0.88	0.50	0.98	0.14
	Immobilizing plaster cast (<1 month)	4	0	45	1	0.96	0.89	0.98	0.88	0.80	1.00	1.00	0.98
	central venous access	36	6	7	1	0.72	0.91	0.86	0.58	0.97	0.54	0.86	0.88
	History of DVT/PE	0	8	42	0	0.68	NA	0.84	0.00	NA	0.84	0.00	1.00
	Family history of thrombosis	0	0	50	0	1.00	NA	1.00	NA	NA	1.00	NA	1.00
	Positive Factor V Leiden	0	0	50	0	1.00	NA	1.00	NA	NA	1.00	NA	1.00
	Positive Prothrombin 20210A	0	0	50	0	1.00	NA	1.00	NA	NA	1.00	NA	1.00
	Positive lupus anticoagulant	0	0	50	0	1.00	NA	1.00	NA	NA	1.00	NA	1.00
	Elevated anticardiolipin antibodies	0	0	50	0	1.00	NA	1.00	NA	NA	1.00	NA	1.00
	Elevated serum homocysteine	4	0	46	0	1.00	1.00	1.00	1.00	1.00	1.00	1.00	1.00
	Heparin-induced thrombocytopenia (HIT)	0	0	50	0	1.00	NA	1.00	NA	NA	1.00	NA	1.00
	Other congenital or acquired thrombophilia	0	0	50	0	1.00	NA	1.00	NA	NA	1.00	NA	1.00
	Stroke (<1 month)	7	5	32	6	0.56	0.56	0.78	0.41	0.54	0.86	0.58	0.84
	Elective major lower extremity arthroplasty	0	0	50	0	1.00	NA	1.00	NA	NA	1.00	NA	1.00
	Hip, pelvis or leg fracture (<1 month)	8	1	39	2	0.88	0.84	0.94	0.81	0.80	0.97	0.89	0.95
	Acute spinal cord injury (paralysis) (<1 month)	0	0	50	0	1.00	NA	1.00	NA	NA	1.00	NA	1.00
	Multiple trauma (<1 month)	7	1	39	3	0.84	0.78	0.92	0.73	0.70	0.97	0.88	0.93
	Age (41-60 years)	11	1	38	0	0.96	0.96	0.98	0.94	1.00	0.97	0.92	1.00
	Age (60-74 years)	22	1	26	1	0.92	0.96	0.96	0.92	0.96	0.96	0.96	0.96
**Complex prompt**	Age (≥75 years)	6	0	44	0	1.00	1.00	1.00	1.00	1.00	1.00	1.00	1.00
	Minor surgery planned	1	2	37	10	0.52	0.14	0.76	0.05	0.09	0.95	0.33	0.79
	Obesity (BMI>25)	12	1	36	1	0.92	0.92	0.96	0.90	0.92	0.97	0.92	0.97
	Swollen legs (current)	12	1	33	4	0.80	0.83	0.90	0.76	0.75	0.97	0.92	0.89

prompt	Items	TP	FP	TN	FN	PABAK	F1	Accuracy	Kappa	Sensitivity	Specificity	PPV	NPV
	Varicose veins	0	0	50	0	1.00	NA	1.00	NA	NA	1.00	NA	1.00
	Pregnancy or postpartum (<1 month)	0	0	50	0	1.00	NA	1.00	NA	NA	1.00	NA	1.00
	History of unexplained stillborn infant, recurrent spontaneous abortion (≥3), premature birth with toxemia or growth-restricted infant	0	0	50	0	1.00	NA	1.00	NA	NA	1.00	NA	1.00
	Oral contraceptives or hormone replacement therapy	0	2	48	0	0.92	NA	0.96	0.00	NA	0.96	0.00	1.00
	Sepsis (<1 month)	13	8	28	1	0.64	0.74	0.82	0.61	0.93	0.78	0.62	0.97
	Serious lung disease incl.pneumonia (<1 month)	26	1	17	6	0.72	0.88	0.86	0.71	0.81	0.94	0.96	0.74
	Abnormal pulmonary function	17	0	23	10	0.60	0.77	0.80	0.61	0.63	1.00	1.00	0.70
	Acute myocardial infarction	1	0	48	1	0.96	0.67	0.98	0.66	0.50	1.00	1.00	0.98
	Congestive heart failure (<1 month)	12	2	35	1	0.88	0.89	0.94	0.85	0.92	0.95	0.86	0.97
	History of inflammatory bowel disease	0	0	50	0	1.00	NA	1.00	NA	NA	1.00	NA	1.00
	Medical patient currently at bed rest	0	1	49	0	0.96	NA	0.98	0.00	NA	0.98	0.00	1.00
	Arthroscopic surgery	0	0	50	0	1.00	NA	1.00	NA	NA	1.00	NA	1.00
	Major surgery (>45 minutes)	17	3	27	3	0.76	0.85	0.88	0.75	0.85	0.90	0.85	0.90
	Laparoscopic surgery (>45 minutes)	2	1	45	2	0.88	0.57	0.94	0.54	0.50	0.98	0.67	0.96
	Malignancy (present or previous)	18	3	29	0	0.88	0.92	0.94	0.87	1.00	0.91	0.86	1.00
	Patient confined to bed (>72 hours)	47	1	1	1	0.92	0.98	0.96	0.48	0.98	0.50	0.98	0.50
	Immobilizing plaster cast (<1 month)	4	0	45	1	0.96	0.89	0.98	0.88	0.80	1.00	1.00	0.98
	central venous access	37	6	7	0	0.76	0.92	0.88	0.63	1.00	0.54	0.86	1.00
	History of DVT/PE	0	8	42	0	0.68	NA	0.84	0.00	NA	0.84	0.00	1.00
	Family history of thrombosis	0	0	50	0	1.00	NA	1.00	NA	NA	1.00	NA	1.00
	Positive Factor V Leiden	0	0	50	0	1.00	NA	1.00	NA	NA	1.00	NA	1.00

prompt	Items	TP	FP	TN	FN	PABAK	F1	Accuracy	Kappa	Sensitivity	Specificity	PPV	NPV
	Positive Prothrombin 20210A	0	0	50	0	1.00	NA	1.00	NA	NA	1.00	NA	1.00
	Positive lupus anticoagulant	0	0	50	0	1.00	NA	1.00	NA	NA	1.00	NA	1.00
	Elevated anticardiolipin antibodies	0	0	50	0	1.00	NA	1.00	NA	NA	1.00	NA	1.00
	Elevated serum homocysteine	4	0	46	0	1.00	1.00	1.00	1.00	1.00	1.00	1.00	1.00
	Heparin-induced thrombocytopenia (HIT)	0	0	50	0	1.00	NA	1.00	NA	NA	1.00	NA	1.00
	Other congenital or acquired thrombophilia	0	0	50	0	1.00	NA	1.00	NA	NA	1.00	NA	1.00
	Stroke (<1 month)	9	3	34	4	0.72	0.72	0.86	0.63	0.69	0.92	0.75	0.89
	Elective major lower extremity arthroplasty	0	0	50	0	1.00	NA	1.00	NA	NA	1.00	NA	1.00
	Hip, pelvis or leg fracture (<1 month)	7	1	39	3	0.84	0.78	0.92	0.73	0.70	0.97	0.88	0.93
	Acute spinal cord injury (paralysis) (<1 month)	0	0	50	0	1.00	NA	1.00	NA	NA	1.00	NA	1.00
	Multiple trauma (<1 month)	7	1	39	3	0.84	0.78	0.92	0.73	0.70	0.97	0.88	0.93
**Expert prompt**	Age (41-60 years)	7	0	39	4	0.84	0.78	0.92	0.73	0.64	1.00	1.00	0.91
	Age (60-74 years)	23	5	22	0	0.80	0.90	0.90	0.80	1.00	0.81	0.82	1.00
	Age (≥75 years)	6	0	44	0	1.00	1.00	1.00	1.00	1.00	1.00	1.00	1.00
	Minor surgery planned	9	3	36	2	0.80	0.78	0.90	0.72	0.82	0.92	0.75	0.95
	Obesity (BMI>25)	12	0	37	1	0.96	0.96	0.98	0.95	0.92	1.00	1.00	0.97
	Swollen legs (current)	15	2	32	1	0.88	0.91	0.94	0.86	0.94	0.94	0.88	0.97
	Varicose veins	0	0	50	0	1.00	NA	1.00	NA	NA	1.00	NA	1.00
	Pregnancy or postpartum (<1 month)	0	0	50	0	1.00	NA	1.00	NA	NA	1.00	NA	1.00
	History of unexplained stillborn infant, recurrent spontaneous abortion (≥3), premature birth with toxemia or growth-restricted infant	0	0	50	0	1.00	NA	1.00	NA	NA	1.00	NA	1.00
	Oral contraceptives or hormone replacement therapy	0	0	50	0	1.00	NA	1.00	NA	NA	1.00	NA	1.00
	Sepsis (<1 month)	14	0	36	0	1.00	1.00	1.00	1.00	1.00	1.00	1.00	1.00

prompt	Items	TP	FP	TN	FN	PABAK	F1	Accuracy	Kappa	Sensitivity	Specificity	PPV	NPV
	Serious lung disease incl.pneumonia (<1 month)	27	3	15	5	0.68	0.87	0.84	0.66	0.84	0.83	0.90	0.75
	Abnormal pulmonary function	19	0	23	8	0.68	0.83	0.84	0.69	0.70	1.00	1.00	0.74
	Acute myocardial infarction	1	1	47	1	0.92	0.50	0.96	0.48	0.50	0.98	0.50	0.98
	Congestive heart failure (<1 month)	13	1	36	0	0.96	0.96	0.98	0.95	1.00	0.97	0.93	1.00
	History of inflammatory bowel disease	0	0	50	0	1.00	NA	1.00	NA	NA	1.00	NA	1.00
	Medical patient currently at bed rest	0	0	50	0	1.00	NA	1.00	NA	NA	1.00	NA	1.00
	Arthroscopic surgery	0	0	50	0	1.00	NA	1.00	NA	NA	1.00	NA	1.00
	Major surgery (>45 minutes)	19	1	29	1	0.92	0.95	0.96	0.92	0.95	0.97	0.95	0.97
	Laparoscopic surgery (>45 minutes)	0	0	46	4	0.84	NA	0.92	0.00	0.00	1.00	NA	0.92
	Malignancy (present or previous)	18	2	30	0	0.92	0.95	0.96	0.92	1.00	0.94	0.90	1.00
	Patient confined to bed (>72 hours)	48	1	1	0	0.96	0.99	0.98	0.66	1.00	0.50	0.98	1.00
	Immobilizing plaster cast (<1 month)	5	0	45	0	1.00	1.00	1.00	1.00	1.00	1.00	1.00	1.00
	central venous access	37	3	10	0	0.88	0.96	0.94	0.83	1.00	0.77	0.92	1.00
	History of DVT/PE	0	0	50	0	1.00	NA	1.00	NA	NA	1.00	NA	1.00
	Family history of thrombosis	0	0	50	0	1.00	NA	1.00	NA	NA	1.00	NA	1.00
	Positive Factor V Leiden	0	0	50	0	1.00	NA	1.00	NA	NA	1.00	NA	1.00
	Positive Prothrombin 20210A	0	0	50	0	1.00	NA	1.00	NA	NA	1.00	NA	1.00
	Positive lupus anticoagulant	0	0	50	0	1.00	NA	1.00	NA	NA	1.00	NA	1.00
	Elevated anticardiolipin antibodies	0	0	50	0	1.00	NA	1.00	NA	NA	1.00	NA	1.00
	Elevated serum homocysteine	4	0	46	0	1.00	1.00	1.00	1.00	1.00	1.00	1.00	1.00
	Heparin-induced thrombocytopenia (HIT)	0	0	50	0	1.00	NA	1.00	NA	NA	1.00	NA	1.00
	Other congenital or acquired thrombophilia	0	1	49	0	0.96	NA	0.98	0.00	NA	0.98	0.00	1.00
	Stroke (<1 month)	10	1	36	3	0.84	0.83	0.92	0.78	0.77	0.97	0.91	0.92
	Elective major lower extremity arthroplasty	0	0	50	0	1.00	NA	1.00	NA	NA	1.00	NA	1.00

prompt	Items	TP	FP	TN	FN	PABAK	F1	Accuracy	Kappa	Sensitivity	Specificity	PPV	NPV
	Hip, pelvis or leg fracture (<1 month)	9	1	39	1	0.92	0.90	0.96	0.87	0.90	0.97	0.90	0.97
	Acute spinal cord injury (paralysis) (<1 month)	0	0	50	0	1.00	NA	1.00	NA	NA	1.00	NA	1.00
	Multiple trauma (<1 month)	10	1	39	0	0.96	0.95	0.98	0.94	1.00	0.97	0.91	1.00

Abbreviations: TP, true positive; FP, false positive; TN, true negative; FN, false negative; Kappa, Cohen's Kappa coefficient; PABAK, Prevalence-Adjusted Bias-Adjusted Kappa; PPV, positive predictive value; NPV, negative predictive value; F1, F1-score; DVT, deep vein thrombosis; PE, pulmonary embolism; BMI, body mass index.

Supplementary Table S16. Performance of Deepseek-V3 with Different Prompting Strategies for Item-Level Caprini Score Extraction in the Development Dataset

prompt	Items	TP	FP	TN	FN	PABAK	F1	Accuracy	Kappa	Sensitivity	Specificity	PPV	NPV
Basic prompt	Age (41-60 years)	11	1	38	0	0.96	0.96	0.98	0.94	1.00	0.97	0.92	1.00
	Age (60-74 years)	22	0	27	1	0.96	0.98	0.98	0.96	0.96	1.00	1.00	0.96
	Age (≥75 years)	6	0	44	0	1.00	1.00	1.00	1.00	1.00	1.00	1.00	1.00
	Minor surgery planned	2	1	38	9	0.60	0.29	0.80	0.21	0.18	0.97	0.67	0.81
	Obesity (BMI>25)	12	0	37	1	0.96	0.96	0.98	0.95	0.92	1.00	1.00	0.97
	Swollen legs (current)	8	3	31	8	0.56	0.59	0.78	0.45	0.50	0.91	0.73	0.79
	Varicose veins	0	0	50	0	1.00	NA	1.00	NA	NA	1.00	NA	1.00
	Pregnancy or postpartum (<1 month)	0	0	50	0	1.00	NA	1.00	NA	NA	1.00	NA	1.00
	History of unexplained stillborn infant, recurrent spontaneous abortion (≥3), premature birth with toxemia or growth-restricted infant	0	0	50	0	1.00	NA	1.00	NA	NA	1.00	NA	1.00
	Oral contraceptives or hormone replacement therapy	0	0	50	0	1.00	NA	1.00	NA	NA	1.00	NA	1.00
	Sepsis (<1 month)	6	3	33	8	0.56	0.52	0.78	0.39	0.43	0.92	0.67	0.80
	Serious lung disease incl.pneumonia (<1 month)	17	1	17	15	0.36	0.68	0.68	0.41	0.53	0.94	0.94	0.53
	Abnormal pulmonary function	1	0	23	26	-0.04	0.07	0.48	0.03	0.04	1.00	1.00	0.47
	Acute myocardial infarction	1	2	46	1	0.88	0.40	0.94	0.37	0.50	0.96	0.33	0.98
	Congestive heart failure (<1 month)	12	2	35	1	0.88	0.89	0.94	0.85	0.92	0.95	0.86	0.97
	History of inflammatory bowel disease	0	0	50	0	1.00	NA	1.00	NA	NA	1.00	NA	1.00
	Medical patient currently at bed rest	0	0	50	0	1.00	NA	1.00	NA	NA	1.00	NA	1.00
	Arthroscopic surgery	0	0	50	0	1.00	NA	1.00	NA	NA	1.00	NA	1.00
	Major surgery (>45 minutes)	17	3	27	3	0.76	0.85	0.88	0.75	0.85	0.90	0.85	0.90

prompt	Items	TP	FP	TN	FN	PABAK	F1	Accuracy	Kappa	Sensitivity	Specificity	PPV	NPV
	Laparoscopic surgery (>45 minutes)	1	0	46	3	0.88	0.40	0.94	0.38	0.25	1.00	1.00	0.94
	Malignancy (present or previous)	18	3	29	0	0.88	0.92	0.94	0.87	1.00	0.91	0.86	1.00
	Patient confined to bed (>72 hours)	35	0	2	13	0.48	0.84	0.74	0.18	0.73	1.00	1.00	0.13
	Immobilizing plaster cast (<1 month)	2	1	44	3	0.84	0.50	0.92	0.46	0.40	0.98	0.67	0.94
	central venous access	34	4	9	3	0.72	0.91	0.86	0.63	0.92	0.69	0.89	0.75
	History of DVT/PE	0	5	45	0	0.80	NA	0.90	0.00	NA	0.90	0.00	1.00
	Family history of thrombosis	0	0	50	0	1.00	NA	1.00	NA	NA	1.00	NA	1.00
	Positive Factor V Leiden	0	0	50	0	1.00	NA	1.00	NA	NA	1.00	NA	1.00
	Positive Prothrombin 20210A	0	0	50	0	1.00	NA	1.00	NA	NA	1.00	NA	1.00
	Positive lupus anticoagulant	0	0	50	0	1.00	NA	1.00	NA	NA	1.00	NA	1.00
	Elevated anticardiolipin antibodies	0	0	50	0	1.00	NA	1.00	NA	NA	1.00	NA	1.00
	Elevated serum homocysteine	0	0	46	4	0.84	NA	0.92	0.00	0.00	1.00	NA	0.92
	Heparin-induced thrombocytopenia (HIT)	0	0	50	0	1.00	NA	1.00	NA	NA	1.00	NA	1.00
	Other congenital or acquired thrombophilia	0	0	50	0	1.00	NA	1.00	NA	NA	1.00	NA	1.00
	Stroke (<1 month)	3	0	37	10	0.60	0.38	0.80	0.31	0.23	1.00	1.00	0.79
	Elective major lower extremity arthroplasty	0	0	50	0	1.00	NA	1.00	NA	NA	1.00	NA	1.00
	Hip, pelvis or leg fracture (<1 month)	5	0	40	5	0.80	0.67	0.90	0.62	0.50	1.00	1.00	0.89
	Acute spinal cord injury (paralysis) (<1 month)	0	0	50	0	1.00	NA	1.00	NA	NA	1.00	NA	1.00
	Multiple trauma (<1 month)	6	0	40	4	0.84	0.75	0.92	0.71	0.60	1.00	1.00	0.91
	Age (41-60 years)	11	1	38	0	0.96	0.96	0.98	0.94	1.00	0.97	0.92	1.00
	Age (60-74 years)	22	0	27	1	0.96	0.98	0.98	0.96	0.96	1.00	1.00	0.96
**Complex prompt**	Age (≥75 years)	6	0	44	0	1.00	1.00	1.00	1.00	1.00	1.00	1.00	1.00
	Minor surgery planned	0	0	39	11	0.56	NA	0.78	0.00	0.00	1.00	NA	0.78
	Obesity (BMI>25)	12	0	37	1	0.96	0.96	0.98	0.95	0.92	1.00	1.00	0.97
	Swollen legs (current)	11	1	33	5	0.76	0.79	0.88	0.70	0.69	0.97	0.92	0.87

prompt	Items	TP	FP	TN	FN	PABAK	F1	Accuracy	Kappa	Sensitivity	Specificity	PPV	NPV
	Varicose veins	0	0	50	0	1.00	NA	1.00	NA	NA	1.00	NA	1.00
	Pregnancy or postpartum (<1 month)	0	0	50	0	1.00	NA	1.00	NA	NA	1.00	NA	1.00
	History of unexplained stillborn infant, recurrent spontaneous abortion (≥3), premature birth with toxemia or growth-restricted infant	0	0	50	0	1.00	NA	1.00	NA	NA	1.00	NA	1.00
	Oral contraceptives or hormone replacement therapy	0	2	48	0	0.92	NA	0.96	0.00	NA	0.96	0.00	1.00
	Sepsis (<1 month)	10	5	31	4	0.64	0.69	0.82	0.56	0.71	0.86	0.67	0.89
	Serious lung disease incl.pneumonia (<1 month)	20	1	17	12	0.48	0.75	0.74	0.50	0.62	0.94	0.95	0.59
	Abnormal pulmonary function	9	0	23	18	0.28	0.50	0.64	0.32	0.33	1.00	1.00	0.56
	Acute myocardial infarction	0	1	47	2	0.88	NA	0.94	-0.03	0.00	0.98	0.00	0.96
	Congestive heart failure (<1 month)	12	1	36	1	0.92	0.92	0.96	0.90	0.92	0.97	0.92	0.97
	History of inflammatory bowel disease	0	0	50	0	1.00	NA	1.00	NA	NA	1.00	NA	1.00
	Medical patient currently at bed rest	0	0	50	0	1.00	NA	1.00	NA	NA	1.00	NA	1.00
	Arthroscopic surgery	0	0	50	0	1.00	NA	1.00	NA	NA	1.00	NA	1.00
	Major surgery (>45 minutes)	16	3	27	4	0.72	0.82	0.86	0.71	0.80	0.90	0.84	0.87
	Laparoscopic surgery (>45 minutes)	2	0	46	2	0.92	0.67	0.96	0.65	0.50	1.00	1.00	0.96
	Malignancy (present or previous)	18	0	32	0	1.00	1.00	1.00	1.00	1.00	1.00	1.00	1.00
	Patient confined to bed (>72 hours)	30	1	1	18	0.24	0.76	0.62	0.02	0.62	0.50	0.97	0.05
	Immobilizing plaster cast (<1 month)	3	0	45	2	0.92	0.75	0.96	0.73	0.60	1.00	1.00	0.96
	central venous access	33	4	9	4	0.68	0.89	0.84	0.58	0.89	0.69	0.89	0.69
	History of DVT/PE	0	9	41	0	0.64	NA	0.82	0.00	NA	0.82	0.00	1.00
	Family history of thrombosis	0	0	50	0	1.00	NA	1.00	NA	NA	1.00	NA	1.00
	Positive Factor V Leiden	0	0	50	0	1.00	NA	1.00	NA	NA	1.00	NA	1.00

prompt	Items	TP	FP	TN	FN	PABAK	F1	Accuracy	Kappa	Sensitivity	Specificity	PPV	NPV
	Positive Prothrombin 20210A	0	0	50	0	1.00	NA	1.00	NA	NA	1.00	NA	1.00
	Positive lupus anticoagulant	0	0	50	0	1.00	NA	1.00	NA	NA	1.00	NA	1.00
	Elevated anticardiolipin antibodies	0	0	50	0	1.00	NA	1.00	NA	NA	1.00	NA	1.00
	Elevated serum homocysteine	3	0	46	1	0.96	0.86	0.98	0.85	0.75	1.00	1.00	0.98
	Heparin-induced thrombocytopenia (HIT)	0	0	50	0	1.00	NA	1.00	NA	NA	1.00	NA	1.00
	Other congenital or acquired thrombophilia	0	0	50	0	1.00	NA	1.00	NA	NA	1.00	NA	1.00
	Stroke (<1 month)	6	0	37	7	0.72	0.63	0.86	0.56	0.46	1.00	1.00	0.84
	Elective major lower extremity arthroplasty	0	0	50	0	1.00	NA	1.00	NA	NA	1.00	NA	1.00
	Hip, pelvis or leg fracture (<1 month)	6	0	40	4	0.84	0.75	0.92	0.71	0.60	1.00	1.00	0.91
	Acute spinal cord injury (paralysis) (<1 month)	0	1	49	0	0.96	NA	0.98	0.00	NA	0.98	0.00	1.00
	Multiple trauma (<1 month)	5	0	40	5	0.80	0.67	0.90	0.62	0.50	1.00	1.00	0.89
	Age (41-60 years)	11	1	38	0	0.96	0.96	0.98	0.94	1.00	0.97	0.92	1.00
	Age (60-74 years)	22	0	27	1	0.96	0.98	0.98	0.96	0.96	1.00	1.00	0.96
	Age (≥75 years)	6	0	44	0	1.00	1.00	1.00	1.00	1.00	1.00	1.00	1.00
	Minor surgery planned	2	3	36	9	0.52	0.25	0.76	0.13	0.18	0.92	0.40	0.80
	Obesity (BMI>25)	12	0	37	1	0.96	0.96	0.98	0.95	0.92	1.00	1.00	0.97
	Swollen legs (current)	10	2	32	6	0.68	0.71	0.84	0.61	0.62	0.94	0.83	0.84
**Expert prompt**	Varicose veins	0	0	50	0	1.00	NA	1.00	NA	NA	1.00	NA	1.00
	Pregnancy or postpartum (<1 month)	0	0	50	0	1.00	NA	1.00	NA	NA	1.00	NA	1.00
	History of unexplained stillborn infant, recurrent spontaneous abortion (≥3), premature birth with toxemia or growth-restricted infant	0	0	50	0	1.00	NA	1.00	NA	NA	1.00	NA	1.00
	Oral contraceptives or hormone replacement therapy	0	3	47	0	0.88	NA	0.94	0.00	NA	0.94	0.00	1.00
	Sepsis (<1 month)	12	1	35	2	0.88	0.89	0.94	0.85	0.86	0.97	0.92	0.95

prompt	Items	TP	FP	TN	FN	PABAK	F1	Accuracy	Kappa	Sensitivity	Specificity	PPV	NPV
	Serious lung disease incl.pneumonia (<1 month)	17	0	18	15	0.40	0.69	0.70	0.45	0.53	1.00	1.00	0.55
	Abnormal pulmonary function	14	0	23	13	0.48	0.68	0.74	0.50	0.52	1.00	1.00	0.64
	Acute myocardial infarction	0	0	48	2	0.92	NA	0.96	0.00	0.00	1.00	NA	0.96
	Congestive heart failure (<1 month)	12	1	36	1	0.92	0.92	0.96	0.90	0.92	0.97	0.92	0.97
	History of inflammatory bowel disease	0	0	50	0	1.00	NA	1.00	NA	NA	1.00	NA	1.00
	Medical patient currently at bed rest	0	0	50	0	1.00	NA	1.00	NA	NA	1.00	NA	1.00
	Arthroscopic surgery	0	0	50	0	1.00	NA	1.00	NA	NA	1.00	NA	1.00
	Major surgery (>45 minutes)	16	2	28	4	0.76	0.84	0.88	0.75	0.80	0.93	0.89	0.88
	Laparoscopic surgery (>45 minutes)	2	0	46	2	0.92	0.67	0.96	0.65	0.50	1.00	1.00	0.96
	Malignancy (present or previous)	18	1	31	0	0.96	0.97	0.98	0.96	1.00	0.97	0.95	1.00
	Patient confined to bed (>72 hours)	36	0	2	12	0.52	0.86	0.76	0.19	0.75	1.00	1.00	0.14
	Immobilizing plaster cast (<1 month)	4	0	45	1	0.96	0.89	0.98	0.88	0.80	1.00	1.00	0.98
	central venous access	34	2	11	3	0.80	0.93	0.90	0.75	0.92	0.85	0.94	0.79
	History of DVT/PE	0	6	44	0	0.76	NA	0.88	0.00	NA	0.88	0.00	1.00
	Family history of thrombosis	0	0	50	0	1.00	NA	1.00	NA	NA	1.00	NA	1.00
	Positive Factor V Leiden	0	0	50	0	1.00	NA	1.00	NA	NA	1.00	NA	1.00
	Positive Prothrombin 20210A	0	0	50	0	1.00	NA	1.00	NA	NA	1.00	NA	1.00
	Positive lupus anticoagulant	0	0	50	0	1.00	NA	1.00	NA	NA	1.00	NA	1.00
	Elevated anticardiolipin antibodies	0	0	50	0	1.00	NA	1.00	NA	NA	1.00	NA	1.00
	Elevated serum homocysteine	2	0	46	2	0.92	0.67	0.96	0.65	0.50	1.00	1.00	0.96
	Heparin-induced thrombocytopenia (HIT)	0	0	50	0	1.00	NA	1.00	NA	NA	1.00	NA	1.00
	Other congenital or acquired thrombophilia	0	0	50	0	1.00	NA	1.00	NA	NA	1.00	NA	1.00
	Stroke (<1 month)	6	0	37	7	0.72	0.63	0.86	0.56	0.46	1.00	1.00	0.84
	Elective major lower extremity arthroplasty	0	0	50	0	1.00	NA	1.00	NA	NA	1.00	NA	1.00

prompt	Items	TP	FP	TN	FN	PABAK	F1	Accuracy	Kappa	Sensitivity	Specificity	PPV	NPV
	Hip, pelvis or leg fracture (<1 month)	6	0	40	4	0.84	0.75	0.92	0.71	0.60	1.00	1.00	0.91
	Acute spinal cord injury (paralysis) (<1 month)	0	0	50	0	1.00	NA	1.00	NA	NA	1.00	NA	1.00
	Multiple trauma (<1 month)	2	0	40	8	0.68	0.33	0.84	0.29	0.20	1.00	1.00	0.83

Abbreviations: TP, true positive; FP, false positive; TN, true negative; FN, false negative; Kappa, Cohen's Kappa coefficient; PABAK, Prevalence-Adjusted Bias-Adjusted Kappa; PPV, positive predictive value; NPV, negative predictive value; F1, F1-score; DVT, deep vein thrombosis; PE, pulmonary embolism; BMI, body mass index.

Supplementary Table S17. Performance of GLM-4.6 with Different Prompting Strategies for Item-Level Caprini Score Extraction in the Development Dataset

prompt	Items	TP	FP	TN	FN	PABAK	F1	Accuracy	Kappa	Sensitivity	Specificity	PPV	NPV
**Basic prompt**	Age (41-60 years)	11	1	38	0	0.96	0.96	0.98	0.94	1.00	0.97	0.92	1.00
	Age (60-74 years)	22	0	27	1	0.96	0.98	0.98	0.96	0.96	1.00	1.00	0.96
	Age (≥75 years)	6	0	44	0	1.00	1.00	1.00	1.00	1.00	1.00	1.00	1.00
	Minor surgery planned	1	4	35	10	0.44	0.12	0.72	-0.01	0.09	0.90	0.20	0.78
	Obesity (BMI>25)	12	0	37	1	0.96	0.96	0.98	0.95	0.92	1.00	1.00	0.97
	Swollen legs (current)	13	2	32	3	0.80	0.84	0.90	0.77	0.81	0.94	0.87	0.91
	Varicose veins	0	0	50	0	1.00	NA	1.00	NA	NA	1.00	NA	1.00
	Pregnancy or postpartum (<1 month)	0	0	50	0	1.00	NA	1.00	NA	NA	1.00	NA	1.00
	History of unexplained stillborn infant, recurrent spontaneous abortion (≥3), premature birth with toxemia or growth-restricted infant	0	0	50	0	1.00	NA	1.00	NA	NA	1.00	NA	1.00
	Oral contraceptives or hormone replacement therapy	0	4	46	0	0.84	NA	0.92	0.00	NA	0.92	0.00	1.00
	Sepsis (<1 month)	11	4	32	3	0.72	0.76	0.86	0.66	0.79	0.89	0.73	0.91
	Serious lung disease incl.pneumonia (<1 month)	23	2	16	9	0.56	0.81	0.78	0.56	0.72	0.89	0.92	0.64
	Abnormal pulmonary function	9	1	22	18	0.24	0.49	0.62	0.27	0.33	0.96	0.90	0.55
	Acute myocardial infarction	0	1	47	2	0.88	NA	0.94	-0.03	0.00	0.98	0.00	0.96
	Congestive heart failure (<1 month)	11	2	35	2	0.84	0.85	0.92	0.79	0.85	0.95	0.85	0.95
	History of inflammatory bowel disease	0	0	50	0	1.00	NA	1.00	NA	NA	1.00	NA	1.00
	Medical patient currently at bed rest	0	3	47	0	0.88	NA	0.94	0.00	NA	0.94	0.00	1.00
	Arthroscopic surgery	0	0	50	0	1.00	NA	1.00	NA	NA	1.00	NA	1.00
	Major surgery (>45 minutes)	15	3	27	5	0.68	0.79	0.84	0.66	0.75	0.90	0.83	0.84

prompt	Items	TP	FP	TN	FN	PABAK	F1	Accuracy	Kappa	Sensitivity	Specificity	PPV	NPV
	Laparoscopic surgery (>45 minutes)	0	1	45	4	0.80	NA	0.90	-0.03	0.00	0.98	0.00	0.92
	Malignancy (present or previous)	18	2	30	0	0.92	0.95	0.96	0.92	1.00	0.94	0.90	1.00
	Patient confined to bed (>72 hours)	33	0	2	15	0.40	0.81	0.70	0.15	0.69	1.00	1.00	0.12
	Immobilizing plaster cast (<1 month)	5	0	45	0	1.00	1.00	1.00	1.00	1.00	1.00	1.00	1.00
	central venous access	37	5	8	0	0.80	0.94	0.90	0.70	1.00	0.62	0.88	1.00
	History of DVT/PE	0	6	44	0	0.76	NA	0.88	0.00	NA	0.88	0.00	1.00
	Family history of thrombosis	0	0	50	0	1.00	NA	1.00	NA	NA	1.00	NA	1.00
	Positive Factor V Leiden	0	0	50	0	1.00	NA	1.00	NA	NA	1.00	NA	1.00
	Positive Prothrombin 20210A	0	0	50	0	1.00	NA	1.00	NA	NA	1.00	NA	1.00
	Positive lupus anticoagulant	0	0	50	0	1.00	NA	1.00	NA	NA	1.00	NA	1.00
	Elevated anticardiolipin antibodies	0	0	50	0	1.00	NA	1.00	NA	NA	1.00	NA	1.00
	Elevated serum homocysteine	4	0	46	0	1.00	1.00	1.00	1.00	1.00	1.00	1.00	1.00
	Heparin-induced thrombocytopenia (HIT)	0	0	50	0	1.00	NA	1.00	NA	NA	1.00	NA	1.00
	Other congenital or acquired thrombophilia	0	0	50	0	1.00	NA	1.00	NA	NA	1.00	NA	1.00
	Stroke (<1 month)	10	5	32	3	0.68	0.71	0.84	0.60	0.77	0.86	0.67	0.91
	Elective major lower extremity arthroplasty	0	0	50	0	1.00	NA	1.00	NA	NA	1.00	NA	1.00
	Hip, pelvis or leg fracture (<1 month)	8	1	39	2	0.88	0.84	0.94	0.81	0.80	0.97	0.89	0.95
	Acute spinal cord injury (paralysis) (<1 month)	0	0	50	0	1.00	NA	1.00	NA	NA	1.00	NA	1.00
	Multiple trauma (<1 month)	7	0	40	3	0.88	0.82	0.94	0.79	0.70	1.00	1.00	0.93
	Age (41-60 years)	11	1	38	0	0.96	0.96	0.98	0.94	1.00	0.97	0.92	1.00
	Age (60-74 years)	22	0	27	1	0.96	0.98	0.98	0.96	0.96	1.00	1.00	0.96
**Complex prompt**	Age (≥75 years)	6	0	44	0	1.00	1.00	1.00	1.00	1.00	1.00	1.00	1.00
	Minor surgery planned	0	3	36	11	0.44	NA	0.72	-0.10	0.00	0.92	0.00	0.77
	Obesity (BMI>25)	12	0	37	1	0.96	0.96	0.98	0.95	0.92	1.00	1.00	0.97
	Swollen legs (current)	15	2	32	1	0.88	0.91	0.94	0.86	0.94	0.94	0.88	0.97

prompt	Items	TP	FP	TN	FN	PABAK	F1	Accuracy	Kappa	Sensitivity	Specificity	PPV	NPV
	Varicose veins	0	0	50	0	1.00	NA	1.00	NA	NA	1.00	NA	1.00
	Pregnancy or postpartum (<1 month)	0	0	50	0	1.00	NA	1.00	NA	NA	1.00	NA	1.00
	History of unexplained stillborn infant, recurrent spontaneous abortion (≥3), premature birth with toxemia or growth-restricted infant	0	0	50	0	1.00	NA	1.00	NA	NA	1.00	NA	1.00
	Oral contraceptives or hormone replacement therapy	0	4	46	0	0.84	NA	0.92	0.00	NA	0.92	0.00	1.00
	Sepsis (<1 month)	10	7	29	4	0.56	0.65	0.78	0.49	0.71	0.81	0.59	0.88
	Serious lung disease incl.pneumonia (<1 month)	26	1	17	6	0.72	0.88	0.86	0.71	0.81	0.94	0.96	0.74
	Abnormal pulmonary function	18	0	23	9	0.64	0.80	0.82	0.65	0.67	1.00	1.00	0.72
	Acute myocardial infarction	0	0	48	2	0.92	NA	0.96	0.00	0.00	1.00	NA	0.96
	Congestive heart failure (<1 month)	12	1	36	1	0.92	0.92	0.96	0.90	0.92	0.97	0.92	0.97
	History of inflammatory bowel disease	0	0	50	0	1.00	NA	1.00	NA	NA	1.00	NA	1.00
	Medical patient currently at bed rest	0	1	49	0	0.96	NA	0.98	0.00	NA	0.98	0.00	1.00
	Arthroscopic surgery	0	0	50	0	1.00	NA	1.00	NA	NA	1.00	NA	1.00
	Major surgery (>45 minutes)	17	5	25	3	0.68	0.81	0.84	0.67	0.85	0.83	0.77	0.89
	Laparoscopic surgery (>45 minutes)	0	0	46	4	0.84	NA	0.92	0.00	0.00	1.00	NA	0.92
	Malignancy (present or previous)	18	1	31	0	0.96	0.97	0.98	0.96	1.00	0.97	0.95	1.00
	Patient confined to bed (>72 hours)	28	0	2	20	0.20	0.74	0.60	0.10	0.58	1.00	1.00	0.09
	Immobilizing plaster cast (<1 month)	4	0	45	1	0.96	0.89	0.98	0.88	0.80	1.00	1.00	0.98
	central venous access	35	3	10	2	0.80	0.93	0.90	0.73	0.95	0.77	0.92	0.83
	History of DVT/PE	0	5	45	0	0.80	NA	0.90	0.00	NA	0.90	0.00	1.00
	Family history of thrombosis	0	0	50	0	1.00	NA	1.00	NA	NA	1.00	NA	1.00
	Positive Factor V Leiden	0	0	50	0	1.00	NA	1.00	NA	NA	1.00	NA	1.00

prompt	Items	TP	FP	TN	FN	PABAK	F1	Accuracy	Kappa	Sensitivity	Specificity	PPV	NPV
	Positive Prothrombin 20210A	0	0	50	0	1.00	NA	1.00	NA	NA	1.00	NA	1.00
	Positive lupus anticoagulant	0	0	50	0	1.00	NA	1.00	NA	NA	1.00	NA	1.00
	Elevated anticardiolipin antibodies	0	0	50	0	1.00	NA	1.00	NA	NA	1.00	NA	1.00
	Elevated serum homocysteine	4	0	46	0	1.00	1.00	1.00	1.00	1.00	1.00	1.00	1.00
	Heparin-induced thrombocytopenia (HIT)	0	0	50	0	1.00	NA	1.00	NA	NA	1.00	NA	1.00
	Other congenital or acquired thrombophilia	0	0	50	0	1.00	NA	1.00	NA	NA	1.00	NA	1.00
	Stroke (<1 month)	9	2	35	4	0.76	0.75	0.88	0.67	0.69	0.95	0.82	0.90
	Elective major lower extremity arthroplasty	0	0	50	0	1.00	NA	1.00	NA	NA	1.00	NA	1.00
	Hip, pelvis or leg fracture (<1 month)	8	1	39	2	0.88	0.84	0.94	0.81	0.80	0.97	0.89	0.95
	Acute spinal cord injury (paralysis) (<1 month)	0	0	50	0	1.00	NA	1.00	NA	NA	1.00	NA	1.00
	Multiple trauma (<1 month)	8	0	40	2	0.92	0.89	0.96	0.86	0.80	1.00	1.00	0.95
**Expert prompt**	Age (41-60 years)	11	1	33	0	0.96	0.96	0.98	0.94	1.00	0.97	0.92	1.00
	Age (60-74 years)	19	0	25	1	0.96	0.97	0.98	0.95	0.95	1.00	1.00	0.96
	Age (≥75 years)	4	0	41	0	1.00	1.00	1.00	1.00	1.00	1.00	1.00	1.00
	Minor surgery planned	2	3	34	6	0.60	0.31	0.80	0.20	0.25	0.92	0.40	0.85
	Obesity (BMI>25)	11	0	33	1	0.96	0.96	0.98	0.94	0.92	1.00	1.00	0.97
	Swollen legs (current)	11	1	30	3	0.82	0.85	0.91	0.78	0.79	0.97	0.92	0.91
	Varicose veins	0	0	45	0	1.00	NA	1.00	NA	NA	1.00	NA	1.00
	Pregnancy or postpartum (<1 month)	0	0	45	0	1.00	NA	1.00	NA	NA	1.00	NA	1.00
	History of unexplained stillborn infant, recurrent spontaneous abortion (≥3), premature birth with toxemia or growth-restricted infant	0	0	45	0	1.00	NA	1.00	NA	NA	1.00	NA	1.00
	Oral contraceptives or hormone replacement therapy	0	1	44	0	0.96	NA	0.98	0.00	NA	0.98	0.00	1.00
	Sepsis (<1 month)	12	0	32	1	0.96	0.96	0.98	0.94	0.92	1.00	1.00	0.97

prompt	Items	TP	FP	TN	FN	PABAK	F1	Accuracy	Kappa	Sensitivity	Specificity	PPV	NPV
	Serious lung disease incl.pneumonia (<1 month)	13	0	18	14	0.38	0.65	0.69	0.43	0.48	1.00	1.00	0.56
	Abnormal pulmonary function	17	0	20	8	0.64	0.81	0.82	0.65	0.68	1.00	1.00	0.71
	Acute myocardial infarction	0	0	43	2	0.91	NA	0.96	0.00	0.00	1.00	NA	0.96
	Congestive heart failure (<1 month)	9	0	35	1	0.96	0.95	0.98	0.93	0.90	1.00	1.00	0.97
	History of inflammatory bowel disease	0	0	45	0	1.00	NA	1.00	NA	NA	1.00	NA	1.00
	Medical patient currently at bed rest	0	0	45	0	1.00	NA	1.00	NA	NA	1.00	NA	1.00
	Arthroscopic surgery	0	0	45	0	1.00	NA	1.00	NA	NA	1.00	NA	1.00
	Major surgery (>45 minutes)	17	1	24	3	0.82	0.89	0.91	0.82	0.85	0.96	0.94	0.89
	Laparoscopic surgery (>45 minutes)	2	0	41	2	0.91	0.67	0.96	0.65	0.50	1.00	1.00	0.95
	Malignancy (present or previous)	18	2	25	0	0.91	0.95	0.96	0.91	1.00	0.93	0.90	1.00
	Patient confined to bed (>72 hours)	32	0	2	11	0.51	0.85	0.76	0.21	0.74	1.00	1.00	0.15
	Immobilizing plaster cast (<1 month)	5	0	40	0	1.00	1.00	1.00	1.00	1.00	1.00	1.00	1.00
	central venous access	32	3	8	2	0.78	0.93	0.89	0.69	0.94	0.73	0.91	0.80
	History of DVT/PE	0	1	44	0	0.96	NA	0.98	0.00	NA	0.98	0.00	1.00
	Family history of thrombosis	0	0	45	0	1.00	NA	1.00	NA	NA	1.00	NA	1.00
	Positive Factor V Leiden	0	0	45	0	1.00	NA	1.00	NA	NA	1.00	NA	1.00
	Positive Prothrombin 20210A	0	0	45	0	1.00	NA	1.00	NA	NA	1.00	NA	1.00
	Positive lupus anticoagulant	0	0	45	0	1.00	NA	1.00	NA	NA	1.00	NA	1.00
	Elevated anticardiolipin antibodies	0	0	45	0	1.00	NA	1.00	NA	NA	1.00	NA	1.00
	Elevated serum homocysteine	3	0	42	0	1.00	1.00	1.00	1.00	1.00	1.00	1.00	1.00
	Heparin-induced thrombocytopenia (HIT)	0	0	45	0	1.00	NA	1.00	NA	NA	1.00	NA	1.00
	Other congenital or acquired thrombophilia	0	0	45	0	1.00	NA	1.00	NA	NA	1.00	NA	1.00
	Stroke (<1 month)	3	0	35	7	0.69	0.46	0.84	0.40	0.30	1.00	1.00	0.83
	Elective major lower extremity arthroplasty	0	0	45	0	1.00	NA	1.00	NA	NA	1.00	NA	1.00

prompt	Items	TP	FP	TN	FN	PABAK	F1	Accuracy	Kappa	Sensitivity	Specificity	PPV	NPV
	Hip, pelvis or leg fracture (<1 month)	7	0	37	1	0.96	0.93	0.98	0.92	0.88	1.00	1.00	0.97
	Acute spinal cord injury (paralysis) (<1 month)	0	0	45	0	1.00	NA	1.00	NA	NA	1.00	NA	1.00
	Multiple trauma (<1 month)	7	0	35	3	0.87	0.82	0.93	0.78	0.70	1.00	1.00	0.92

Abbreviations: TP, true positive; FP, false positive; TN, true negative; FN, false negative; Kappa, Cohen's Kappa coefficient; PABAK, Prevalence-Adjusted Bias-Adjusted Kappa; PPV, positive predictive value; NPV, negative predictive value; F1, F1-score; DVT, deep vein thrombosis; PE, pulmonary embolism; BMI, body mass index.

Supplementary Table S18. Performance of Qwen3-Next with Basic and Complex Prompting Strategies for Item-Level Padua Score Extraction in the Test Dataset

prompt	Items	TP	FP	TN	FN	Accuracy	Kappa	PABAK	Sensitivity	Specificity	PPV	NPV	F1
Basic prompt	Active cancer	44	2	154	0	0.99	0.97	0.98	1.00	0.99	0.96	1.00	0.98
	Previous VTE	0	5	195	0	0.97	0.00	0.95	NA	0.97	0.00	1.00	NA
	Elderly age (≥70 years)	62	4	133	1	0.97	0.94	0.95	0.98	0.97	0.94	0.99	0.96
	Heart and/or respiratory failure	36	5	159	0	0.97	0.92	0.95	1.00	0.97	0.88	1.00	0.94
	Acute infection and/or rheumatologic disorder	69	11	118	2	0.94	0.86	0.87	0.97	0.91	0.86	0.98	0.91
	Obesity (BMI ≥30)	11	0	189	0	1.00	1.00	1.00	1.00	1.00	1.00	1.00	1.00
	Ongoing hormonal treatment	1	0	198	1	0.99	0.66	0.99	0.50	1.00	1.00	0.99	0.67
	Recent (≤1 month) trauma and/or surgery	11	113	75	1	0.43	0.06	-0.14	0.92	0.40	0.09	0.99	0.16
	Reduced mobility	58	16	96	30	0.77	0.53	0.54	0.66	0.86	0.78	0.76	0.72
	Acute myocardial infarction or ischemic stroke	13	4	182	1	0.97	0.83	0.95	0.93	0.98	0.76	0.99	0.84
	Already known thrombophilic condition	0	1	199	0	0.99	0.00	0.99	NA	0.99	0.00	1.00	NA
Complex prompt	Active cancer	41	0	156	3	0.98	0.96	0.97	0.93	1.00	1.00	0.98	0.96
	Previous VTE	0	3	197	0	0.98	0.00	0.97	NA	0.98	0.00	1.00	NA
	Elderly age (≥70 years)	61	3	134	2	0.97	0.94	0.95	0.97	0.98	0.95	0.99	0.96

prompt	Items	TP	FP	TN	FN	Accuracy	Kappa	PABAK	Sensitivity	Specificity	PPV	NPV	F1
	Heart and/or respiratory failure	35	8	156	1	0.95	0.86	0.91	0.97	0.95	0.81	0.99	0.89
	Acute infection and/or rheumatologic disorder	67	11	118	4	0.92	0.84	0.85	0.94	0.91	0.86	0.97	0.90
	Obesity (BMI ≥30)	11	0	189	0	1.00	1.00	1.00	1.00	1.00	1.00	1.00	1.00
	Ongoing hormonal treatment	1	0	198	1	0.99	0.66	0.99	0.50	1.00	1.00	0.99	0.67
	Recent (≤1 month) trauma and/or surgery	11	99	89	1	0.50	0.08	0.00	0.92	0.47	0.10	0.99	0.18
	Reduced mobility	43	4	108	45	0.76	0.48	0.51	0.49	0.96	0.91	0.71	0.64
	Acute myocardial infarction or ischemic stroke	13	4	182	1	0.97	0.83	0.95	0.93	0.98	0.76	0.99	0.84
	Already known thrombophilic condition	0	1	199	0	0.99	0.00	0.99	NA	0.99	0.00	1.00	NA

Abbreviations: TP, true positive; FP, false positive; TN, true negative; FN, false negative; Kappa, Cohen's Kappa coefficient; PABAK, Prevalence-Adjusted Bias-Adjusted Kappa; PPV, positive predictive value; NPV, negative predictive value; F1, F1-score; VTE, venous thromboembolism; BMI, body mass index.

Supplementary Table S19. Performance of Qwen3-Next with Basic and Complex Prompting Strategies for Item-Level Caprini Score Extraction in the Test Dataset

prompt	Items	TP	FP	TN	FN	PABAK	F1	Accuracy	Kappa	Sensitivity	Specificity	PPV	NPV
Basic prompt	Age (41-60 years)	40	8	150	2	0.90	0.89	0.95	0.86	0.95	0.95	0.83	0.99
	Age (60-74 years)	63	7	121	9	0.84	0.89	0.92	0.83	0.88	0.95	0.90	0.93
	Age (≥75 years)	39	2	155	4	0.94	0.93	0.97	0.91	0.91	0.99	0.95	0.97
	Minor surgery planned	19	19	149	13	0.68	0.54	0.84	0.45	0.59	0.89	0.50	0.92
	Obesity (BMI>25)	44	1	155	0	0.99	0.99	0.99	0.99	1.00	0.99	0.98	1.00
	Swollen legs (current)	14	3	172	11	0.86	0.67	0.93	0.63	0.56	0.98	0.82	0.94
	Varicose veins	6	0	194	0	1.00	1.00	1.00	1.00	1.00	1.00	1.00	1.00
	Pregnancy or postpartum (<1 month)	4	0	196	0	1.00	1.00	1.00	1.00	1.00	1.00	1.00	1.00
	History of unexplained stillborn infant, recurrent spontaneous abortion (≥3), premature birth with toxemia or growth-restricted infant	0	0	200	0	1.00	NA	1.00	NA	NA	1.00	NA	1.00
	Oral contraceptives or hormone replacement therapy	1	1	197	1	0.98	0.50	0.99	0.49	0.50	0.99	0.50	0.99
	Sepsis (<1 month)	7	15	177	1	0.84	0.47	0.92	0.43	0.88	0.92	0.32	0.99
	Serious lung disease incl.pneumonia (<1 month)	32	2	135	31	0.67	0.66	0.83	0.56	0.51	0.99	0.94	0.81
	Abnormal pulmonary function	16	7	160	17	0.76	0.57	0.88	0.50	0.48	0.96	0.70	0.90
	Acute myocardial infarction	3	0	197	0	1.00	1.00	1.00	1.00	1.00	1.00	1.00	1.00
	Congestive heart failure (<1 month)	18	2	177	3	0.95	0.88	0.97	0.86	0.86	0.99	0.90	0.98
	History of inflammatory bowel disease	0	0	200	0	1.00	NA	1.00	NA	NA	1.00	NA	1.00
	Medical patient currently at bed rest	4	4	160	32	0.64	0.18	0.82	0.12	0.11	0.98	0.50	0.83
	Arthroscopic surgery	2	1	197	0	0.99	0.80	0.99	0.80	1.00	0.99	0.67	1.00
	Major surgery (>45 minutes)	31	5	139	25	0.70	0.67	0.85	0.58	0.55	0.97	0.86	0.85

prompt	Items	TP	FP	TN	FN	PABAK	F1	Accuracy	Kappa	Sensitivity	Specificity	PPV	NPV
	Laparoscopic surgery (>45 minutes)	13	14	171	2	0.84	0.62	0.92	0.58	0.87	0.92	0.48	0.99
	Malignancy (present or previous)	47	3	146	4	0.93	0.93	0.96	0.91	0.92	0.98	0.94	0.97
	Patient confined to bed (>72 hours)	52	16	96	36	0.48	0.67	0.74	0.46	0.59	0.86	0.76	0.73
	Immobilizing plaster cast (<1 month)	2	0	195	3	0.97	0.57	0.98	0.57	0.40	1.00	1.00	0.98
	central venous access	23	12	160	5	0.83	0.73	0.92	0.68	0.82	0.93	0.66	0.97
	History of DVT/PE	0	4	196	0	0.96	NA	0.98	0.00	NA	0.98	0.00	1.00
	Family history of thrombosis	0	0	200	0	1.00	NA	1.00	NA	NA	1.00	NA	1.00
	Positive Factor V Leiden	0	0	200	0	1.00	NA	1.00	NA	NA	1.00	NA	1.00
	Positive Prothrombin 20210A	0	0	200	0	1.00	NA	1.00	NA	NA	1.00	NA	1.00
	Positive lupus anticoagulant	0	0	200	0	1.00	NA	1.00	NA	NA	1.00	NA	1.00
	Elevated anticardiolipin antibodies	1	0	199	0	1.00	1.00	1.00	1.00	1.00	1.00	1.00	1.00
	Elevated serum homocysteine	5	0	195	0	1.00	1.00	1.00	1.00	1.00	1.00	1.00	1.00
	Heparin-induced thrombocytopenia (HIT)	0	0	200	0	1.00	NA	1.00	NA	NA	1.00	NA	1.00
	Other congenital or acquired thrombophilia	0	0	199	1	0.99	NA	0.99	0.00	0.00	1.00	NA	0.99
	Stroke (<1 month)	14	17	164	5	0.78	0.56	0.89	0.50	0.74	0.91	0.45	0.97
	Elective major lower extremity arthroplasty	4	1	195	0	0.99	0.89	0.99	0.89	1.00	0.99	0.80	1.00
	Hip, pelvis or leg fracture (<1 month)	11	2	184	3	0.95	0.81	0.97	0.80	0.79	0.99	0.85	0.98
	Acute spinal cord injury (paralysis) (<1 month)	0	0	200	0	1.00	NA	1.00	NA	NA	1.00	NA	1.00
	Multiple trauma (<1 month)	3	3	190	4	0.93	0.46	0.96	0.44	0.43	0.98	0.50	0.98
	Age (41-60 years)	41	9	149	1	0.90	0.89	0.95	0.86	0.98	0.94	0.82	0.99
	Age (60-74 years)	64	10	118	8	0.82	0.88	0.91	0.81	0.89	0.92	0.86	0.94
**Complex prompt**	Age (≥75 years)	40	1	156	3	0.96	0.95	0.98	0.94	0.93	0.99	0.98	0.98
	Minor surgery planned	15	16	152	17	0.67	0.48	0.83	0.38	0.47	0.90	0.48	0.90
	Obesity (BMI>25)	44	1	155	0	0.99	0.99	0.99	0.99	1.00	0.99	0.98	1.00
	Swollen legs (current)	21	1	174	4	0.95	0.89	0.97	0.88	0.84	0.99	0.95	0.98

prompt	Items	TP	FP	TN	FN	PABAK	F1	Accuracy	Kappa	Sensitivity	Specificity	PPV	NPV
	Varicose veins	6	0	194	0	1.00	1.00	1.00	1.00	1.00	1.00	1.00	1.00
	Pregnancy or postpartum (<1 month)	4	0	196	0	1.00	1.00	1.00	1.00	1.00	1.00	1.00	1.00
	History of unexplained stillborn infant, recurrent spontaneous abortion (≥3), premature birth with toxemia or growth-restricted infant	0	0	200	0	1.00	NA	1.00	NA	NA	1.00	NA	1.00
	Oral contraceptives or hormone replacement therapy	1	2	196	1	0.97	0.40	0.98	0.39	0.50	0.99	0.33	0.99
	Sepsis (<1 month)	4	5	187	4	0.91	0.47	0.95	0.45	0.50	0.97	0.44	0.98
	Serious lung disease incl.pneumonia (<1 month)	56	3	134	7	0.90	0.92	0.95	0.88	0.89	0.98	0.95	0.95
	Abnormal pulmonary function	18	6	161	15	0.79	0.63	0.90	0.57	0.55	0.96	0.75	0.91
	Acute myocardial infarction	3	0	197	0	1.00	1.00	1.00	1.00	1.00	1.00	1.00	1.00
	Congestive heart failure (<1 month)	17	2	177	4	0.94	0.85	0.97	0.83	0.81	0.99	0.89	0.98
	History of inflammatory bowel disease	0	0	200	0	1.00	NA	1.00	NA	NA	1.00	NA	1.00
	Medical patient currently at bed rest	13	10	154	23	0.67	0.44	0.83	0.35	0.36	0.94	0.57	0.87
	Arthroscopic surgery	2	1	197	0	0.99	0.80	0.99	0.80	1.00	0.99	0.67	1.00
	Major surgery (>45 minutes)	38	5	139	18	0.77	0.77	0.88	0.69	0.68	0.97	0.88	0.89
	Laparoscopic surgery (>45 minutes)	13	6	179	2	0.92	0.76	0.96	0.74	0.87	0.97	0.68	0.99
	Malignancy (present or previous)	48	2	147	3	0.95	0.95	0.97	0.93	0.94	0.99	0.96	0.98
	Patient confined to bed (>72 hours)	62	24	88	26	0.50	0.71	0.75	0.49	0.70	0.79	0.72	0.77
	Immobilizing plaster cast (<1 month)	2	0	195	3	0.97	0.57	0.98	0.57	0.40	1.00	1.00	0.98
	central venous access	22	6	166	6	0.88	0.79	0.94	0.75	0.79	0.97	0.79	0.97
	History of DVT/PE	0	6	194	0	0.94	NA	0.97	0.00	NA	0.97	0.00	1.00
	Family history of thrombosis	0	0	200	0	1.00	NA	1.00	NA	NA	1.00	NA	1.00
	Positive Factor V Leiden	0	0	200	0	1.00	NA	1.00	NA	NA	1.00	NA	1.00

prompt	Items	TP	FP	TN	FN	PABAK	F1	Accuracy	Kappa	Sensitivity	Specificity	PPV	NPV
	Positive Prothrombin 20210A	0	0	200	0	1.00	NA	1.00	NA	NA	1.00	NA	1.00
	Positive lupus anticoagulant	0	0	200	0	1.00	NA	1.00	NA	NA	1.00	NA	1.00
	Elevated anticardiolipin antibodies	1	0	199	0	1.00	1.00	1.00	1.00	1.00	1.00	1.00	1.00
	Elevated serum homocysteine	5	0	195	0	1.00	1.00	1.00	1.00	1.00	1.00	1.00	1.00
	Heparin-induced thrombocytopenia (HIT)	0	0	200	0	1.00	NA	1.00	NA	NA	1.00	NA	1.00
	Other congenital or acquired thrombophilia	0	2	197	1	0.97	NA	0.98	-0.01	0.00	0.99	0.00	0.99
	Stroke (<1 month)	15	5	176	4	0.91	0.77	0.95	0.74	0.79	0.97	0.75	0.98
	Elective major lower extremity arthroplasty	3	1	195	1	0.98	0.75	0.99	0.74	0.75	0.99	0.75	0.99
	Hip, pelvis or leg fracture (<1 month)	14	0	186	0	1.00	1.00	1.00	1.00	1.00	1.00	1.00	1.00
	Acute spinal cord injury (paralysis) (<1 month)	0	1	199	0	0.99	NA	0.99	0.00	NA	0.99	0.00	1.00
	Multiple trauma (<1 month)	6	6	187	1	0.93	0.63	0.96	0.61	0.86	0.97	0.50	0.99

Abbreviations: TP, true positive; FP, false positive; TN, true negative; FN, false negative; Kappa, Cohen's Kappa coefficient; PABAK, Prevalence-Adjusted Bias-Adjusted Kappa; PPV, positive predictive value; NPV, negative predictive value; F1, F1-score; DVT, deep vein thrombosis; PE, pulmonary embolism; BMI, body mass index.

Supplementary Table S20. Macro-Averaged Item-Level Performance of the Padua Score Across Different LLMs and Prompting Strategies in the Test Dataset

Prompt type	LLMs	PABAK	F1-score	Accuracy	Kappa	Time per Case (s)	Tokens per Case
**Basic prompt**	Qwen3-8B	0.78 (95%CI 0.55–0.96)	0.68 (95%CI 0.48–0.86)	0.89 (95%CI 0.77–0.98)	0.58 (95%CI 0.35–0.80)	17.71	7409.3
	GLM-4-9B	0.71 (95%CI 0.51–0.89)	0.60 (95%CI 0.40–0.78)	0.86 (95%CI 0.75–0.94)	0.37 (95%CI 0.15–0.58)	5.13	5221.3
	Qwen3-32B*	0.79 (95%CI 0.53–0.97)	0.74 (95%CI 0.53–0.91)	0.89 (95%CI 0.77–0.98)	0.57 (95%CI 0.33–0.81)	23.63	6787.9
	Deepseek-V3	0.80 (95%CI 0.60–0.97)	0.71 (95%CI 0.47–0.92)	0.90 (95%CI 0.79–0.98)	0.54 (95%CI 0.27–0.80)	12.94	5486.0
	GLM-4.6	0.80 (95%CI 0.58–0.96)	0.74 (95%CI 0.53–0.92)	0.90 (95%CI 0.78–0.98)	0.56 (95%CI 0.30–0.79)	10.96	5741.6
**Complex prompt**	Qwen3-8B	0.78 (95%CI 0.55–0.95)	0.71 (95%CI 0.50–0.89)	0.89 (95%CI 0.78–0.98)	0.53 (95%CI 0.29–0.76)	22.78	8129.1
	GLM-4-9B	0.71 (95%CI 0.51–0.89)	0.51 (95%CI 0.32–0.70)	0.85 (95%CI 0.75–0.94)	0.36 (95%CI 0.16–0.58)	5.18	6735.4
	Qwen3-32B*	0.79 (95%CI 0.55–0.95)	0.72 (95%CI 0.49–0.91)	0.89 (95%CI 0.78–0.97)	0.49 (95%CI 0.25–0.73)	25.38	7507.4
	Deepseek-V3	0.80 (95%CI 0.59–0.96)	0.73 (95%CI 0.54–0.91)	0.90 (95%CI 0.78–0.98)	0.63 (95%CI 0.39–0.84)	13.42	6035.2
	GLM-4.6	0.79 (95%CI 0.56–0.96)	0.72 (95%CI 0.46–0.92)	0.89 (95%CI 0.77–0.98)	0.55 (95%CI 0.28–0.81)	11.20	6314.0
**Expert prompt**	Qwen3-8B	0.81 (95%CI 0.65–0.93)	0.75 (95%CI 0.56–0.84)	0.90 (95%CI 0.84–0.96)	0.60 (95%CI 0.42–0.78)	27.10	10782.1
	GLM-4-9B	0.75 (95%CI 0.59–0.90)	0.44 (95%CI 0.24–0.67)	0.88 (95%CI 0.80–0.95)	0.35 (95%CI 0.15–0.57)	6.89	9865.2
	Qwen3-32B*	0.89 (95%CI 0.79–0.97)	0.80 (95%CI 0.68–0.89)	0.94 (95%CI 0.89–0.98)	0.75 (95%CI 0.61–0.88)	23.9	11003.9
	Deepseek-V3	0.88 (95%CI 0.78–0.97)	0.78 (95%CI 0.65–0.89)	0.94 (95%CI 0.89–0.98)	0.73 (95%CI 0.59–0.86)	13.17	9215.1
	GLM-4.6	0.89 (95%CI 0.79–0.96)	0.84 (95%CI 0.71–0.95)	0.94 (95%CI 0.90–0.98)	0.76 (95%CI 0.65–0.83)	12.80	9633.1

Note * indicates thinking mode; Metrics including Accuracy, Kappa, PABAK, and F1-score were calculated at the item level for each individual Pauda variable and then summarized using macro-averaging across all items.

Abbreviations LLM, Large Language Model; Kappa, Cohen's Kappa coefficient; CI, Confidence Interval; PABAK, Prevalence-Adjusted Bias-Adjusted Kappa; Time per case (s), Average inference time per patient; Tokens per case, average number of input tokens per patient.

Supplementary Table S21. Macro-Averaged Item-Level Performance of the Caprini Score Across Different LLMs and Prompting Strategies in the Test Dataset

Prompt type	LLMs	Accuracy	Kappa	PABAK	F1-score	Time per Case (s)	Tokens per Case
**Basic prompt**	Qwen3-8B	0.94 (95%CI 0.91–0.96)	0.56 (95%CI 0.45–0.68)	0.88 (95%CI 0.83–0.93)	0.68 (95%CI 0.59–0.77)	22.39	7064.6
	GLM-4-9B	0.93 (95%CI 0.90–0.96)	0.41 (95%CI 0.29–0.52)	0.87 (95%CI 0.81–0.92)	0.52 (95%CI 0.40–0.63)	7.14	5886.9
	Qwen3-32B*	0.95 (95%CI 0.92–0.97)	0.65 (95%CI 0.53–0.77)	0.89 (95%CI 0.83–0.94)	0.73 (95%CI 0.63–0.83)	54.12	8247.4
	Deepseek-V3	0.95 (95%CI 0.92–0.97)	0.56 (95%CI 0.44–0.68)	0.90 (95%CI 0.85–0.94)	0.71 (95%CI 0.62–0.79)	18.48	7390.1
	GLM-4.6	0.95 (95%CI 0.92–0.97)	0.60 (95%CI 0.48–0.72)	0.90 (95%CI 0.85–0.95)	0.73 (95%CI 0.63–0.81)	22.15	7047.8
**Complex prompt**	Qwen3-8B	0.94 (95%CI 0.92–0.97)	0.59 (95%CI 0.47–0.70)	0.89 (95%CI 0.84–0.94)	0.68 (95%CI 0.59–0.77)	22.91	9664.3
	GLM-4-9B	0.93 (95%CI 0.90–0.96)	0.42 (95%CI 0.29–0.54)	0.87 (95%CI 0.81–0.92)	0.59 (95%CI 0.48–0.70)	7.70	8343.9
	Qwen3-32B*	0.95 (95%CI 0.92–0.97)	0.67 (95%CI 0.55–0.78)	0.91 (95%CI 0.85–0.95)	0.77 (95%CI 0.68–0.85)	68.92	12131.9
	Deepseek-V3	0.96 (95%CI 0.94–0.98)	0.69 (95%CI 0.56–0.80)	0.92 (95%CI 0.88–0.96)	0.79 (95%CI 0.69–0.87)	27.39	8563.3
	GLM-4.6	0.96 (95%CI 0.93–0.98)	0.65 (95%CI 0.53–0.76)	0.92 (95%CI 0.88–0.96)	0.78 (95%CI 0.69–0.86)	33.31	9576.7
**Expert prompt**	Qwen3-8B	0.94 (95%CI 0.91–0.96)	0.51 (95%CI 0.40–0.63)	0.87 (95%CI 0.81–0.93)	0.65 (95%CI 0.56–0.74)	27.44	10820.64
	GLM-4-9B	0.93 (95%CI 0.90–0.96)	0.47 (95%CI 0.35–0.59)	0.87 (95%CI 0.81–0.92)	0.65 (95%CI 0.54–0.76)	7.70	8343.9
	Qwen3-32B*	0.95 (95%CI 0.92–0.98)	0.69 (95%CI 0.57–0.80)	0.91 (95%CI 0.85–0.96)	0.78 (95%CI 0.67–0.86)	68.91	18534.7
	Deepseek-V3	0.96 (95%CI 0.94–0.98)	0.69 (95%CI 0.56–0.80)	0.92 (95%CI 0.88–0.96)	0.86 (95%CI 0.76–0.90)	23.19	14726.6
	GLM-4.6	0.96 (95%CI 0.94–0.98)	0.69 (95%CI 0.57–0.80)	0.92 (95%CI 0.88–0.96)	0.82 (95%CI 0.73–0.87)	72.21	18378.3

Note * indicates thinking mode; Metrics including Accuracy, Kappa, and PABAK represent the average item-level agreement across all individual Caprini items, with 95% confidence intervals reported.

Abbreviations LLM, Large Language Model; Kappa, Cohen's Kappa coefficient; CI, Confidence Interval; PABAK, Prevalence-Adjusted Bias-Adjusted Kappa; Time per case (s), Average inference time per patient; Tokens per case, average number of input tokens per patient.

Supplementary Table S22. Representative error cases in LLM-based risk factor extraction

Case	Risk item	Expert	Qwen3-Next	Error type
1	Patient confined to bed (>72 hours)	No (Expert rationale: The patient underwent surgery on July 9, 2024 (removal of fibular internal fixation). During the postoperative period until discharge, the medical records repeatedly mentioned orders such as “non-weight bearing on the affected limb” and “avoid strenuous activity”, but there were no explicit statements indicating “absolute bed rest”, “not allowed to get out of bed”, “continuous bed rest >72 hours”, or “bed rest day X”. Meanwhile, the records also included indirect evidence suggesting the patient's ability to be mobile, such as “encourage functional exercise” and “generally in good condition”. Clinically, “non-weight bearing” only indicates restriction of load on the affected limb and does not equate to complete bed rest. Given the lack of direct evidence for sustained bed rest and the presence of indications of mobility, this case does not meet the criterion of “bed rest >72 hours”, and is therefore judged as not meeting the standard.)	Yes (LLM chain-of-thought reasoning: The patient was hospitalized for 5 days post-surgery. The medical records repeatedly emphasized “non-weight bearing on the affected limb”, “elevating the affected limb”, and “dressing the affected limb”, and the medical orders did include “Level 1 care”. This is consistent with “postoperative bed rest observation” and “immobilization for more than three days”. Although there was no explicit record of bed rest for more than three days, the patient did not get out of bed for 5 days post-surgery, clearly exceeding 72 hours.)	Due to incomplete clinical documentation, the LLM made an incorrect inference.
2	Patient confined to bed (>72 hours)	No (Expert rationale: The patient was hospitalized in the respiratory medicine department. Although diagnosed with Pseudomonas aeruginosa pneumonia and hypoxemia, and treated with oxygen therapy, anti-infective therapy, and bronchoalveolar lavage during hospitalization, there were no explicit records indicating “absolute bed rest”, “not allowed to get out of bed”, “continuous bed rest >72 hours”, or “bed rest day X”. Meanwhile, multiple ward round notes described the patient as “conscious and cooperative during examination, in generally fair condition” and noted “mild shortness	Yes (LLM chain-of-thought reasoning: The patient was hospitalized for 10 days. Orders such as “basic VTE prophylaxis”, “mechanical sputum clearance”, “blood pressure monitoring”, “pulse oximetry”, and “central oxygen supply” were interpreted as indicating restricted mobility. There was no documentation of “out-of-bed activity” in the progress notes, and “bronchoscopic airway clearance” was assumed to require the patient to remain in bed. Therefore, the model concluded that bed rest clearly exceeded 72 hours.)	Incomplete clinical documentation and insufficient understanding of medical terminology by the LLM.

Case	Risk item	Expert	Qwen3-Next	Error type
		of breath after activity", suggesting preserved mobility and the ability to ambulate. The medical orders indicated "Level II nursing care", which does not support the need for prolonged bed rest monitoring. Overall, although respiratory function was impaired, there is no direct evidence of sustained bed rest; therefore, the criterion of "bed rest >72 hours" is not met.)		
3	Malignancy (present or previous)	No (Expert rationale: There is no evidence in this patient of any of the following: a current or prior confirmed diagnosis of malignancy; pathological findings indicating malignancy; or receipt of cancer-related treatments during this hospitalization, such as chemotherapy, radiotherapy, targeted therapy, or oncologic surgery. Therefore, the criterion for "malignancy" is not met.)	Yes (LLM chain-of-thought reasoning: The diagnoses do not contain keywords such as "cancer", "malignancy", "leukemia", or "lymphoma". However, multiple orders include tests such as "ascites tumor markers" (CEA, CA199, AFP, NSE, CYFRA21-1, SCC, ProGRP, CA724, CA153), "pleural/ascitic fluid analysis", "cytology/pathology", "bone marrow smear", "bone marrow biopsy", "flow cytometry immunophenotyping", and "cytogenetic karyotyping", suggesting a high suspicion of hematologic or pleural malignancy. In particular, tests like "(external) bone marrow karyotype analysis", "(external) bone marrow biopsy and diagnosis", "(hematology) bone marrow cytology", "abnormal platelet morphology", "pancytopenia", and "secondary thrombocytopenia" are consistent with evaluations for malignancy. Although no explicit diagnosis of "leukemia" or "lymphoma" is documented, malignancy includes entities such as leukemia, lymphoma, and multiple myeloma. While treatments like chemotherapy or immunotherapy are not recorded, the combination of bone marrow biopsy, flow cytometry, and cytogenetics represents the diagnostic gold standard for hematologic malignancies. Together with findings such as pancytopenia, thrombocytopenia, liver dysfunction, and	LLM reasoning error and insufficient prompt constraints.

Case	Risk item	Expert	Qwen3-Next	Error type
			hypoalbuminemia, these Items strongly suggest myelodysplastic syndrome or a pre-leukemic state. Therefore, despite the absence of an explicit diagnosis, the pattern of investigations supports malignancy-related assessment and meets the criteria for “malignancy”.)	
4	Abnormal pulmonary function	No (Expert rationale: The patient underwent tests such as “bronchial provocation test”, “forced oscillation pulmonary function test” and “fractional exhaled nitric oxide measurement” during hospitalization. Although specific test values were not provided, there was no clear diagnosis suggesting pulmonary function abnormality, such as asthma or chronic obstructive pulmonary disease, either previously or during this hospitalization, and no related treatments such as bronchodilators were administered. Therefore, based on the available information, the criterion of “Abnormal pulmonary function” is not met.)	Yes (LLM chain-of-thought reasoning: Orders included “forced oscillation pulmonary function test”, “bronchial provocation test (12 items)” and “fractional exhaled nitric oxide measurement”, suggesting pulmonary function assessment. The patient also had cough, wheezing, moist rales and CT evidence of pneumonia, which clinically suggest airway hyperresponsiveness or ventilatory dysfunction. Although no explicit pulmonary function test values were provided, “bronchial provocation test” and “forced oscillation” are methods for assessing functional abnormalities and therefore meet the criterion of “pulmonary function abnormality”.)	Incomplete clinical data and reasoning error
5	central venous access	Yes (Expert rationale: The patient received “central venous catheter care” during hospitalization, which meets the criterion for central venous access.)	No (LLM chain-of-thought reasoning: Orders included “routine care for deep venous catheterization”, but there was no record of “deep venous catheterization” itself; therefore, actual catheter placement could not be confirmed, and the criterion for central venous catheterization was not met.)	Insufficient prompt constraints and reasoning error
6	Positive lupus anticoagulant	No (Expert rationale: The patient was diagnosed with antiphospholipid antibody syndrome, but no test result for “lupus anticoagulant” was available. Due to the lack of objective evidence, the criterion of “lupus anticoagulant positivity” is not met.)	Yes (LLM chain-of-thought reasoning: The diagnosis explicitly included “antiphospholipid antibody syndrome”, and the medical orders also included “lupus anticoagulant testing (plasma)”. Although “lupus anticoagulant positivity” was not directly documented, the model interpreted “antiphospholipid antibody syndrome” as clinical evidence supporting lupus anticoagulant positivity, and therefore	Incomplete clinical data and reasoning error

Case	Risk item	Expert	Qwen3-Next	Error type
			judged that the criterion of “lupus anticoagulant positivity” was met.)	
7	Major surgery (>45 minutes)	No (Expert rationale: The patient underwent tracheostomy in the ICU. Currently, percutaneous dilatational tracheostomy is more commonly used, with less trauma and faster recovery; therefore, the criterion of “major surgery” is not met.)	Yes (LLM chain-of-thought reasoning: Tracheostomy is a typical invasive procedure involving critical airway anatomy. The operation directly establishes an artificial airway and alters the original anatomical structure, while also carrying risks of complications such as bleeding, infection and pneumothorax. Postoperatively, it often requires prolonged bed rest and mechanical ventilation support, suggesting a significant systemic impact on the patient; therefore, it should be classified as “major open surgery”.)	Insufficient understanding of medical terminology by the LLM
8	Age (≥75 years)	No (Expert rationale: The medical record did not provide the patient’s age or date of birth, making age calculation impossible; therefore, it cannot be determined whether the criterion of “age ≥75 years” is met.)	Yes (LLM chain-of-thought reasoning: The patient was admitted on June 7, 2024, but no date of birth was provided. Based on the history of present illness, including “abdominal pain for one month without obvious cause” and “hemorrhoid surgery performed at another hospital 3 years ago”, together with discharge diagnoses such as “severe mixed ventilatory dysfunction”, “chronic obstructive pulmonary disease” and “old pulmonary tuberculosis”, which are common in older adults, the model inferred age-related degenerative conditions involving multiple systems. In addition, conditions such as “chronic obstructive pulmonary disease, hypertension, prostatic hyperplasia with calcification, hepatic cyst and right renal cyst” are typical in elderly patients. Combined with average life expectancy in the Chinese population and clinical experience, the model judged that the probability of age ≥75 years was high.)	Due to incomplete clinical documentation, the LLM made an incorrect inference.
9	Acute infection and/or rheumatologic disorder	Yes (Expert rationale: The discharge diagnosis included “pneumonia”, which represents a newly developed or active condition during hospitalization. There was no evidence of chronic	No (LLM chain-of-thought reasoning: The discharge diagnosis included “pneumonia”, but there were no records of antibiotic or antiviral therapy, nor evidence of elevated infection markers such as	Incomplete clinical data and insufficient prompt constraints

Case	Risk item	Expert	Qwen3-Next	Error type
		or prior infection; therefore, it was judged as acute infection.)	CRP, PCT or WBC (although high-sensitivity C-reactive protein testing was ordered, no results were reported). Therefore, the criterion for acute infection was not met.)	
10	Heart and/or respiratory failure	No (Expert rationale: Although the diagnoses included “chronic obstructive pulmonary disease with acute lower respiratory tract infection” and “interstitial pneumonia”, and the medical orders included respiratory support treatments such as oxygen therapy and nebulization, there was a lack of key evidence such as a clear diagnosis of respiratory failure, abnormal blood gas analysis or mechanical ventilation; therefore, it cannot be determined as respiratory failure.)	Yes (LLM chain-of-thought reasoning: The diagnosis field included “chronic obstructive pulmonary disease with acute lower respiratory tract infection” and “interstitial pneumonia”, while the medical orders included “nasal catheter oxygen therapy”, “nebulized budesonide and salbutamol” and “routine respiratory care”, which were interpreted as consistent with clinical manifestations and supportive treatment related to respiratory failure.)	Incomplete clinical documentation and insufficient understanding of medical terminology by the LLM

Abbreviations: LLM, large language model; VTE, venous thromboembolism.

Supplementary Table S23. Hospital-Level Performance and Inter-Hospital Variability of the LLM for the Padua Score in the Development Dataset

	PABAK	F1	Accuracy	Kappa	Sensitivity	Specificity	PPV	NPV
**Hospital-level results**								
Center 1	1.00	1.00	1.00	1.00	1.00	1.00	1.00	1.00
Center 2	0.96	1.00	0.98	0.67	0.83	1.00	1.00	0.98
Center 3	0.85	0.91	0.93	0.50	0.83	0.89	0.88	0.98
Center 4	0.96	0.95	0.98	0.91	1.00	0.97	0.93	1.00
Center 5	0.96	1.00	0.98	0.67	0.75	1.00	1.00	0.98
Center 6	0.96	0.98	0.98	0.83	1.00	0.91	0.97	1.00
Center 7	0.96	0.97	0.98	0.89	0.94	1.00	1.00	0.95
Center 8	0.96	1.00	0.98	0.50	0.80	1.00	1.00	0.98
Center 9	0.89	0.91	0.95	0.51	0.72	1.00	1.00	0.86
Center 10	0.93	0.95	0.96	0.82	0.92	1.00	1.00	0.90
**Summary statistics**								
**Mean**	0.95	0.97	0.97	0.73	0.88	0.98	0.98	0.96
**SD**	0.04	0.03	0.02	0.19	0.11	0.04	0.04	0.05
**CV**	0.05	0.04	0.02	0.25	0.12	0.04	0.04	0.05

Note: Performance metrics (accuracy, sensitivity, specificity, PPV, NPV, F1 score, Kappa, and PABAK) were first calculated at the patient level for each item within each hospital. Item-level metrics were then averaged (macro-averaging) to obtain hospital-level performance. Finally, hospital-level estimates were summarized across hospitals using mean, standard deviation (SD), and coefficient of variation (CV) to assess inter-hospital variability. The CV was calculated as the SD divided by the mean. Centers were anonymized and labeled as Center 1, Center 2, etc.

Abbreviations: LLM, large language model; PPV, positive predictive value; NPV, negative predictive value; F1, F1-score; Kappa, Cohen's kappa coefficient; PABAK, prevalence-adjusted bias-adjusted kappa; SD, standard deviation; CV, coefficient of variation.

Supplementary Table S24. Hospital-level performance and inter-hospital variability of the LLM for the Caprini Score in the Development Dataset

	PABAK	F1	Accuracy	Kappa	Sensitivity	Specificity	PPV	NPV
**Hospital-level results**								
Center 1	0.94	0.95	0.97	0.72	0.86	0.99	0.93	0.94
Center 2	0.93	0.94	0.96	0.62	0.87	0.98	0.85	0.96
Center 3	0.77	0.91	0.88	0.42	0.59	0.96	0.75	0.90
Center 4	0.95	0.93	0.97	0.82	0.88	0.99	0.97	0.97
Center 5	0.91	0.84	0.95	0.55	0.72	0.99	0.85	0.94
Center 6	0.94	0.91	0.97	0.80	0.91	0.98	0.89	0.98
Center 7	0.92	0.90	0.96	0.66	0.73	0.99	0.92	0.95
Center 8	0.87	0.91	0.94	0.58	0.74	0.99	0.92	0.90
Center 9	0.95	0.88	0.97	0.37	0.90	0.97	0.62	0.99
Center 10	0.91	0.88	0.95	0.66	0.83	0.99	0.92	0.93
**Summary statistics**								
**Mean**	0.91	0.90	0.95	0.62	0.80	0.98	0.86	0.95
**SD**	0.05	0.03	0.03	0.15	0.10	0.01	0.11	0.03
**CV**	0.06	0.04	0.03	0.24	0.13	0.01	0.12	0.03

Notes: Performance metrics (accuracy, sensitivity, specificity, PPV, NPV, F1 score, Kappa, and PABAK) were first calculated at the patient level for each item within each hospital. Item-level metrics were then averaged (macro-averaging) to obtain hospital-level performance. Finally, hospital-level estimates were summarized across hospitals using mean, standard deviation (SD), and coefficient of variation (CV) to assess inter-hospital variability. The CV was calculated as the SD divided by the mean. Centers were anonymized and labeled as Center 1, Center 2, etc.

Abbreviations: LLM, large language model; PPV, positive predictive value; NPV, negative predictive value; F1, F1-score; Kappa, Cohen's kappa coefficient; PABAK, prevalence-adjusted bias-adjusted kappa; SD, standard deviation; CV, coefficient of variation.

Supplementary Table S25. Hospital-level performance and inter-hospital variability of the LLM for the Padua score in the test Dataset

	PABAK	F1	Accuracy	Kappa	Sensitivity	Specificity	PPV	NPV
**Hospital-level results**								
Center 11	1.00	1.00	1.00	1.00	1.00	1.00	1.00	1.00
Center 12	0.98	1.00	0.99	0.83	1.00	0.99	0.83	1.00
Center 13	0.98	0.99	0.99	0.97	1.00	0.98	0.98	1.00
Center 14	1.00	1.00	1.00	1.00	1.00	1.00	1.00	1.00
Center 15	0.95	0.96	0.97	0.80	0.94	1.00	1.00	0.91
Center 16	0.98	0.97	0.99	0.93	1.00	0.98	0.94	1.00
Center 17	0.98	0.98	0.99	0.97	1.00	0.98	0.97	1.00
Center 18	0.98	0.98	0.99	0.97	0.97	1.00	1.00	0.98
Center 19	0.93	0.92	0.96	0.88	0.89	0.98	0.98	0.96
Center 20	0.91	0.93	0.95	0.73	0.95	0.95	0.77	0.98
Center 21	0.98	0.95	0.99	0.95	1.00	0.99	0.93	1.00
Center 22	1.00	1.00	1.00	1.00	1.00	1.00	1.00	1.00
Center 23	1.00	1.00	1.00	1.00	1.00	1.00	1.00	1.00
Center 24	1.00	1.00	1.00	1.00	1.00	1.00	1.00	1.00
Center 25	1.00	1.00	1.00	1.00	1.00	1.00	1.00	1.00
Center 26	0.95	0.94	0.97	0.76	0.79	0.99	0.95	0.98
Center 27	0.98	0.97	0.99	0.96	0.95	1.00	1.00	0.99
Center 28	1.00	1.00	1.00	1.00	1.00	1.00	1.00	1.00
Center 29	0.89	0.91	0.95	0.63	0.86	0.99	0.80	0.92
Center 30	0.95	0.95	0.97	0.91	0.98	0.97	0.94	0.98
**Summary statistics**								
**Mean**	0.97	0.97	0.99	0.91	0.97	0.99	0.95	0.99
**SD**	0.03	0.03	0.02	0.11	0.06	0.01	0.07	0.03
**CV**	0.03	0.03	0.02	0.12	0.06	0.01	0.08	0.03

Notes: Performance metrics (accuracy, sensitivity, specificity, PPV, NPV, F1 score, Kappa, and PABAK) were first calculated at the patient level for each item within each

hospital. Item-level metrics were then averaged (macro-averaging) to obtain hospital-level performance. Finally, hospital-level estimates were summarized across hospitals using mean, standard deviation (SD), and coefficient of variation (CV) to assess inter-hospital variability. The CV was calculated as the SD divided by the mean. Centers were anonymized and labeled as Center 1, Center 2, etc.

Abbreviations: LLM, large language model; PPV, positive predictive value; NPV, negative predictive value; F1, F1-score; Kappa, Cohen's kappa coefficient; PABAK, prevalence-adjusted bias-adjusted kappa; SD, standard deviation; CV, coefficient of variation.

Supplementary Table S26. Hospital-level performance and inter-hospital variability of the LLM for the Caprini score in the test Dataset

	PABAK	F1	Accuracy	Kappa	Sensitivity	Specificity	PPV	NPV
**Hospital-level results**								
Center 11	0.95	0.88	0.98	0.83	0.93	0.98	0.88	0.99
Center 12	0.96	0.95	0.98	0.84	0.92	0.98	0.92	0.99
Center 13	0.96	0.92	0.98	0.78	0.91	0.98	0.85	0.99
Center 14	0.97	0.93	0.98	0.73	0.85	0.98	0.88	0.99
Center 15	0.97	0.94	0.99	0.91	0.96	0.99	0.94	0.99
Center 16	0.91	0.90	0.96	0.67	0.76	0.98	0.87	0.96
Center 17	0.95	0.96	0.98	0.74	0.85	0.97	0.89	0.99
Center 18	0.96	0.93	0.98	0.80	0.88	0.97	0.93	0.99
Center 19	0.98	0.96	0.99	0.94	0.96	1.00	0.97	0.99
Center 20	0.98	1.00	0.99	0.79	0.92	0.97	0.92	0.99
Center 21	0.96	0.91	0.98	0.83	0.91	0.99	0.89	0.99
Center 22	0.99	0.99	0.99	0.97	0.99	0.99	0.99	0.99
Center 23	0.98	0.94	0.99	0.93	0.92	1.00	1.00	0.99
Center 24	0.99	1.00	1.00	0.94	0.94	1.00	1.00	1.00
Center 25	0.99	0.98	0.99	0.96	1.00	0.99	0.97	1.00
Center 26	0.95	0.93	0.98	0.81	0.87	0.97	0.95	0.99
Center 27	0.96	0.93	0.98	0.89	0.97	0.97	0.93	1.00
Center 28	0.96	0.94	0.98	0.85	1.00	0.98	0.85	1.00
Center 29	0.99	0.99	1.00	0.98	1.00	1.00	0.98	1.00
Center 30	0.96	0.94	0.98	0.80	0.93	0.97	0.90	0.99
**Summary statistics**								
**Mean**	0.97	0.95	0.98	0.85	0.92	0.98	0.93	0.99
**SD**	0.02	0.03	0.01	0.09	0.06	0.01	0.05	0.01
**CV**	0.02	0.03	0.01	0.10	0.07	0.01	0.05	0.01

Notes: Performance metrics (accuracy, sensitivity, specificity, PPV, NPV, F1 score, Kappa, and PABAK) were first calculated at the patient level for each item within each hospital. Item-level metrics were then averaged (macro-averaging) to obtain hospital-level performance. Finally, hospital-level estimates were summarized across hospitals using mean, standard deviation (SD), and coefficient of variation (CV) to assess inter-hospital variability. The CV was calculated as the SD divided by the mean. Centers were anonymized and labeled as Center 1, Center 2, etc.

Abbreviations: LLM, large language model; PPV, positive predictive value; NPV, negative predictive value; F1, F1-score; Kappa, Cohen's kappa coefficient; PABAK, prevalence-adjusted bias-adjusted kappa; SD, standard deviation; CV, coefficient of variation.

Supplementary Information

The original prompt templates were developed in Chinese to align with the language of the clinical records. To facilitate reproducibility and accessibility for an international audience, we provide English-translated versions of the prompts in this Supplementary Information.

Prompt template for Padua score extraction

1. Padua Scoring Basic Prompt

[Task Background]
You are a medical information extraction assistant skilled in identifying venous thromboembolism (VTE)-related risk factors from Chinese electronic medical records.

[Task Objective]
Based on the patient's hospitalization medical record, determine whether the patient meets each risk factor in the Padua VTE Risk Score.

[Input Data]
The input is the patient's complete hospitalization medical record text, which may include: past medical history, history of present illness, diagnoses, physical examination findings, laboratory results, medical orders, surgical records, discharge summary, and so on.

[Decision Rules]
1. If the medical record contains an explicit description that meets the definition, label it as 1.
2. If it is not mentioned, described unclearly, or cannot be determined, label it as 0.
3. Base judgments only on the original text; do not over-infer from tests, medications, or common knowledge.
4. A justification must be provided for every variable.
5. The justification should quote key information from the original text whenever possible; if the original text does not mention it, explicitly write "Not mentioned".

[Padua Risk Factors]
1. Active cancer
2. Previous VTE
3. Reduced mobility (≥3 days)
4. Ongoing hormonal treatment
5. Already known thrombophilic condition
6. Recent (≤1 month) trauma and/or surgery
7. Elderly age (≥70 years)
8. Heart and/or respiratory failure
9. Acute myocardial infarction or ischemic stroke
10. Acute infection and/or rheumatologic disorder
11. Obesity (BMI ≥30)

[Important Constraints]

1. Every variable must be output.
2. No variable may be added or omitted.
3. The output must be valid JSON.
4. Nothing other than JSON may appear in the output.
5. Every variable in reasoning must have content.
6. If there is no supporting evidence in the original text, write "Not mentioned" in reasoning.

[Output Format]

{
"subject_id": "<subject_id>",
"result": [
{"item": "Active cancer", "value": 0},
{"item": "Previous VTE", "value": 0},
{"item": " Reduced mobility (≥3 days)", "value": 0},
{"item": "Ongoing hormonal treatment", "value": 0},
{"item": "Already known thrombophilic condition", "value": 0},
{"item": "Recent (≤1 month) trauma and/or surgery", "value": 0},
{"item": "Elderly age (≥70 years)", "value": 0},
{"item": "Heart and/or respiratory failure", "value": 0},
{"item": "Acute myocardial infarction or ischemic stroke", "value": 0},
{"item": "Acute infection and/or rheumatologic disorder", "value": 0},
{"item": "Obesity (BMI ≥30)", "value": 0}
],
"reasoning": [
{"item": "Active cancer", "reason": "The original text does not mention any malignancy-related diagnosis, recurrence, metastasis, or antitumor treatment; therefore, this is judged as 0."},
{"item": "Previous VTE", "reason": "The original text does not mention a history of deep vein thrombosis, pulmonary embolism, or other VTE; therefore, this is judged as 0."},
{"item": " Reduced mobility (≥3 days)", "reason": "The medical record states that the patient was bedridden for 3 days; therefore, this is judged as 1."},
{"item": "Ongoing hormonal treatment", "reason": "The original text does not mention oral contraceptives or hormone replacement therapy; therefore, this is judged as 0."},
{"item": "Already known thrombophilic condition", "reason": "The original text does not mention antiphospholipid syndrome, protein C deficiency, protein S deficiency, antithrombin deficiency, Factor V Leiden, or similar thrombophilic conditions; therefore, this is judged as 0."},
{"item": "Recent (≤1 month) trauma and/or surgery", "reason": "The original text does not mention recent trauma or surgery; therefore, this is judged as 0."},
{"item": "Elderly age (≥70 years)", "reason": "The patient is 78 years old; therefore, this is judged as 1."},
{"item": "Heart and/or respiratory failure", "reason": "The original text does not mention a definite diagnosis of heart failure or respiratory failure; therefore, this is judged as 0."},
{"item": "Acute myocardial infarction or ischemic stroke", "reason": "The original text does not mention acute myocardial infarction or ischemic stroke; therefore, this is judged as 0."},
{"item": "Acute infection and/or rheumatologic disorder", "reason": "The original text does not mention a definite diagnosis of acute infection or rheumatologic disorder; therefore, this is judged as 0."},

{"item": "Obesity (BMI ≥30)", "reason": "The BMI is 24.2, which does not reach 30; therefore, this is judged as 0."}
]
}

[Medical Record Text]
{input_text}
---

2. Padua Scoring Complex Prompt
[Task Background]
You are a medical information extraction assistant responsible for identifying venous thromboembolism (VTE)-related risk factors from Chinese electronic medical records.

[Task Objective]
Based on the patient's hospitalization medical record, determine whether the patient meets each risk factor in the Padua score.
Only determine whether each variable is present; do not assign points and do not calculate the total score.

[Input Data]
The input is the patient's complete hospitalization medical record text, which may include past medical history, history of present illness, diagnoses, physical examination findings, laboratory tests, medical orders, surgical records, discharge summary, and so on.

[Decision Rules]
1. If there is an explicit description in the medical record and it meets the definition, label it as 1.
2. If it is not mentioned, is unclear, or cannot be determined, label it as 0.
3. Base judgments only on the original text; do not over-infer from tests, medications, or common knowledge.
4. A justification must be provided for every variable.

[Padua Risk Factors and Decision Points]
1. Active cancer
[Medical Definition]: Refers to a patient who is currently receiving cancer-related treatment (chemotherapy, radiotherapy, targeted therapy, immunotherapy, etc.), or who was diagnosed with a malignant tumor within the past 6 months, or who has known metastatic disease.
[Examples]:
- "The patient received chemotherapy for a malignant tumor during this hospitalization." indicates this item is present.
- "History of breast cancer, cured many years ago." indicates this item is absent.
- "A space-occupying lesion was found but its nature has not yet been confirmed." indicates this item is absent.

---

2. Previous VTE
[Medical Definition]: A prior history of deep vein thrombosis (DVT) of the lower extremities or pulmonary embolism (PE).
[Examples]:
- “History of pulmonary embolism.” indicates this item is present.
- “Denies history of thrombosis.” indicates this item is absent.
- “No record related to previous VTE history is found in the past medical history.” indicates this item is absent.

---
3. Reduced mobility ≥3 days
[Medical Definition]: Bed rest with bathroom privileges only (due to the patient’s own limitations or medical orders) lasting for at least 3 days.
[Examples]:
- “Bed rest for 72 hours after surgery.” indicates this item is present.
- “The patient moves freely.” indicates this item is absent.
- “The patient got out of bed on postoperative day 2.” indicates this item is absent.

---
4. Ongoing hormonal treatment
[Medical Definition]: Receipt of any estrogen-containing treatment, including oral contraceptives and postmenopausal hormone replacement therapy.
[Examples]:
- “Currently taking oral contraceptives.” indicates this item is present.
- “No use of estrogen-progestin drugs.” indicates this item is absent.
- “Medication was stopped three months ago.” indicates this item is absent.

---
5. Already known thrombophilic condition
[Medical Definition]: Includes antithrombin deficiency, protein C deficiency, protein S deficiency, Factor V Leiden mutation, prothrombin G20210A mutation, and antiphospholipid syndrome.
[Examples]:
- “Diagnosed with antiphospholipid syndrome.” indicates this item is present.
- “Protein C test showed no abnormality.” indicates this item is absent.
- “No related testing has been performed.” indicates this item is absent.

---
6. Recent trauma and/or surgery
[Medical Definition]: Any traumatic surgery within the past 30 days, or any major traumatic event such as fracture or traffic accident.
[Examples]:
- “The patient underwent knee replacement surgery three days before admission.” indicates this item is present.
- “Denies surgical history.” indicates this item is absent.
- “Appendectomy 10 years ago.” indicates this item is absent.

---
7. Elderly age (≥70 years)
[Examples]:
- “82 years old.” indicates this item is present.
- “64 years old.” indicates this item is absent.
- “69 years old.” indicates this item is absent.

---
8. Heart and/or respiratory failure
[Examples]:
- “Diagnosed with respiratory failure during this hospitalization and underwent endotracheal intubation.” indicates this item is present.
- “Good cardiac function, no dyspnea.” indicates this item is absent.
- “Shortness of breath only, without a diagnosis.” indicates this item is absent.

---
9. Acute myocardial infarction or ischemic stroke
[Examples]:
- “Admitted for acute cerebral infarction.” indicates this item is present.
- “No relevant medical history.” indicates this item is absent.
- “Cerebral infarction one year ago.” indicates this item is absent.

---
10. Acute infection and/or rheumatologic disorder
[Medical Definition]: Currently in an acute infectious state, or active rheumatologic disease (such as active systemic lupus erythematosus).
[Examples]:
- “Diagnosed with pulmonary infection.” indicates this item is present.
- “The patient has no fever and no other evidence of infection.” indicates this item is absent.
- “Rheumatoid arthritis for many years, currently stable.” indicates this item is absent.

---
11. Obesity (BMI ≥30)
[Medical Definition]: BMI ≥30
[Examples]:
- “BMI 33.” indicates this item is present.
- “BMI 28.” indicates this item is absent.
- “BMI not mentioned.” indicates this item is absent.

[Important Constraints]
1. Every variable must be output.
2. No variables may be added or omitted.
3. The output must be valid JSON.
4. Nothing other than JSON may appear in the output.
5. A justification must be provided for every variable.

[Output Format]

```
{
  "subject_id": "<subject_id>",
  "result": [
    {"item": "Active cancer", "value": 0},
    {"item": "Previous VTE", "value": 0},
    {"item": "Reduced mobility", "value": 0},
    {"item": "Ongoing hormonal treatment", "value": 0},
    {"item": "Already known thrombophilic condition", "value": 0},
    {"item": "Recent trauma and/or surgery", "value": 0},
    {"item": "Elderly age (≥70 years)", "value": 0},
    {"item": "Heart and/or respiratory failure", "value": 0},
    {"item": "Acute myocardial infarction or ischemic stroke", "value": 0},
    {"item": "Acute infection and/or rheumatologic disorder", "value": 0},
    {"item": "Obesity (BMI ≥30)", "value": 0}
  ],
  "reasoning": [
    {"item": "Active cancer", "reason": "No related diagnosis or treatment mentioned"},
    {"item": "Previous VTE", "reason": "No history of thrombosis mentioned"}
  ]
}
```

[Medical Record Text]
{input_text}

---

3. Padua Scoring Expert knowledge-augmented prompt

[Task Background]
You are a medical information extraction assistant and need to identify venous thromboembolism (VTE)-related risk factors from Chinese electronic medical records.

[Task Objective]
Based on the patient's hospitalization medical record, determine whether the patient meets each Padua risk factor.
Only determine whether each variable is present (1 or 0); do not assign points, do not calculate the total score, and do not perform risk stratification.

[Input Data]
Includes past medical history, history of present illness, diagnoses, laboratory tests, medical orders, surgical records, nursing records, etc.

[General Decision Principles (must be followed)]
1. Base judgments only on the original text; do not infer

2. A variable can be judged as 1 only when there is explicit evidence
3. A single clue (such as only a laboratory test or only a medication) is usually insufficient for judgment
4. If not mentioned or if evidence is insufficient, judge as 0
5. Every variable must be accompanied by a justification
6. Information from different fields may be combined for judgment (except for items with special restrictions)

Item 1: Active cancer
[Medical Definition]: Refers to a patient who is currently receiving cancer-related treatment (chemotherapy, radiotherapy, targeted therapy, immunotherapy, etc.), or who was diagnosed with a malignant tumor within the past 6 months, or who has known metastasis.
[Sources]: Diagnosis fields (outpatient/emergency diagnosis, primary diagnosis, other diagnoses), surgery and procedures, pathology diagnosis, medical orders, past medical history
[Keywords]
Diagnosis keywords: metastatic malignant tumor, cancer, malignant tumor, sarcoma, leukemia, lymphoma, multiple myeloma
Surgery keywords: tumor resection, sarcoma resection, cancer resection, palliative surgery for malignant tumors, lymph node dissection, radiotherapy
Pathology keywords: cancer, malignant tumor, sarcoma
Medication keywords: paclitaxel, docetaxel, cisplatin, carboplatin, oxaliplatin, doxorubicin, epirubicin, irinotecan, topotecan, cyclophosphamide, gemcitabine, fluorouracil, etoposide, cytarabine, capecitabine, vincristine, vindesine, vinorelbine, targeted therapy, targeted drugs, targeted regimen, imatinib, erlotinib, gefitinib, apatinib, lenvatinib, axitinib, sorafenib, sunitinib, bevacizumab, almonertinib, trastuzumab, dabrafenib, immunotherapy, immunosuppressants, immune checkpoint inhibitors, nivolumab, pembrolizumab, tislelizumab, atezolizumab, ipilimumab, tamoxifen, anastrozole, letrozole, goserelin, leuprorelin, bicalutamide, thalidomide, lenalidomide, carfilzomib, bleomycin, intrathoracic perfusion chemotherapy, methotrexate, pemetrexed, hydroxyurea, raltitrexed, mercaptopurine, rituximab, bortezomib, olaparib, axicabtagene, lobaplatin, nedaplatin, homoharringtonine, temozolomide
[Decision Criteria] (must satisfy both “evidence of malignancy” and “evidence of activity”)
Step 1: Confirm evidence of malignancy (any of the following)
- Malignancy-related keywords explicitly appear in diagnosis fields
- Pathology diagnosis clearly indicates malignancy
- Past medical history explicitly indicates malignancy
Step 2: Confirm active status (any of the following)
- Current or recent treatment exists (chemotherapy, radiotherapy, targeted therapy, immunotherapy, etc.)
- Metastasis is present
- Past medical history explicitly indicates treatment within 6 months before admission
[Decision Rules]
- Judge as 1 only when both “malignancy exists” and “active status exists” are satisfied
- If malignancy exists but there is no evidence of activity, judge as 0
- If not mentioned or evidence is insufficient, judge as 0
[Notes]
1. If only “cancer” appears but there is no treatment, metastasis, or time information, do not judge it as active
2. Past medical history counts only when it is clearly within 6 months

3. Joint judgment across multiple fields is allowed

--

Item 2: Previous VTE

[Medical Definition]: Previous lower-extremity deep vein thrombosis (DVT) or pulmonary embolism (PE).

[Sources]: Past medical history field only

[Keywords]: VTE, DVT, PE, venous thrombosis, pulmonary embolism, pulmonary artery thrombosis

[Exclusion Keywords]: no history of VTE, superficial lower-extremity vein, phlebitis (such as basilic vein, great saphenous vein, etc.)

[Decision Criteria]

All of the following must be met:

1. The information must come from the past medical history field
2. The past medical history explicitly records VTE-related history
3. It is not superficial vein thrombosis or phlebitis
4. It is not first diagnosed during the current hospitalization

[Decision Rules]

Judge as 1 only when all the above are satisfied

If explicitly stated as “no history of VTE,” judge as 0

If the source is not the past medical history field, or if it is newly developed this admission, judge as 0

If not mentioned or evidence is insufficient, judge as 0

--

Item 3: Elderly age (≥70 years)

[Item]: Elderly age (≥70 years)

[Sources]: Age field or date of birth field

[Keywords]: age 70 or above, age ≥70

[Decision Criteria]

Any one of the following is sufficient:

1. The age field explicitly shows ≥70 years
2. Age calculated from admission date minus date of birth is ≥70 years

[Decision Rules]

Judge as 1 when the criterion is met

Judge as 0 when it is not met

If not mentioned or cannot be calculated, judge as 0

[Notes]

1. Prefer the structured age field
2. If both age and date of birth are present, prioritize the structured age field
3. Date of birth must be combined with admission date to calculate age
4. Do not judge based only on vague descriptions such as “elderly”

Item 4: Heart and/or respiratory failure

[Sources] Diagnosis fields, medical order fields

[Keywords]

Heart failure, chronic cardiac insufficiency, Adams-Stokes syndrome, cardiogenic shock, acute

pulmonary edema, acute heart failure, cardiopulmonary resuscitation, cardiac arrest, cardiac function class IV, respiratory failure, ventilator, endotracheal intubation, high-flow oxygen therapy
[Decision Criteria]
Any one of the following is sufficient:
1. Diagnosis fields explicitly contain a diagnosis related to heart failure or respiratory failure
2. Medical orders or treatments contain the following supportive measures:
  - Noninvasive ventilator, mechanical ventilation, endotracheal intubation, ECMO
  - High-flow oxygen therapy
  - Cardiopulmonary resuscitation or management related to cardiac arrest
[Decision Rules]
Judge as 1 when the above criteria are met
Judge as 0 when they are not met
If not mentioned or evidence is insufficient, judge as 0
[Notes]
1. Laboratory results (such as elevated BNP) cannot be used alone as evidence
2. Symptoms alone (such as shortness of breath or dyspnea) without a clear diagnosis or supportive treatment do not count
3. Priority should be given to explicit diagnoses, then to key supportive treatments
4. Joint judgment across multiple sources is allowed

Item 5: Acute infection and/or rheumatologic disorder
[Medical Definition] Acute infection and/or rheumatologic disorder refers to a recent acute systemic or local infection, or a definite systemic rheumatologic/immune disease, in which inflammation and treatment increase hypercoagulability risk.
[Sources] Primary/other diagnosis fields, medical order fields, laboratory fields
[Keywords]
Acute infection-related: sepsis, bacteremia, septicemia, septic shock, pneumonia, severe pneumonia, pulmonary infection, fungal infection, respiratory infection, acute bronchitis, COVID-19, pyelonephritis, cystitis, urinary tract infection, acute prostatitis, acute gastroenteritis, cellulitis, erysipelas, abscess, bacterial meningitis, cholecystitis, cholangitis, myelitis, infective endocarditis, infectious fever, peritonitis, acute hepatitis, acute viral enteritis, acute bacterial enteritis, acute mastitis, acute pancreatitis, osteomyelitis, septic arthritis
Rheumatologic disease-related: rheumatoid arthritis, lupus, Sjögren syndrome, Behçet disease, dermatomyositis, scleroderma, polymyositis, vasculitis, ankylosing spondylitis, psoriatic arthritis, amyopathic dermatomyositis, antisynthetase syndrome, aortitis
Medication-related: antibiotics, antiviral drugs; glucocorticoids or immunosuppressants
Laboratory-related: elevated white blood cell count, elevated CRP, elevated PCT, elevated ESR, positive cultures
[Exclusion Keywords]
Chronic, Helicobacter (for example, “chronic pneumonia” or “Helicobacter pylori” do not count)
[Decision Criteria]
Any one of the following:
1. The diagnosis field contains an acute infection-related disease, and at the same time at least one of the following supporting evidences is present:
  - Anti-infective treatment in medical orders (antibiotics or antiviral drugs)
  - Laboratory evidence suggestive of infection (elevated white blood cells, CRP, PCT, ESR, or

positive culture)
  - Infection is the main reason for the current hospitalization
2. The diagnosis field contains a rheumatologic disease, and at the same time at least one of the following supporting evidences is present:
  - Use of immunosuppressants, cytotoxic drugs, or glucocorticoid therapy
  - The main reason for the current hospitalization is treatment of the rheumatologic disease

[Decision Rules]
Judge as 1 when the decision criteria are met
Judge as 0 when they are not met
If not mentioned or evidence is insufficient, judge as 0
[Notes]
1. Both "diagnosis" and "supporting evidence" must be present; a single clue is insufficient
2. Fever alone or inflammatory marker abnormalities alone cannot be used as evidence
3. Chronic infections do not count
4. Joint judgment across multiple sources is allowed

Item 6: Obesity (BMI ≥30)
[Medical Definition] BMI ≥30
[Sources] BMI field or height and weight
[Decision Rules]
BMI ≥30 → judge as 1
BMI <30 → judge as 0
If not mentioned or cannot be calculated → judge as 0

Item 7: Ongoing hormonal treatment
[Medical Definition] Refers to sex hormone-related treatment, including estrogen or estrogen-progestin combination therapy, used for contraception, menopausal hormone replacement, assisted reproduction, or breast/gynecologic tumor treatment; glucocorticoids are excluded.
[Sources] Medical orders, past medical history, marital/reproductive history
[Keywords]
Estrogen-related: estrogen, estradiol, ethinyl estradiol, Progynova
Progestin-related (for contraception or replacement): dydrogesterone, progesterone, megestrol acetate
SERMs: raloxifene, tamoxifen
Aromatase inhibitors: anastrozole, letrozole
Contraception and assisted reproduction-related: oral contraceptives, combination preparations, assisted reproduction drugs
[Exclusion Keywords] prednisone, methylprednisolone, dexamethasone, and other glucocorticoids
[Decision Criteria]
Any one of the following:
1. Medical orders or past medical history explicitly indicate use of estrogen or estrogen-progestin-related drugs
2. Marital/reproductive history explicitly records contraceptive drugs, oral contraceptives, or assisted reproduction drugs

[Decision Rules]
Judge as 1 when the criterion is met
Judge as 0 when it is not met

If not mentioned or evidence is insufficient, judge as 0
[Notes]
1. Glucocorticoids do not count
2. Progestin-only preparations generally do not count unless explicitly used for contraception or assisted reproduction
3. There must be actual medication use or a clear history of use; do not judge based on possibility alone
4. Joint judgment across multiple sources is allowed

Item 8: Recent trauma and/or surgery
[Medical Definition]: Trauma (such as fracture, solid organ injury, closed/open injury, etc.) or surgery within ≤30 days before admission, including surgery under general anesthesia, neuraxial anesthesia, regional anesthesia, or intravenous anesthesia; simple diagnostic endoscopy or puncture biopsy and similar minimally invasive diagnostic/therapeutic procedures are excluded.
[Sources]: Past medical history field only
[Keywords]:
Trauma-related: trauma, injury, fracture, crack fracture, open wound, contusion, car accident/traffic accident (MVC), fall injury, liver/spleen rupture, renal contusion, intracavitary/solid organ injury, laceration
Surgery-related: surgery, surgical history, ...ectomy / ...repair / ...replacement / internal fixation / plate / intramedullary nail / suture / cesarean section / cholecystectomy / appendectomy / tumor resection / arthroscopy / open reduction and internal fixation, etc.
Postoperative indicators: postoperative, postoperative day X, suture removal, postoperative review
[Exclusion Keywords / Situations (any one excludes the item)]:
Explicit negation/none: no surgical history, denies surgery/trauma
Not from past medical history: information from surgical records/progress notes/diagnoses or other current hospitalization process information (not allowed for this item)
Time inconsistent or unclear: several years ago, many years ago, long-standing past history, no date and no relative time to infer; only “recently” or “the other day” without confirmation of ≤30 days
Planned but not performed: planned / proposed / considered / recommended surgery, preoperative assessment / preparation but not yet performed
Examinations or therapeutic minimally invasive procedures that usually do not count as surgery: gastroscopy/colonoscopy, bronchoscopy, cystoscopy, hysteroscopy; puncture/biopsy/catheter placement (PICC/central venous catheter) as diagnostic/therapeutic procedures only, unless clearly documented as surgical treatment
[Time Judgment (must be met)]:
Absolute date: if the past medical history provides the date of trauma/surgery, admission date - event date must be ≤30 days (including day 30).
Relative expressions that can be judged: such as “postoperative day X,” “X days/weeks/months ago”; if ≤30 days can be inferred from the admission date, it counts.
Only “recently / the other day / not long ago” without calculable evidence → does not count.
If both “trauma” and “surgery” appear with different dates, a value of 1 can be assigned as long as either event meets the ≤30-day requirement.
[Scoring Rule]: When all of the following are satisfied: (source = past medical history) + (keyword hit and no exclusion triggered) + (time ≤30 days), recent trauma and/or surgery = 1
[Notes]:
This item is strictly limited to the “past medical history” source. Preoperative preparation / procedures /

examinations during the current hospitalization do not count.
Endoscopic “operations” (such as endoscopic mucosal resection/hemostasis/polypectomy) may count if clearly documented as therapeutic surgery of a surgical nature and within ≤30 days; diagnostic endoscopy alone does not count.
If “postoperative follow-up / suture removal / review” is mentioned but it is impossible to infer that the event occurred within ≤30 days, do not count it based on the word “postoperative” alone.

Item 9: Reduced mobility
[Medical Definition] Bed rest with bathroom privileges only (due to the patient’s own limitations or medical orders) for at least 3 days.
[Sources]: Nursing records, medical orders, progress notes, physical examination, admission/transfer information, diagnosis fields, surgical records
[Keywords]:
Nursing/orders/progress notes: absolute bed rest, strict bed rest, bed rest ≥72 hours, prolonged bed rest, bed-rest care, turning care, mechanical ventilation, endotracheal intubation, ECMO, sedation and analgesia, pressure sore care
Physical examination: lower limb muscle strength grade 0, consciousness disturbance, coma, lower limb paralysis
Departments: ICU, CCU, RICU, EICU, intensive care unit, stroke center
Severe diseases/surgeries: hip fracture, femoral fracture, pelvic fracture, spinal fracture, cerebral contusion/laceration, intracranial hemorrhage, multiple trauma, fall from height, laparotomy, thoracotomy, craniotomy, organ transplantation, extensive burns, joint replacement, etc.
[Criteria] (any one of the following):
1. Explicit description of bed rest or mobility restriction
Such as “absolute bed rest,” “bed rest ≥72 hours after surgery,” “requires bed-rest care,” “pressure sore care,” etc.
2. Explicit loss of function
Such as lower limb muscle strength grade 0, coma, consciousness disturbance
3. Strong supportive treatment indicating inability to move independently
Such as mechanical ventilation, endotracheal intubation, ECMO, deep sedation
4. Severe injury or major surgery usually causing bed rest
Such as spinal fracture, pelvic fracture, laparotomy, thoracotomy, craniotomy, organ transplantation, etc.
5. If the above are not met, the patient’s severe condition and self-limitation require more than 3 days
[Decision Rules]
Judge as 1 when the criteria are met
Judge as 0 when they are not met
If not mentioned or evidence is insufficient, judge as 0
[Notes]
1. Severe illness or monitoring alone cannot directly imply reduced mobility
2. Department of Respiratory and Critical Care Medicine belongs to a general ward and does not count
3. Joint judgment across multiple sources is allowed

Item 10: Acute myocardial infarction or ischemic stroke
[Medical Definition] Refers to current or recent ischemic stroke or acute myocardial infarction; old events or hemorrhagic stroke are excluded.

[Sources] Diagnosis fields, past medical history, history of present illness
[Keywords]: acute myocardial infarction (all types), cerebral infarction, cerebral thrombosis, cerebral arterial thrombosis, cerebral infarction/stroke
[Exclusion Keywords]: old, previous, sequelae, lacunar, cerebral hemorrhage, subarachnoid hemorrhage, hemorrhagic stroke, personal history
[Criteria] Both of the following must be met:
1. There is a diagnosis related to ischemic stroke or myocardial infarction
2. It is a current or recent acute event (already present at admission or newly developed during hospitalization)
[Decision Rules]
Judge as 1 when the criteria are met
Judge as 0 when they are not met
If not mentioned or evidence is insufficient, judge as 0
[Notes]
1. Includes events present at admission, events occurring within 30 days before admission, or new events during hospitalization
2. Old cerebral infarction or sequelae do not count
3. Hemorrhagic stroke (cerebral hemorrhage, subarachnoid hemorrhage, etc.) is explicitly excluded
4. If only “cerebral infarction” appears without time information, it should preferentially be judged as a current event unless clearly indicated as previous or old
5. Joint judgment across multiple sources is allowed

Item 11: Already known thrombophilic condition
[Sources]: Diagnosis fields
[Keywords]: antithrombin deficiency, protein C/S deficiency, Factor V Leiden, prothrombin G20210A, antiphospholipid syndrome
[Sources] Diagnosis fields
[Exclusion Situations]
1. Normal or negative laboratory results
2. Screening only or suspicion only (such as “to be ruled out,” “possible,” “consider”)
3. No definite diagnostic conclusion
[Decision Criteria] Must satisfy:
A clear diagnosis of one of the above thrombophilic diseases is present in the diagnosis fields
[Decision Rules]
Judge as 1 when the criterion is met
Judge as 0 when it is not met
If not mentioned or evidence is insufficient, judge as 0

[Output Format]

```
{
  "subject_id": "<subject_id>",
  "result": [
    {"item": "Active cancer", "value": 0},
    {"item": "Previous VTE", "value": 0},
    {"item": "Reduced mobility", "value": 0},
    {"item": "Ongoing hormonal treatment", "value": 0},
```

```
    {"item": "Already known thrombophilic condition", "value": 0},
    {"item": "Recent trauma and/or surgery", "value": 0},
    {"item": "Elderly age (≥70 years)", "value": 0},
    {"item": "Heart and/or respiratory failure", "value": 0},
    {"item": "Acute myocardial infarction or ischemic stroke", "value": 0},
    {"item": "Acute infection and/or rheumatologic disorder", "value": 0},
    {"item": "Obesity (BMI ≥30)", "value": 0}
  ],
  "reasoning": [
    {"item": "Active cancer", "reason": "No related diagnosis or treatment mentioned"},
    {"item": "Previous VTE", "reason": "No history of thrombosis mentioned"}
  ]
}
```

[Medical Record Text]
{input_text}

Prompt template for Caprini score extraction

1. Caprini Scoring Basic Prompt

[Task Background]
You are a medical information extraction assistant skilled in identifying venous thromboembolism (VTE)-related risk factors from Chinese electronic medical records.

[Task Objective]
Based on the patient's hospitalization medical record, determine whether the patient meets each item in the Caprini VTE Risk Assessment Model.
This task only determines whether each variable is present; do not assign points, do not calculate a total score, and do not perform risk stratification.

[Input Data]
The input is the patient's complete hospitalization medical record text, which may include: past medical history, history of present illness, diagnoses, physical examination findings, laboratory results, medical orders, surgical records, discharge summary, and so on.

[Decision Rules]
1. If there is an explicit description in the medical record and it meets the definition, label it as 1.
2. If it is not mentioned, is unclear, or cannot be determined, label it as 0.
3. Base judgments only on the original text; do not over-infer from tests, medications, or common knowledge.
4. A justification must be provided for every variable.
5. The justification should quote key information from the original text whenever possible; if the original text does not mention it, explicitly write "Not mentioned".
6. If multiple mutually exclusive items exist within the same category, they must not all be judged as 1; only the single item that best matches the patient's actual condition should be retained as 1, and all others should be marked as 0.

- Age is mutually exclusive: if “Age (41-60 years)”, “Age (60-74 years)”, and “Age (≥75 years)” are all involved, only one item should be retained as 1 according to the actual age, and the others should be 0;

- Bed-rest items are mutually exclusive: if both “Medical patient currently at bed rest” and “Patient confined to bed (>72 hours)” are involved, only the one that best matches the actual duration/status of bed rest should be retained as 1, and the other should be 0;

- Surgery-related items are mutually exclusive: if “Minor surgery planned”, “Laparoscopic surgery (>45 minutes)”, “Major surgery (>45 minutes)”, or “Arthroscopic surgery” are all involved, only the one that best matches the actual surgical situation should be retained as 1, and the others should be 0.

7. All items must be output. No item may be added, removed, or merged.
8. The output must be valid JSON, and nothing other than JSON may appear outside it.
9. Do not output any scoring process, total score, or risk level.

[Caprini Risk Assessment Items]

[1-point items]

- Age (41-60 years)
- Minor surgery planned
- Obesity (BMI>25)
- Swollen legs (current)
- Varicose veins
- Pregnancy or postpartum (<1 month)
- History of unexplained stillborn infant, recurrent spontaneous abortion (≥3), premature birth with toxemia or growth-restricted infant
- Oral contraceptives or hormone replacement therapy
- Sepsis (<1 month)
- Serious lung disease incl.pneumonia (<1 month)
- Abnormal pulmonary function
- Acute myocardial infarction
- Congestive heart failure (<1 month)
- History of inflammatory bowel disease
- Medical patient currently at bed rest

[2-point items]

- Age (60-74 years)
- Arthroscopic surgery
- Major surgery (>45 minutes)
- Laparoscopic surgery (>45 minutes)
- Malignancy (present or previous)
- Patient confined to bed (>72 hours)
- Immobilizing plaster cast (<1 month)
- central venous access

[3-point items]

- Age (≥75 years)
- History of DVT/PE
- Family history of thrombosis
- Positive Factor V Leiden

- Positive Prothrombin 20210A
- Positive lupus anticoagulant
- Elevated anticardiolipin antibodies
- Elevated serum homocysteine
- Heparin-induced thrombocytopenia (HIT)
- Other congenital or acquired thrombophilia

[5-point items]
- Stroke (<1 month)
- Elective major lower extremity arthroplasty
- Hip, pelvis or leg fracture (<1 month)
- Acute spinal cord injury (paralysis) (<1 month)
- Multiple trauma (<1 month)

[Output Format]

Output only one standard JSON object. Do not output any explanations, headings, reasoning process, or any extra text.

The output format is as follows:

{
  "patient_id": "<subject_id>",
  "result": [
    {"item": "Age (41-60 years)", "value": 0},
    {"item": "Minor surgery planned", "value": 0},
    {"item": "Obesity (BMI>25)", "value": 0},
    {"item": "Swollen legs (current)", "value": 0},
    {"item": "Varicose veins", "value": 0},
    {"item": "Pregnancy or postpartum (<1 month)", "value": 0},
    {"item": "History of unexplained stillborn infant, recurrent spontaneous abortion (≥3), premature birth with toxemia or growth-restricted infant", "value": 0},
    {"item": "Oral contraceptives or hormone replacement therapy", "value": 0},
    {"item": "Sepsis (<1 month)", "value": 0},
    {"item": "Serious lung disease incl.pneumonia (<1 month)", "value": 0},
    {"item": "Abnormal pulmonary function", "value": 0},
    {"item": "Acute myocardial infarction", "value": 0},
    {"item": "Congestive heart failure (<1 month)", "value": 0},
    {"item": "History of inflammatory bowel disease", "value": 0},
    {"item": "Medical patient currently at bed rest", "value": 0},
    {"item": "Age (60-74 years)", "value": 0},
    {"item": "Arthroscopic surgery", "value": 0},
    {"item": "Major surgery (>45 minutes)", "value": 0},
    {"item": "Laparoscopic surgery (>45 minutes)", "value": 0},
    {"item": "Malignancy (present or previous)", "value": 0},
    {"item": "Patient confined to bed (>72 hours)", "value": 0},
    {"item": "Immobilizing plaster cast (<1 month)", "value": 0},
    {"item": "central venous access", "value": 0},
    {"item": "Age (≥75 years)", "value": 0},
    {"item": "History of DVT/PE", "value": 0},

{"item": "Family history of thrombosis", "value": 0},
{"item": "Positive Factor V Leiden", "value": 0},
{"item": "Positive Prothrombin 20210A", "value": 0},
{"item": "Positive lupus anticoagulant", "value": 0},
{"item": "Elevated anticardiolipin antibodies", "value": 0},
{"item": "Elevated serum homocysteine", "value": 0},
{"item": "Heparin-induced thrombocytopenia (HIT)", "value": 0},
{"item": "Other congenital or acquired thrombophilia", "value": 0},
{"item": "Stroke (<1 month)", "value": 0},
{"item": "Elective major lower extremity arthroplasty", "value": 0},
{"item": "Hip, pelvis or leg fracture (<1 month)", "value": 0},
{"item": "Acute spinal cord injury (paralysis) (<1 month)", "value": 0},
{"item": "Multiple trauma (<1 month)", "value": 0}
],
"reasoning": [
{"item": "Age (41-60 years)", "reason": "The patient is 30 years old, therefore this is judged as 0"},
{"item": "Minor surgery planned", "reason": "Not mentioned"},
{"item": "Obesity (BMI>25)", "reason": "Not mentioned"},
{"item": "Swollen legs (current)", "reason": "Not mentioned"},
{"item": "Varicose veins", "reason": "Not mentioned"},
{"item": "Pregnancy or postpartum (<1 month)", "reason": "Not mentioned"},
{"item": "History of unexplained stillborn infant, recurrent spontaneous abortion (≥3), premature birth with toxemia or growth-restricted infant", "reason": "Not mentioned"},
{"item": "Oral contraceptives or hormone replacement therapy", "reason": "Not mentioned"},
{"item": "Sepsis (<1 month)", "reason": "Not mentioned"},
{"item": "Serious lung disease incl.pneumonia (<1 month)", "reason": "Not mentioned"},
{"item": "Abnormal pulmonary function", "reason": "Not mentioned"},
{"item": "Acute myocardial infarction", "reason": "Not mentioned"},
{"item": "Congestive heart failure (<1 month)", "reason": "Not mentioned"},
{"item": "History of inflammatory bowel disease", "reason": "Not mentioned"},
{"item": "Medical patient currently at bed rest", "reason": "Not mentioned"},
{"item": "Age (60-74 years)", "reason": "The patient is 30 years old, therefore this is judged as 0"},
{"item": "Arthroscopic surgery", "reason": "Not mentioned"},
{"item": "Major surgery (>45 minutes)", "reason": "Not mentioned"},
{"item": "Laparoscopic surgery (>45 minutes)", "reason": "Not mentioned"},
{"item": "Malignancy (present or previous)", "reason": "The original text does not mention malignancy-related diagnosis, recurrence, metastasis, or antitumor treatment, therefore this is judged as 0"},
{"item": "Patient confined to bed (>72 hours)", "reason": "Not mentioned"},
{"item": "Immobilizing plaster cast (<1 month)", "reason": "Not mentioned"},
{"item": "central venous access", "reason": "Not mentioned"},
{"item": "Age (≥75 years)", "reason": "The patient is 30 years old, therefore this is judged as 0"},
{"item": "History of DVT/PE", "reason": "Not mentioned"},
{"item": "Family history of thrombosis", "reason": "Not mentioned"},

```
    {"item": "Positive Factor V Leiden", "reason": "Not mentioned"},
    {"item": "Positive Prothrombin 20210A", "reason": "Not mentioned"},
    {"item": "Positive lupus anticoagulant", "reason": "Not mentioned"},
    {"item": "Elevated anticardiolipin antibodies", "reason": "Not mentioned"},
    {"item": "Elevated serum homocysteine", "reason": "Not mentioned"},
    {"item": "Heparin-induced thrombocytopenia (HIT)", "reason": "Not mentioned"},
    {"item": "Other congenital or acquired thrombophilia", "reason": "Not mentioned"},
    {"item": "Stroke (<1 month)", "reason": "Not mentioned"},
    {"item": "Elective major lower extremity arthroplasty", "reason": "Not mentioned"},
    {"item": "Hip, pelvis or leg fracture (<1 month)", "reason": "Not mentioned"},
    {"item": "Acute spinal cord injury (paralysis) (<1 month)", "reason": "Not mentioned"},
    {"item": "Multiple trauma (<1 month)", "reason": "Not mentioned"}
  ]
}
[Medical Record Text]
{input_text}
```

2. Caprini Scoring Complex Prompt

[Task Background]

You are a senior clinician familiar with venous thromboembolism (VTE) risk assessment.
Please review the patient's medical record and determine whether the patient meets each risk factor in the Caprini Risk Assessment Model; assign 1 if present and 0 if absent.

[Input Data]

The input is the patient's complete electronic medical record text, which may include:
past medical history, history of present illness, diagnoses, examinations, laboratory tests, medical orders, progress notes, surgical records, discharge records, and so on.

[Task Requirements]

1. Read the medical record text and assess each Caprini risk factor one by one;
2. Assign 1 if the factor is present; assign 0 if it is absent, not mentioned, or cannot be determined;
3. A justification must be provided for every item;
4. Output the result as structured JSON;
5. If multiple items exist within the same category, retain only the one that best matches the patient's condition as 1, and assign 0 to the others; do not assign 1 to multiple mutually exclusive items:
    - Age is mutually exclusive: if "Age (41-60 years)", "Age (60-74 years)", and "Age (≥75 years)" all apply, retain only the one that best matches the actual age;
    - Bed rest is mutually exclusive: if both "Medical patient currently at bed rest" and "Patient confined to bed (>72 hours)" apply, retain only the one that best matches the actual situation;
    - Surgery is mutually exclusive: if "Minor surgery planned", "Laparoscopic surgery (>45 minutes)", "Major surgery (>45 minutes)", or "Arthroscopic surgery" all apply, retain only the one that best matches the actual surgical situation.
6. All items must be output; do not add, delete, or merge any items;
7. The output must be valid JSON, and nothing may appear outside the JSON;
8. Do not calculate a total score and do not determine a risk level.

1-point items
Age (41-60 years)
Medical Definition: Age between 41 and 60 years, inclusive.
Examples:
"Patient is 52 years old" → 1
"Patient is 36 years old" → 0
"Born in 1984, admitted in 2025" → 41 years old → 1

Minor surgery planned
Medical Definition: A minor surgical procedure performed under local or short-duration anesthesia, with operative time <45 minutes, limited trauma, rapid recovery, and few complications.
Examples:
"Skin cyst excision performed" → 1
"Radical gastrectomy for gastric cancer performed" → 0
"Laparoscopic appendectomy (operative time 30 minutes)" → minor surgery → 1

Obesity (BMI>25)
Medical Definition: Body mass index (BMI) greater than 25 kg/m^2.
Examples:
"BMI 28.2 kg/m^2" → 1
"BMI 23.5 kg/m^2" → 0
"Obese body habitus but BMI not measured" → insufficient information → 0

Swollen legs (current)
Medical Definition: Lower-extremity edema or swelling documented in the physical examination or progress notes.
Examples:
"Physical exam: mild bilateral lower-extremity edema" → 1
"No lower-extremity swelling observed" → 0
"Mild lower-extremity swelling" → swelling present → 1

Varicose veins
Medical Definition: Clinically confirmed dilation, tortuosity, or bulging of superficial veins in the lower extremities.
Examples:
"Diagnosis: varicose veins of the lower extremities" → 1
"Denies varicose veins" → 0
"Recurrent varicose veins after surgery" → still present → 1

Pregnancy or postpartum (<1 month)
Medical Definition: Current pregnancy or within 1 month postpartum.
Examples:
"Admitted 2 weeks postpartum" → 1
"History of childbirth, currently not pregnant" → 0

"Admitted at 28 weeks of gestation" → current pregnancy → 1

History of unexplained stillborn infant, recurrent spontaneous abortion (≥3), premature birth with toxemia or growth-restricted infant
Medical Definition: History of unexplained stillbirth, recurrent spontaneous abortion, or premature birth with toxemia or growth-restricted infant.
Examples:
"History of 3 unexplained miscarriages" → 1
"Only 1 induced abortion" → 0
"2 miscarriages of unknown cause" → meets the definition → 1

Oral contraceptives or hormone replacement therapy
Medical Definition: Currently taking estrogen- or progestin-containing medications, including oral contraceptives, hormone replacement therapy, or anti-estrogen therapy.
Examples:
"Long-term oral ethinylestradiol tablets" → 1
"Denies hormone use" → 0
"Dydrogesterone stopped 2 weeks ago" → if used long term → 1

Sepsis (<1 month)
Medical Definition: Sepsis, septicemia, bacteremia, infectious shock, or other systemic inflammatory response syndromes due to infection.
Examples:
"Diagnosis: sepsis" → 1
"Upper respiratory tract infection" → 0
"Severe pneumonia with shock" → considered sepsis → 1

Serious lung disease incl.pneumonia (<1 month)
Medical Definition: COPD, bronchiectasis, pulmonary fibrosis, interstitial lung disease, or pneumonia within 1 month before admission.
Examples:
"Diagnosis: COPD" → 1
"Acute bronchitis" → 0
"Severe pneumonia 2 weeks ago" → 1

Abnormal pulmonary function
Medical Definition: Ventilatory or diffusion dysfunction, or patients requiring mechanical ventilation.
Examples:
"Pulmonary function: obstructive ventilatory dysfunction" → 1
"Normal pulmonary function" → 0
"Requires noninvasive ventilatory support" → abnormal function → 1

Acute myocardial infarction
Medical Definition: Acute myocardial infarction diagnosed during the current hospitalization or recently.
Examples:

“Diagnosis: acute anterior myocardial infarction” → 1
“Old myocardial infarction” → 0

Congestive heart failure (<1 month)
Medical Definition: Significantly impaired cardiac function with clinical signs of heart failure, such as dyspnea or lower-extremity edema.
Examples:
“Diagnosis: congestive heart failure” → 1
“Compensated heart failure” → 0
“Admitted during acute exacerbation” → 1

History of inflammatory bowel disease
Medical Definition: Crohn’s disease or ulcerative colitis.
Examples:
“Active ulcerative colitis” → 1
“Irritable bowel syndrome” → 0
“Crohn’s disease” → counts → 1

Medical patient currently at bed rest
Medical Definition: Bed rest for less than 3 days or mild limitation of activity.
Examples:
“Ambulated on postoperative day 2” → 1
“Bed rest for 5 days” → 0
“Bed rest for 48 hours” → 1

2-point items
Age (60-74 years)
Medical Definition: Age 61–74 years at admission.
Examples:
“Patient is 68 years old” → 1
“58 years old” → 0
“Born in 1960” → 64 years old → 1

Laparoscopic surgery (>45 minutes)
Medical Definition: A laparoscopic procedure performed under general anesthesia with operative duration ≥45 minutes.
Examples:
“Laparoscopic colectomy, 60 minutes” → 1
“Laparoscopic exploration, 20 minutes” → 0
“Laparoscopic cholecystectomy (about 1 hour)” → 1

Major surgery (>45 minutes)
Medical Definition: Highly invasive surgery under general or neuraxial anesthesia involving the thoracic cavity, abdominal cavity, intracranial region, spine, or urinary system, lasting ≥45 minutes.
Examples:
“Radical gastrectomy under general anesthesia” → 1

"Biopsy under local anesthesia" → 0
"Abdominal tumor resection" → major surgery → 1

Arthroscopic surgery
Medical Definition: Arthroscopic surgery for intra-articular repair, debridement, or reconstruction.
Examples:
"Knee arthroscopic debridement performed" → 1
"Planned arthroscopic surgery" → 0
"Arthroscopic repair performed intraoperatively" → 1

Malignancy (present or previous)
Medical Definition: Current malignancy or recently diagnosed malignancy.
Examples:
"Diagnosis: malignant lung tumor" → 1
"Benign thyroid nodule" → 0
"Follow-up after gastric cancer surgery" → if still under treatment → 1

Patient confined to bed (>72 hours)
Medical Definition: Bed rest for 3 days or more, with severe limitation of activity.
Examples:
"Bed rest for 4 days" → 1
"Bed rest for 2 days" → 0
"Mostly bedridden for 4 days, occasional ambulation" → 1

Immobilizing plaster cast (<1 month)
Medical Definition: Recent plaster fixation of a limb or joint.
Examples:
"Right lower limb in plaster cast" → 1
"Plaster mold taken" → 0
"Lower leg plaster fixation for 1 week" → 1
---
central venous access
Medical Definition: Presence of PICC, PORT, central venous catheter, or other deep venous access.
Examples:
"PICC inserted" → 1
"Peripheral venous indwelling needle" → 0
"Subclavian venous catheter placed and retained" → 1

3-point items
Age (≥75 years)
Medical Definition: Age ≥75 years at admission.
Examples:
"78 years old" → 1
"70 years old" → 0
"Born in 1948 (admitted in 2025)" → 77 years old → 1

History of DVT/PE
Medical Definition: Previous confirmed deep vein thrombosis or pulmonary embolism.
Examples:
“Lower-extremity venous thrombosis in 2018” → 1
“Denies history of thrombosis” → 0
“Superficial phlebitis” → not VTE → 0

Family history of thrombosis
Medical Definition: VTE history in a first-degree relative (parents, children, siblings).
Examples:
“Father had pulmonary embolism” → 1
“Aunt had thrombosis” → 0
“Mother had thrombosis of unclear cause” → 1

Positive Factor V Leiden
Medical Definition: Laboratory-confirmed positive Factor V Leiden mutation.
Examples:
“Test: Factor V Leiden positive” → 1
“Negative” → 0
“Previous report indicated carrier status” → 1

Positive Prothrombin 20210A
Medical Definition: Laboratory-confirmed positive prothrombin G20210A gene mutation.
Examples:
“G20210A mutation positive” → 1
“No mutation detected” → 0
“Report: heterozygous mutation” → 1

Positive lupus anticoagulant
Medical Definition: Laboratory evidence of positive lupus anticoagulant (LA).
Examples:
“LA positive” → 1
“LA negative” → 0
“Suspected positive, still positive on repeat test” → 1

Elevated anticardiolipin antibodies
Medical Definition: Positive anticardiolipin antibody (aCL) or antiphospholipid syndrome.
Examples:
“aCL positive” → 1
“Negative” → 0
“Antiphospholipid syndrome” → 1

Elevated serum homocysteine
Medical Definition: Laboratory value above the upper limit of normal, or diagnosis of hyperhomocysteinemia.
Examples:

"Homocysteine 25 μmol/L" → 1
"Normal" → 0
"Diagnosis: hyperhomocysteinemia" → 1

Heparin-induced thrombocytopenia (HIT)
Medical Definition: Confirmed heparin-related immune thrombocytopenia.
Examples:
"Diagnosis: HIT" → 1
"Simple thrombocytopenia" → 0
"Suspected HIT, confirmed by testing" → 1

Other congenital or acquired thrombophilia
Medical Definition: Protein C/S deficiency, antithrombin deficiency, PNH, polycythemia vera, etc.
Examples:
"Antithrombin III deficiency" → 1
"Mild thrombocytosis" → 0
"Reduced protein S activity" → 1

5-point items
Stroke (<1 month)
Medical Definition: Acute cerebral infarction, cerebral hemorrhage, or cerebral embolism.
Examples:
"Admitted for acute cerebral infarction" → 1
"Sequelae of cerebral infarction" → 0
"In recovery phase 1 week after cerebral hemorrhage" → 1

Acute spinal cord injury (paralysis) (<1 month)
Medical Definition: Spinal cord injury or compression within the past 1 month.
Examples:
"Diagnosis: acute spinal cord injury" → 1
"Old injury" → 0
"Spinal cord contusion 1 week ago" → 1

Hip, pelvis or leg fracture (<1 month)
Medical Definition: Hip, pelvic, or lower-extremity fracture occurring within 1 month.
Examples:
"Admitted for femoral neck fracture" → 1
"Upper-extremity fracture" → 0
"Tibia-fibula fracture 2 weeks ago" → 1

Elective major lower extremity arthroplasty
Medical Definition: Hip or knee joint replacement.
Examples:
"Knee joint replacement performed" → 1
"Arthroscopic exploration performed" → 0
"Knee joint replacement 1 year ago" → previous replacement → 0

Multiple trauma (<1 month)
Medical Definition: A multisite traumatic event within 1 month before admission requiring hospitalization or surgery.
Examples:
“Multiple fractures due to a traffic accident” → 1
“Minor skin abrasion” → 0
“One week ago the patient had a traffic accident causing multiple bilateral lower-extremity fractures and splenic rupture” → 1

[Output Format]
Output only one standard JSON object. Do not output any explanations, headings, or extra text.

{
"patient_id": "<subject_id>",
"result": [
{"item": "Age (41-60 years)", "value": 0},
{"item": "Minor surgery planned", "value": 0},
{"item": "Obesity (BMI>25)", "value": 0},
{"item": "Swollen legs (current)", "value": 0},
{"item": "Varicose veins", "value": 0},
{"item": "Pregnancy or postpartum (<1 month)", "value": 0},
{"item": "History of unexplained stillborn infant, recurrent spontaneous abortion (≥3), premature birth with toxemia or growth-restricted infant", "value": 0},
{"item": "Oral contraceptives or hormone replacement therapy", "value": 0},
{"item": "Sepsis (<1 month)", "value": 0},
{"item": "Serious lung disease incl.pneumonia (<1 month)", "value": 0},
{"item": "Abnormal pulmonary function", "value": 0},
{"item": "Acute myocardial infarction", "value": 0},
{"item": "Congestive heart failure (<1 month)", "value": 0},
{"item": "History of inflammatory bowel disease", "value": 0},
{"item": "Medical patient currently at bed rest", "value": 0},
{"item": "Age (60-74 years)", "value": 0},
{"item": "Laparoscopic surgery (>45 minutes)", "value": 0},
{"item": "Major surgery (>45 minutes)", "value": 0},
{"item": "Arthroscopic surgery", "value": 0},
{"item": "Malignancy (present or previous)", "value": 0},
{"item": "Patient confined to bed (>72 hours)", "value": 0},
{"item": "Immobilizing plaster cast (<1 month)", "value": 0},
{"item": "central venous access", "value": 0},
{"item": "Age (≥75 years)", "value": 0},
{"item": "History of DVT/PE", "value": 0},
{"item": "Family history of thrombosis", "value": 0},
{"item": "Positive Factor V Leiden", "value": 0},
{"item": "Positive Prothrombin 20210A", "value": 0},

{"item": "Positive lupus anticoagulant", "value": 0},
{"item": "Elevated anticardiolipin antibodies", "value": 0},
{"item": "Elevated serum homocysteine", "value": 0},
{"item": "Heparin-induced thrombocytopenia (HIT)", "value": 0},
{"item": "Other congenital or acquired thrombophilia", "value": 0},
{"item": "Stroke (<1 month)", "value": 0},
{"item": "Acute spinal cord injury (paralysis) (<1 month)", "value": 0},
{"item": "Hip, pelvis or leg fracture (<1 month)", "value": 0},
{"item": "Elective major lower extremity arthroplasty", "value": 0},
{"item": "Multiple trauma (<1 month)", "value": 0}
],
"reasoning": [
{"item": "Age (41-60 years)", "reason": "The patient is 30 years old, therefore this is judged as 0"},
{"item": "Minor surgery planned", "reason": "Not mentioned"},
{"item": "Obesity (BMI>25)", "reason": "Not mentioned"},
{"item": "Swollen legs (current)", "reason": "Not mentioned"},
{"item": "Varicose veins", "reason": "Not mentioned"},
{"item": "Pregnancy or postpartum (<1 month)", "reason": "Not mentioned"},
{"item": "History of unexplained stillborn infant, recurrent spontaneous abortion (≥3), premature birth with toxemia or growth-restricted infant", "reason": "Not mentioned"},
{"item": "Oral contraceptives or hormone replacement therapy", "reason": "Not mentioned"},
{"item": "Sepsis (<1 month)", "reason": "Not mentioned"},
{"item": "Serious lung disease incl.pneumonia (<1 month)", "reason": "Not mentioned"},
{"item": "Abnormal pulmonary function", "reason": "Not mentioned"},
{"item": "Acute myocardial infarction", "reason": "Not mentioned"},
{"item": "Congestive heart failure (<1 month)", "reason": "Not mentioned"},
{"item": "History of inflammatory bowel disease", "reason": "Not mentioned"},
{"item": "Medical patient currently at bed rest", "reason": "Not mentioned"},
{"item": "Age (60-74 years)", "reason": "The patient is 30 years old, therefore this is judged as 0"},
{"item": "Laparoscopic surgery (>45 minutes)", "reason": "Not mentioned"},
{"item": "Major surgery (>45 minutes)", "reason": "Not mentioned"},
{"item": "Arthroscopic surgery", "reason": "Not mentioned"},
{"item": "Malignancy (present or previous)", "reason": "Not mentioned"},
{"item": "Patient confined to bed (>72 hours)", "reason": "Not mentioned"},
{"item": "Immobilizing plaster cast (<1 month)", "reason": "Not mentioned"},
{"item": "central venous access", "reason": "Not mentioned"},
{"item": "Age (≥75 years)", "reason": "The patient is 30 years old, therefore this is judged as 0"},
{"item": "History of DVT/PE", "reason": "Not mentioned"},
{"item": "Family history of thrombosis", "reason": "Not mentioned"},
{"item": "Positive Factor V Leiden", "reason": "Not mentioned"},
{"item": "Positive Prothrombin 20210A", "reason": "Not mentioned"},
{"item": "Positive lupus anticoagulant", "reason": "Not mentioned"},
{"item": "Elevated anticardiolipin antibodies", "reason": "Not mentioned"},
{"item": "Elevated serum homocysteine", "reason": "Not mentioned"},

```
    {"item": "Heparin-induced thrombocytopenia (HIT)", "reason": "Not mentioned"},
    {"item": "Other congenital or acquired thrombophilia", "reason": "Not mentioned"},
    {"item": "Stroke (<1 month)", "reason": "Not mentioned"},
    {"item": "Acute spinal cord injury (paralysis) (<1 month)", "reason": "Not mentioned"},
    {"item": "Hip, pelvis or leg fracture (<1 month)", "reason": "Not mentioned"},
    {"item": "Elective major lower extremity arthroplasty", "reason": "Not mentioned"},
    {"item": "Multiple trauma (<1 month)", "reason": "Not mentioned"}
  ]
}
```

[Medical Record Text]
{input_text}

3. Caprini Scoring Expert knowledge-augmented Prompt

[Task Objective]

Read the input medical record and determine, item by item, whether the patient meets each item in the Caprini Venous Thromboembolism (VTE) Risk Assessment Model.

Only determine whether each item is present: assign 1 if present and 0 if absent.

Do not calculate a total score and do not determine a risk level.

[Input Data Description]

The input you receive may include the following fields (some may be missing):

- Past medical history, history of present illness, diagnoses, examination results / laboratory data, medical orders, progress notes, marital and reproductive history, personal history, family history, surgical records, discharge records, etc.

[Execution Requirements]

1. Each item must be judged based on keyword matching and logical assessment using the specified source fields.
2. Assign 1 if the condition is met; assign 0 if not met, and explain the reason.
3. For mutually exclusive items, retain only the one that best matches the patient's actual condition as 1, and assign 0 to the others.
4. The output consists of two parts:
   - Reasoning process (item-by-item judgment + quoted original medical record text + explanation)
   - JSON result (fixed fields)
5. If multiple items exist within the same category, retain only the one that best matches the patient's actual condition as 1, and assign 0 to the others; do not assign 1 to multiple mutually exclusive items:
   - Age is mutually exclusive: if "Age (41-60 years)", "Age (60-74 years)", and "Age (≥75 years)" are all involved, retain only the one that best matches the actual age;
   - Bed rest is mutually exclusive: if both "Medical patient currently at bed rest" and "Patient confined to bed (>72 hours)" are involved, retain only the one that best matches the actual situation;
   - Surgery is mutually exclusive: if "Minor surgery planned", "Laparoscopic surgery (>45 minutes)", "Major surgery (>45 minutes)", or "Arthroscopic surgery" are all involved, retain only the one that best matches the actual surgical situation.
6. All items must be output; no item may be added, deleted, or merged.
7. The output must be valid JSON, and nothing may appear outside the JSON.

8. Do not calculate a total score and do not determine a risk level.

Item 1 Age (41-60 years)
[Item Name] Age (41-60 years)
[Source Fields] Date of birth, admission date, age field
[Keywords] “41–60 years”; year of birth range: 1964–1983
[Exclusion Keywords] None
[Logical Conditions]
Condition A: age 41–60 years, or (admission date – date of birth) is between 41 and 60 years, or year of birth is between 1964 and 1983 → assign 1
Condition B: age <41 years, or >60 years, or cannot be determined → assign 0
[Assignment Rule]
This item is mutually exclusive with the other age categories; only one age category may be assigned 1, and the others must be 0.
Priority order:
Direct age field (if present and credible)
Calculated from admission date – date of birth
Estimated from year-of-birth range
[Notes]
If multiple age data are present (for example, the chart states “the patient is 60 years old,” but calculation from the date of birth gives 61 years), use the date-of-birth calculation.
The age must be explicitly recorded or calculated; subjective inference is not allowed.
If the age field conflicts with the year-based calculation, mark the conflict in the reasoning process and explain how the discrepancy was resolved.

Item 2 Age (60-74 years)
[Item Name] Age (60-74 years)
[Source Fields] Date of birth, admission date, age field
[Keywords] “61–74 years”; year of birth range: 1950–1963
[Exclusion Keywords] None
[Logical Conditions]
Condition A: age 61–74 years, or (admission date – date of birth) is between 61 and 74 years, or year of birth is between 1950 and 1963 → assign 1
Condition B: age <61 years, or >74 years, or cannot be determined → assign 0
[Assignment Rule]
This item is mutually exclusive with the other age categories; only one age category may be assigned 1, and the others must be 0.
Priority order:
Direct age field (if present and credible)
Calculated from admission date – date of birth
Estimated from year-of-birth range
[Notes]
If multiple age data are present (for example, the chart states “the patient is 60 years old,” but calculation from the date of birth gives 61 years), use the date-of-birth calculation.
The age must be explicitly recorded or calculated; subjective inference is not allowed.

If the age field conflicts with the year-based calculation, mark the conflict in the reasoning process and explain how the discrepancy was resolved.

Item 3 Age (≥75 years)

[Item Name] Age (≥75 years)

[Source Fields] Date of birth, admission date, age field

[Keywords] “≥75 years”; year of birth range: ≤1949

[Exclusion Keywords] None

[Logical Conditions]

Condition A: age ≥75 years, or (admission date – date of birth) ≥75 years, or year of birth ≤1949 → assign 1

Condition B: age <75 years, or cannot be determined → assign 0

[Assignment Rule]

This item is mutually exclusive with the other age categories; only one age category may be assigned 1, and the others must be 0.

Priority order:

Direct age field (if present and credible)

Calculated from admission date – date of birth

Estimated from year-of-birth range

[Notes]

If multiple age data are present (for example, the chart states “the patient is 74 years old,” but calculation from the date of birth gives 75 years), use the date-of-birth calculation.

The age must be explicitly recorded or calculated; subjective inference is not allowed.

If the age field conflicts with the year-based calculation, mark the conflict in the reasoning process and explain how the discrepancy was resolved.

Item 4 Minor surgery planned

[Item] Minor surgery planned

[Medical Definition] Refers to a procedure performed during the current hospitalization under local anesthesia or short-duration anesthesia, with operative time <45 minutes, limited scope, rapid postoperative recovery, and few complications.

[Source] Surgical records, procedure name, medical record description

[Keywords]

Outpatient surgical procedures: ingrown toenail surgery, local foreign body removal, superficial suturing, puncture

Some endoscopic procedures: colonoscopic single small polyp resection, minor cystoscopic/hysteroscopic resection, diagnostic laparoscopy

Routine gynecologic procedures: cervical biopsy, LEEP, IUD removal, anterior/posterior vaginal wall repair

Simple urologic procedures: ureteral stent insertion/replacement, cystoscopic stone removal

Some breast/thyroid procedures: breast duct exploration, incision and drainage of breast abscess

Some laparoscopic procedures: uncomplicated laparoscopic appendectomy

Superficial procedures: skin/subcutaneous mass excision, cyst excision, superficial lymph node biopsy, breast biopsy, incision and drainage, lipoma excision, granulation tissue curettage

Abscess procedures: incision and drainage of abscess, debridement

Gynecology/urology: cervical conization, dilation and curettage (D&C), tubal ligation, polypectomy

Airway: tracheotomy, tracheostomy
Vascular: arteriovenous fistula formation (AVF), vascular intervention
[Exclusion Keywords]
Thoracotomy/laparotomy/craniotomy, major joint replacement, spinal fixation/fusion, organ resection or transplantation
Operative time or anesthesia time ≥45 minutes
Purely diagnostic endoscopy or endoscopic biopsy (gastroscopy, colonoscopy, bronchoscopy)
Vascular catheterization, thoracic/abdominal drainage tube placement
The four major punctures (thoracentesis / bone marrow aspiration / paracentesis / lumbar puncture / bone marrow biopsy)
[Condition A] Meets any of the above and does not meet the criteria for major surgery → assign 1
[Assignment Rule] If major surgery is met during the current hospitalization, then Minor surgery planned = 0; retain only the item that best matches the patient's actual condition as 1
[Notes] Follow the mutual exclusion rule for surgery-related items. Tracheotomy and arteriovenous fistula surgery are considered minor surgery. ICU admission is often due to critical illness (respiratory failure, infection, shock, etc.); one must not infer major surgery simply because the patient has tracheotomy, closed drainage, or similar procedures.

Item 5 Obesity (BMI>25)
[Item] Obesity (BMI>25)
[Source] Medical record (past medical history / history of present illness), laboratory reports, admission note
[Keywords] BMI, body mass index >25
[Exclusion Keywords] None
[Condition A] BMI or body mass index >25 is explicitly mentioned → assign 1
[Condition B] BMI ≤25 or BMI data are missing → assign 0
[Assignment Rule] If condition is met → 1, otherwise → 0
[Notes] Missing values or BMI ≤25 do not count

Item 6 Swollen legs (current)
[Item] Swollen legs (current)
[Source] Physical examination, exam findings, history of present illness
[Keywords] lower-extremity swelling, lower-extremity edema, lower-extremity puffiness, mild lower-extremity edema, bilateral lower-extremity edema
[Exclusion Keywords] no, none, not seen
[Condition A] The source contains a keyword and does not contain any exclusion term → assign 1
[Assignment Rule] If condition is met → 1, otherwise → 0
[Notes] Must be a positive description

Item 7 Varicose veins
[Item] Varicose veins
[Source] Physical examination, exam findings, history of present illness, diagnosis
[Keywords] varicose veins
[Exclusion Keywords] no, none, not seen
[Condition A] The source contains the keyword and does not contain any exclusion term → assign 1
[Assignment Rule] If condition is met → 1, otherwise → 0

[Notes] Must be a positive description

Item 8　Pregnancy or postpartum (<1 month)
[Item] Pregnancy or postpartum (<1 month)
[Source] Past medical history, marital and reproductive history
[Keywords] current pregnancy, pregnant, delivery, postpartum
[Exclusion Keywords] not pregnant, no delivery, non-pregnant
[Condition A] Currently pregnant → assign 1
[Condition B] Delivery ≤30 days (within 1 month postpartum) → assign 1
[Assignment Rule] If condition is met → 1, otherwise → 0
[Notes] If delivery is mentioned but no time is specified and there is no exact date → 0; negative descriptions → 0

Item 9　History of unexplained stillborn infant, recurrent spontaneous abortion (≥3), premature birth with toxemia or growth-restricted infant
[Item] History of unexplained stillborn infant, recurrent spontaneous abortion (≥3), premature birth with toxemia or growth-restricted infant
[Source] Past medical history, history of present illness
[Keywords] unexplained miscarriage, recurrent miscarriage
[Exclusion Keywords] no miscarriage history, no recurrent miscarriage, no miscarriage seen
[Condition A] The source explicitly mentions unexplained miscarriage or recurrent miscarriage → assign 1
[Assignment Rule] If condition is met → 1, otherwise → 0
[Notes] If only “miscarriage” is described without qualifying wording, do not count it

---

Item 10　Oral contraceptives or hormone replacement therapy
[Item] Oral contraceptives or hormone replacement therapy
[Source] Past medical history, medical orders
[Keywords] estrogen, progestin, estradiol, ethinyl estradiol, estradiol valerate, Progynova, dydrogesterone, progesterone, raloxifene, tamoxifen, anastrozole, letrozole
[Exclusion Keywords] not used, discontinued, not taking, no hormone use
[Condition A] The source contains any keyword and does not contain any exclusion term → assign 1
[Assignment Rule] If condition is met → 1, otherwise → 0
[Notes] Any dosage form counts

Item 11　Sepsis (<1 month)
[Item] Sepsis (<1 month)
[Medical Definition] Sepsis refers to sepsis or infection-related shock accompanied by organ dysfunction or circulatory dysfunction.
[Mandatory Keywords] sepsis, septicemia, bacteremia, toxemia, infectious shock, septic shock, severe pneumonia, septic shock
[Exclusion Keywords]
chronic (for example, “chronic pulmonary infection”)
Helicobacter pylori infection
“no infection,” “denies infection,” “infection not seen”
mild general infections (such as ordinary pneumonia, urinary tract infection, upper respiratory infection,

simple urinary tract infection, etc.)
[Conditions]
Condition A: diagnosis field contains a keyword (and no exclusion term), and the medical orders include anti-infective treatment (antibiotics / antifungals / antivirals) → assign 1
Condition B: past medical history explicitly mentions a sepsis-related disease within 1 month (keyword present), with no exclusion term → assign 1
Otherwise: no keyword appears, or only "fever/infection + antibiotics + elevated inflammatory markers" is present → assign 0
[Mandatory Rule]
Scoring is allowed only when one of the keywords (sepsis, septicemia, bacteremia, toxemia, infectious shock, septic shock, severe pneumonia) explicitly appears in the diagnosis/record.
If none of these keywords appears, assign 0 in all cases, even if the patient has signs of infection, elevated inflammatory markers, or receives antibiotics.
[Notes]
If infection in past medical history occurred more than 1 month ago → do not assign.
If "sepsis" appears in the diagnosis but anti-infective drugs are absent from the orders, still judge as 1 (to avoid missing cases due to incomplete orders), and explain this in the reasoning process.
Inference is prohibited; keywords must match exactly. If the text does not contain any of the keywords [sepsis, septicemia, bacteremia, toxemia, infectious shock, septic shock, severe pneumonia], then even if fever, infectious manifestations, antibiotic treatment, or elevated inflammatory markers are present, it must be judged as 0.

Item 12 Serious lung disease incl.pneumonia (<1 month)
[Item] Serious lung disease incl.pneumonia (<1 month)
[Medical Definition] Serious lung disease refers to structural lung diseases that cause significant pulmonary dysfunction or increase thrombosis risk, including chronic obstructive pulmonary disease (COPD), bronchiectasis, pulmonary fibrosis, other structural lung diseases, and pneumonia occurring within 1 month before admission.
[Source] Past medical history, history of present illness, diagnosis fields
[Keywords] chronic obstructive pulmonary disease, COPD, bronchiectasis, pulmonary fibrosis, interstitial lung disease, interstitial pulmonary fibrosis, structural lung disease, cystic fibrosis, destroyed lung
Severe pneumonia, pneumonia (if there is a date or wording such as "recent" indicating <1 month)
[Exclusion Keywords]
asthma (asthma alone does not count), mild pneumonia (if no severe wording or no "<1 month" indication, it does not count)
pulmonary hypertension (a separate disease, not included in this item), upper respiratory infection, acute bronchitis, etc.
[Condition] The source contains one of the above lung disease keywords and does not contain exclusion terms; if "pneumonia" is present, it must be clearly within ≤1 month (for example, "pneumonia 2 weeks ago," "recent pneumonia"), otherwise do not assign.
[Assignment Rule] Meets condition → Serious lung disease incl.pneumonia (<1 month) = 1, otherwise = 0
[Notes]
"Serious lung disease" includes both chronic structural lung disease and recent pneumonia (<1 month); prior pneumonia (>1 month) does not count.

Item 13 Abnormal pulmonary function
[Item] Abnormal pulmonary function
[Source] History of present illness, diagnosis, ancillary tests, treatment / medical orders
[Keywords]
Disease diagnosis terms: chronic obstructive pulmonary disease (COPD), asthma, interstitial pneumonia / interstitial lung disease, pulmonary fibrosis, pneumoconiosis, occupational lung disease, bronchiectasis, respiratory failure
Treatment terms: mechanical ventilation, noninvasive ventilation, endotracheal intubation, ventilator-assisted ventilation, pulmonary rehabilitation
Functional abnormality terms: obstructive / restrictive / mixed ventilatory dysfunction, small airway dysfunction
Pulmonary function index terms: reduced FEV1/FVC, reduced FEV1, reduced FVC, reduced DLCO / diffusion capacity, airway obstruction
Test result terms: positive bronchodilator test, positive bronchial provocation test
[Exclusion Keywords]
normal pulmonary function, normal ventilation, normal diffusion function, negative bronchodilator test, no airflow limitation
[Conditions]
Condition A: diagnosis, functional index, or test result keywords are present → assign 1 (if no exclusion term)
Condition B: treatment keywords (mechanical ventilation, noninvasive ventilation, ventilator-assisted ventilation, endotracheal intubation, etc.) indicate existing dysfunction → assign 1 (if no exclusion term)
[Assignment Rule]
Meets Condition A or Condition B → 1, otherwise → 0

Item 14 Acute myocardial infarction
[Item] Acute myocardial infarction
[Source] Diagnosis
[Keywords] acute myocardial infarction, acute XXX myocardial infarction (such as anterior, inferior, posterior wall, right ventricle, etc.)
[Exclusion Keywords] old myocardial infarction
[Condition A] Diagnosis contains any keyword and does not contain exclusion terms → assign 1
[Assignment Rule] Meets condition → 1, otherwise → 0
[Notes] Keyword matching is case-insensitive and not affected by full-width / half-width forms

---

Item 15 Congestive heart failure (<1 month)
[Item] Congestive heart failure (<1 month)
[Source] Diagnosis
[Keywords] congestive heart failure, CHF, heart failure, cardiac insufficiency, acute pulmonary edema, cardiogenic pulmonary edema, NYHA class III, NYHA class IV, chronic heart failure
[Exclusion Keywords] ischemic, compensated stage, normal LVEF, normal cardiac function, NYHA class I, NYHA class II
[Condition A] Diagnosis contains any keyword and does not contain exclusion terms → assign 1
[Assignment Rule] Meets condition → 1, otherwise → 0

Item 16 History of inflammatory bowel disease

[Item] History of inflammatory bowel disease

[Source] Past medical history, history of present illness, diagnosis

[Keywords] Crohn's disease, ulcerative colitis

[Exclusion Keywords] none

[Condition A] Diagnosis or history of present illness contains any keyword → assign 1

[Condition B] Past medical history contains any keyword and the same record contains a time cue ≤30 days (such as "within 1 month," "past 2 weeks," "5 days before admission," etc.) → assign 1

[Assignment Rule] Meets Condition A or B → 1, otherwise → 0

Item 17 Medical patient currently at bed rest

[Item] Medical patient currently at bed rest

[Source] Physical examination, nursing records, medical orders, progress notes

[Keywords] bed rest for 2 days, bed rest for 48 hours, bed rest <3 days, immobilization <72 hours, crutch walking, wheelchair use, walking distance <10 meters, requires assistance walking, only short-distance activity, ambulated on day 2 after admission/surgery, activity resumed within 48 hours after onset

[Exclusion Keywords] prolonged bed rest, bed rest >72 hours, activity restriction >3 days

[Condition A] Source contains a clear time description <72 hours → assign 1

[Condition B] Functional limitation indicates short-term bed rest definition → assign 1

[Condition C] Progress notes show the patient got out of bed within 48 hours after admission/surgery → assign 1

[Assignment Rule] Meets any condition and does not contain exclusion terms → 1, otherwise → 0

[Notes] If the record only says "bed rest" without any time information, do not count; mutually exclusive with "Patient confined to bed (>72 hours)"

Item 18 Arthroscopic surgery

[Item] Arthroscopic surgery

[Source] Surgical records, diagnosis, medical orders

[Keywords] arthroscopic surgery, knee arthroscopy, hip arthroscopy, shoulder arthroscopy, wrist arthroscopy, ankle arthroscopy, arthroscopy + debridement/repair/reconstruction

[Exclusion Keywords] planned arthroscopic surgery not performed

[Condition A] Source contains any keyword → assign 1

[Assignment Rule] If performed during the current hospitalization → 1, otherwise → 0

[Notes] If the criteria for Major surgery (>45 minutes) or Minor surgery planned are met, then Arthroscopic surgery = 0 (mutually exclusive)

Item 19 Major surgery (>45 minutes)

[Item] Major surgery (>45 minutes)

[Medical Definition] Refers to a major open surgery performed during the current hospitalization under general anesthesia or neuraxial anesthesia, with operative time (including anesthesia time) ≥45 minutes, involving the thoracic cavity, abdominal cavity, intracranial region, spine, major joints, or the genitourinary system. Such surgeries are highly traumatic and usually followed by prolonged bed rest.

[Source] Surgical records, procedure name

[Keywords] Operative time must be ≥45 minutes and performed under general anesthesia. Coronary

artery bypass grafting, heart valve replacement, thoracic aortic aneurysm repair, open lobectomy, pneumonectomy, thymoma resection, radical gastrectomy (total / distal), radical colorectal cancer surgery (right / left / total colectomy), esophagectomy, pancreaticoduodenectomy (Whipple), extensive small/large bowel resection, hepatectomy / liver cancer resection, abdominal tumor resection, open cholecystectomy (with bile duct exploration), craniotomy, intracranial tumor resection, spinal decompression/fusion, decompression for traumatic brain injury, clipping of intracranial aneurysm, transabdominal radical prostatectomy, partial / radical nephrectomy, cystectomy with urinary diversion, ureterectomy, radical cystectomy, pelvic exenteration, spinal canal tumor resection, aneurysm clipping / vascular reconstruction, extensive hysterectomy, total hysterectomy (abdominal / vaginal / laparoscopic), ovarian tumor resection, myomectomy, radical hysterectomy for cervical cancer, cesarean section, open reduction and internal fixation of fractures, pelvic fracture surgery, spinal fusion, organ transplantation, modified radical mastectomy, extensive burns

[Exclusion Keywords] interventional or minimally invasive surgeries are classified as minor surgery; catheter placement procedures

[Condition A] Source matches any of the above keywords → assign 1

[Assignment Rule] Meets condition → 1, otherwise → 0

[Notes] Mutually exclusive with Minor surgery planned, Arthroscopic surgery, and Laparoscopic surgery (>45 minutes). "Major surgery" must satisfy surgery type + traumatic extent + time ≥45 minutes. Do not misclassify ICU admission or procedures such as closed drainage, tracheotomy, PICC, or AVF formation as major surgery. Pure diagnosis/biopsy/minor endoscopic resection is not major surgery. Limited to the current hospitalization.

Item 20 Laparoscopic surgery (>45 minutes)

[Item] Laparoscopic surgery (>45 minutes)

[Source] Surgical records, procedure name

[Keywords] laparoscopy + operative duration ≥45 minutes; laparoscopic radical gastrectomy, laparoscopic colectomy, laparoscopic rectal cancer surgery (TME), laparoscopic partial small bowel resection, laparoscopic cholecystectomy, laparoscopic partial hepatectomy, laparoscopic distal pancreatectomy, laparoscopic nephrectomy (partial / total), laparoscopic adrenalectomy, laparoscopic radical prostatectomy, single-port laparoscopic appendectomy, laparoscopic inguinal hernia repair, laparoscopic ovarian cystectomy (small cysts)

[Exclusion Keywords] laparoscopic exploration only (without resection / repair / reconstruction)

[Condition A] Source matches any of the above keywords → assign 1

[Assignment Rule] Meets condition → 1, otherwise → 0

[Notes] Mutually exclusive with Major surgery (>45 minutes), Minor surgery planned, and Arthroscopic surgery; limited to the current hospitalization.

Item 21 Malignancy (present or previous)

[Item] Malignancy (present or previous)

[Source] Diagnosis

[Keywords] cancer, malignant tumor, leukemia, lymphoma, multiple myeloma, malignant XXX effusion, chemotherapy, chemical treatment, immunotherapy, targeted therapy, radiotherapy, radiation therapy

[Exclusion Keywords] benign tumor, benign nodule

[Condition A] Diagnosis contains any keyword and no exclusion term → assign 1

[Assignment Rule] Meets condition → 1, otherwise → 0

Item 22 Patient confined to bed (>72 hours)

[Item] Patient confined to bed (>72 hours)

[Source] Medical orders, past medical history, diagnosis, progress notes, admitting department, operations, surgical records

[Keywords] absolute bed rest, strict bed rest, bed rest, not allowed out of bed, temporarily no ambulation, requires bed rest treatment, bed-rest rehabilitation, bed-rest recovery, in-bed activities, only allowed to sit up at bedside, activity restriction, immobilization >3 days, hemiplegia, postoperative orthopedic immobilization, postoperative bed rest observation, bed rest for multiple trauma, supine position for 6 weeks after spine surgery, continuously bedridden without walking, fourth day of bed rest, already bedridden >72 hours, activity ability: bedridden, Braden score indicating long-term bed-rest risk, Morse fall risk score leading to activity restriction, admission to ICU/CCU/EICU/RICU/intensive care unit, or undergoing endotracheal intubation, laparotomy, pelvic surgery, cesarean section, organ transplantation, craniotomy, thoracotomy, abdominal surgery, spinal surgery, etc.

[Exclusion Keywords] bed rest <72 hours, activity status unclear, already out of bed, walking at bedside, ambulation, crutch walking, moves freely, active freely, started rehabilitation training

[Condition A] Source matches any keyword and contains no exclusion term → assign 1

[Assignment Rule] Meets condition → 1, otherwise → 0

[Notes] Mutually exclusive with “Medical patient currently at bed rest”

Item 23 Immobilizing plaster cast (<1 month)

[Item] Immobilizing plaster cast (<1 month)

[Source] Surgical records, medical orders

[Keywords] plaster cast

[Exclusion Keywords] plaster allergy test, plaster mold taking (non-therapeutic use)

[Condition A] Source contains “plaster cast” and no exclusion term → assign 1

[Assignment Rule] Meets condition → 1, otherwise → 0

Item 24 central venous access

[Item] central venous access

[Source] Medical orders

[Keywords] PICC, venous catheter, infusion port, PORT, venous access, central venous catheter, central venous catheterization, central venous access, internal jugular catheterization, subclavian vein catheterization, femoral vein catheterization, deep venous catheterization

[Exclusion Keywords] peripheral venous indwelling needle, superficial venous puncture, isolated peripheral arterial catheterization

[Condition A] Source contains any keyword and no exclusion term → assign 1

[Assignment Rule] Meets condition → 1, otherwise → 0

Item 25 History of DVT/PE

[Item] History of DVT/PE

[Source] Past medical history

[Keywords] VTE, DVT, PE, venous thrombosis, pulmonary embolism, pulmonary artery thrombosis

[Exclusion Keywords] denies, none, great saphenous vein, small saphenous vein, internal pudendal vein, anterior abdominal wall vein, external pudendal vein, superficial circumflex iliac vein, external jugular vein, cephalic vein, basilic vein, median cubital vein, thrombophlebitis

[Condition A] Source contains any keyword and no exclusion term → assign 1
[Assignment Rule] Meets condition → 1, otherwise → 0
[Notes] Must be documented in past medical history, not an initial diagnosis during the current hospitalization
---
Item 26 Family history of thrombosis
[Item] Family history of thrombosis
[Source] Family history
[Keywords] father, mother, children, brothers, sisters + deep vein thrombosis, pulmonary embolism
[Exclusion Keywords] denies, none
[Condition A] A first-degree relative is confirmed to have DVT or PE and there is no exclusion term → assign 1
[Assignment Rule] Meets condition → 1, otherwise → 0
[Notes] Must be a first-degree relative; second-degree or more distant relatives do not count
---
Item 27 Positive Factor V Leiden
[Item] Positive Factor V Leiden
[Source] History of present illness, past medical history, family history, diagnosis
[Keywords] Factor V Leiden mutation, V Leiden mutation, Factor V Leiden
[Exclusion Keywords] denies, not found
[Condition A] Source contains any keyword and no exclusion term → assign 1
[Assignment Rule] Meets condition → 1, otherwise → 0

Item 28 Positive Prothrombin 20210A
[Item] Positive Prothrombin 20210A
[Source] History of present illness, past medical history, family history, diagnosis
[Keywords] prothrombin G20210A mutation, G20210A mutation, prothrombin mutation
[Exclusion Keywords] denies, not found
[Condition A] Source contains any keyword and no exclusion term → assign 1
[Assignment Rule] Meets condition → 1, otherwise → 0

Item 29 Positive lupus anticoagulant
[Item] Positive lupus anticoagulant
[Source] Laboratory data, diagnosis, past medical history
[Keywords] positive lupus anticoagulant, LA positive, LA(+)
[Exclusion Keywords] not seen, negative, (-), suspected positive, pending recheck, repeat test recommended, denies, none
[Condition A] Laboratory data contain any positive keyword and no exclusion term → assign 1
[Condition B] Diagnosis or past medical history contain any positive keyword and no exclusion term → assign 1
[Assignment Rule] Meets Condition A or B → 1, otherwise → 0

Item 30 Elevated serum homocysteine
[Item] Elevated serum homocysteine
[Source] Laboratory data, history of present illness, past medical history, diagnosis

[Keywords] elevated homocysteine, high homocysteine, hyperhomocysteinemia
[Exclusion Keywords] denies, none, not seen
[Condition A] The laboratory test name contains “homocysteine” and the value is above the upper limit of the reference range → assign 1
[Condition B] The source text must contain an abnormal keyword (elevated, high, hyperhomocysteinemia), and must not contain exclusion terms (denies, none, not seen, negative, measurement, examination, test) → assign 1
[Assignment Rule] Meets Condition A or B → 1, otherwise → 0

Item 31 Heparin-induced thrombocytopenia (HIT)
[Item] Heparin-induced thrombocytopenia (HIT)
[Source] History of present illness, past medical history, diagnosis
[Keywords] heparin-induced thrombocytopenia, heparin-related thrombocytopenia, HIT, Heparin-Induced Thrombocytopenia
[Exclusion Keywords] denies, none, not seen
[Condition A] Source contains any keyword and no exclusion term → assign 1
[Assignment Rule] Meets condition → 1, otherwise → 0
[Notes] Must be a confirmed diagnosis, not merely suspected or under evaluation

---

Item 32 Other congenital or acquired thrombophilia
[Item] Other congenital or acquired thrombophilia
[Source] Past medical history, EDC-other risk factors
[Keywords] antithrombin III deficiency, chronic myeloid leukemia, polycythemia vera, essential thrombocythemia, primary myelofibrosis, chronic neutrophilic leukemia, antithrombin deficiency, protein C deficiency, protein S deficiency, paroxysmal nocturnal hemoglobinuria, PNH, dysfibrinogenemia, homozygous methylenetetrahydrofolate reductase gene mutation, MTHFR mutation
[Exclusion Keywords] denies, none, not seen
[Condition A] Source contains any keyword and no exclusion term → assign 1
[Assignment Rule] Meets condition → 1, otherwise → 0
[Notes] Must be a confirmed diagnosis

Item 33 Stroke (<1 month)
[Item] Stroke (<1 month)
[Source] Past medical history, diagnosis
[Keywords] cerebral infarction, cerebral thrombosis, cerebral embolism, hypertensive intracerebral hemorrhage, intracranial hemorrhage, cerebral hemorrhage, lobar hemorrhage, brainstem hemorrhage, cerebellar hemorrhage, intraventricular hemorrhage, secondary intracerebral hemorrhage (AVM, aneurysm, tumor stroke, anticoagulation-related, etc.)
[Exclusion Keywords] denies, none, not seen, old, subarachnoid hemorrhage, lacunar cerebral infarction, transient ischemic attack, TIA, sequelae of cerebral infarction, sequelae of cerebral hemorrhage
[Condition A] Past medical history contains a time cue ≤30 days + any keyword → assign 1
[Condition B] Diagnosis contains any keyword and no exclusion term → assign 1
[Assignment Rule] Meets Condition A or B → 1, otherwise → 0

Item 34 Elective major lower extremity arthroplasty

[Item] Elective major lower extremity arthroplasty
[Source] Past medical history, surgical records, procedure name
[Keywords] hip replacement surgery, knee replacement surgery
[Exclusion Keywords] denies, none, not seen
[Condition A] Past medical history contains a time cue ≤30 days + any keyword → assign 1
[Condition B] Surgical record or procedure name contains any keyword → assign 1
[Assignment Rule] Meets Condition A or B → 1, otherwise → 0
[Notes] Does not include arthroscopic surgery or partial joint resurfacing
---
Item 35 Hip, pelvis or leg fracture (<1 month)
[Item] Hip, pelvis or leg fracture (<1 month)
[Source] Diagnosis, medical record notes
[Time Window] Recent fracture occurring or clearly documented during the current hospitalization or within ≤30 days before admission.
[Keywords] femoral neck fracture, intertrochanteric fracture, subtrochanteric fracture, femoral head fracture, iliac wing fracture, pubic ramus fracture, sacral fracture, pubic symphysis separation, unstable pelvic ring fracture, acetabular fracture, femoral shaft fracture, distal femur fracture, patellar fracture, tibial plateau fracture, tibia-fibula shaft fracture, ankle fracture, metatarsal fracture, Jones fracture, March fracture, toe fracture, calcaneal fracture, talus fracture
[Exclusion Keywords] denies, none, not seen
[Condition A] Contains a time cue ≤30 days + any keyword in the source and no exclusion term → assign 1
[Assignment Rule] Meets condition → 1, otherwise → 0
[Notes]
Only hip, pelvis, or lower-extremity fractures count; upper-extremity fractures do not.
Fractures older than 30 days or old fractures do not count.

Item 36 Acute spinal cord injury (paralysis) (<1 month)
[Item] Acute spinal cord injury (paralysis) (<1 month)
[Source] Diagnosis, past medical history
[Keywords] cervical spinal cord injury, thoracic spinal cord injury, lumbar spinal cord injury, sacral spinal cord injury, spinal cord compression, spinal shock, spinal cord contusion, spinal cord edema, spinal cord hemorrhage, spinal cord ischemia, spinal cord impingement, spinal cord embolism, syringomyelia, diastematomyelia, complete spinal cord injury, incomplete spinal cord injury, spinal concussion, spinal cord functional injury, spinal cord hematoma
[Exclusion Keywords] no paralysis, recovered, old, stable stage
[Condition A] Diagnosis contains any keyword and no exclusion term → assign 1
[Condition B] Past medical history contains a time cue ≤30 days + any keyword and no exclusion term → assign 1
[Assignment Rule] Meets Condition A or B → 1, otherwise → 0
[Notes] Must be a confirmed diagnosis, not merely suspected

Item 37 Elevated anticardiolipin antibodies
[Item] Elevated anticardiolipin antibodies
[Source] Ancillary tests, diagnosis
[Keywords]

Laboratory data - aCL / anticardiolipin antibody - “positive / above upper reference limit”
Diagnosis contains antiphospholipid antibody syndrome, antiphospholipid syndrome
[Exclusion] Exclude “negative / (-) / not detected / weakly positive / suspected positive / repeat test recommended / transiently positive”
[Condition A] Ancillary tests or diagnosis meet the above keywords and do not contain exclusion terms → assign 1
[Assignment Rule] Meets condition → 1, otherwise → 0

Item 38 Multiple trauma (<1 month)
[Item] Multiple trauma (<1 month)
[Source] Past medical history, history of present illness, diagnosis, medical record notes
[Medical Definition] Refers to a severe traumatic event occurring within 1 month before admission, usually involving multiple injuries or major organ injury, and requiring hospitalization, surgery, or intensive monitoring.
[Keywords] car accident, traffic accident, multiple trauma, multiple fractures, complex trauma, fall from height, crush injury, severe trauma, traumatic shock, splenic rupture, liver rupture, renal contusion, pelvic fracture with multiple injuries, combined thoracoabdominal injury, multiple soft tissue lacerations/contusions
[Exclusion Keywords] minor skin trauma, minor abrasion, superficial injury, old injury, previous trauma, no trauma, denies trauma
[Condition A] The source contains a time cue ≤30 days, contains any major trauma-related keyword, and does not contain any exclusion term → assign 1
[Assignment Rule] Meets condition → 1, otherwise → 0
[Notes]
1. The trauma must have occurred within the past 1 month;
2. It must be major trauma; minor trauma does not count;
3. If only “trauma” is mentioned without severity or time information, do not assign;
4. If multiple trauma, complex trauma, major organ injury, or traumatic shock is explicitly documented, it may be directly judged as major trauma.

[Reasoning Process]
List the judgment result for each item, including:
- Item name
- Judgment result
- Reason (quote the original medical record text + explain the judgment)

[Output JSON]
[Output Format]
Output only one standard JSON object. Do not output any explanations, headings, or extra text.

```
{
  "patient_id": "<subject_id>",
  "result": [
    {"item": "Age (41-60 years)", "value": 0},
    {"item": "Minor surgery planned", "value": 0},
    {"item": "Obesity (BMI>25)", "value": 0},
```

{"item": "Swollen legs (current)", "value": 0},
{"item": "Varicose veins", "value": 0},
{"item": "Pregnancy or postpartum (<1 month)", "value": 0},
{"item": "History of unexplained stillborn infant, recurrent spontaneous abortion (≥3), premature birth with toxemia or growth-restricted infant", "value": 0},
{"item": "Oral contraceptives or hormone replacement therapy", "value": 0},
{"item": "Sepsis (<1 month)", "value": 0},
{"item": "Serious lung disease incl.pneumonia (<1 month)", "value": 0},
{"item": "Abnormal pulmonary function", "value": 0},
{"item": "Acute myocardial infarction", "value": 0},
{"item": "Congestive heart failure (<1 month)", "value": 0},
{"item": "History of inflammatory bowel disease", "value": 0},
{"item": "Medical patient currently at bed rest", "value": 0},
{"item": "Age (60-74 years)", "value": 0},
{"item": "Laparoscopic surgery (>45 minutes)", "value": 0},
{"item": "Major surgery (>45 minutes)", "value": 0},
{"item": "Arthroscopic surgery", "value": 0},
{"item": "Malignancy (present or previous)", "value": 0},
{"item": "Patient confined to bed (>72 hours)", "value": 0},
{"item": "Immobilizing plaster cast (<1 month)", "value": 0},
{"item": "central venous access", "value": 0},
{"item": "Age (≥75 years)", "value": 0},
{"item": "History of DVT/PE", "value": 0},
{"item": "Family history of thrombosis", "value": 0},
{"item": "Positive Factor V Leiden", "value": 0},
{"item": "Positive Prothrombin 20210A", "value": 0},
{"item": "Positive lupus anticoagulant", "value": 0},
{"item": "Elevated anticardiolipin antibodies", "value": 0},
{"item": "Elevated serum homocysteine", "value": 0},
{"item": "Heparin-induced thrombocytopenia (HIT)", "value": 0},
{"item": "Other congenital or acquired thrombophilia", "value": 0},
{"item": "Stroke (<1 month)", "value": 0},
{"item": "Acute spinal cord injury (paralysis) (<1 month)", "value": 0},
{"item": "Hip, pelvis or leg fracture (<1 month)", "value": 0},
{"item": "Elective major lower extremity arthroplasty", "value": 0},
{"item": "Multiple trauma (<1 month)", "value": 0}
],
"reasoning": [
{"item": "Age (41-60 years)", "reason": "The patient is 30 years old, therefore this is judged as 0"},
{"item": "Minor surgery planned", "reason": "Not mentioned"},
{"item": "Obesity (BMI>25)", "reason": "Not mentioned"},
{"item": "Swollen legs (current)", "reason": "Not mentioned"},
{"item": "Varicose veins", "reason": "Not mentioned"},
{"item": "Pregnancy or postpartum (<1 month)", "reason": "Not mentioned"},
{"item": "History of unexplained stillborn infant, recurrent spontaneous abortion (≥3), premature

birth with toxemia or growth-restricted infant", "reason": "Not mentioned"},
{"item": "Oral contraceptives or hormone replacement therapy", "reason": "Not mentioned"},
{"item": "Sepsis (<1 month)", "reason": "Not mentioned"},
{"item": "Serious lung disease incl.pneumonia (<1 month)", "reason": "Not mentioned"},
{"item": "Abnormal pulmonary function", "reason": "Not mentioned"},
{"item": "Acute myocardial infarction", "reason": "Not mentioned"},
{"item": "Congestive heart failure (<1 month)", "reason": "Not mentioned"},
{"item": "History of inflammatory bowel disease", "reason": "Not mentioned"},
{"item": "Medical patient currently at bed rest", "reason": "Not mentioned"},
{"item": "Age (60-74 years)", "reason": "The patient is 30 years old, therefore this is judged as 0"},
{"item": "Laparoscopic surgery (>45 minutes)", "reason": "Not mentioned"},
{"item": "Major surgery (>45 minutes)", "reason": "Not mentioned"},
{"item": "Arthroscopic surgery", "reason": "Not mentioned"},
{"item": "Malignancy (present or previous)", "reason": "Not mentioned"},
{"item": "Patient confined to bed (>72 hours)", "reason": "Not mentioned"},
{"item": "Immobilizing plaster cast (<1 month)", "reason": "Not mentioned"},
{"item": "central venous access", "reason": "Not mentioned"},
{"item": "Age (≥75 years)", "reason": "The patient is 30 years old, therefore this is judged as 0"},
{"item": "History of DVT/PE", "reason": "Not mentioned"},
{"item": "Family history of thrombosis", "reason": "Not mentioned"},
{"item": "Positive Factor V Leiden", "reason": "Not mentioned"},
{"item": "Positive Prothrombin 20210A", "reason": "Not mentioned"},
{"item": "Positive lupus anticoagulant", "reason": "Not mentioned"},
{"item": "Elevated anticardiolipin antibodies", "reason": "Not mentioned"},
{"item": "Elevated serum homocysteine", "reason": "Not mentioned"},
{"item": "Heparin-induced thrombocytopenia (HIT)", "reason": "Not mentioned"},
{"item": "Other congenital or acquired thrombophilia", "reason": "Not mentioned"},
{"item": "Stroke (<1 month)", "reason": "Not mentioned"},
{"item": "Acute spinal cord injury (paralysis) (<1 month)", "reason": "Not mentioned"},
{"item": "Hip, pelvis or leg fracture (<1 month)", "reason": "Not mentioned"},
{"item": "Elective major lower extremity arthroplasty", "reason": "Not mentioned"},
{"item": "Multiple trauma (<1 month)", "reason": "Not mentioned"}
]
}

[Medical Record Text]
{input_text}

Supplementary Collaborating Centers

Collaborating centers include: Beijing Fengtai Youanmen Hospital, Beijing, China (Jie Ren); The Affiliated Hospital of Putian University, Putian, China (Xiaoyu Qi); The Affiliated Union Hospital of Fujian Medical University, Fuzhou, China (Yong Wu); Guilin People's Hospital, Guilin, China (Yan Zuo); The First Affiliated Hospital of Henan University of Science and Technology, Luoyang, China (Yimin Mao); Renmin Hospital of Wuhan University, Wuhan, China (Ke Hu); Jilin People's Hospital, Jilin, China (Qiang Gao); Nanjing Drum Tower Hospital, the Affiliated Hospital of Nanjing University Medical School, Nanjing, China (Hang Xu); The Affiliated Hospital of Xuzhou Medical University, Xuzhou, China (Xiao Liu); Panjin Liaoyou Gem Flower Hospital (Liaohe Oilfield General Hospital), Panjin, China (Yanmei Wu); Inner Mongolia Baogang Hospital, Hohhot, China (Jingping Yang); Weifang Second People's Hospital, Weifang, China (Yong Zhang); Shanxi Bethune Hospital, Taiyuan, China (Xiansheng Liu); The Fourth People's Hospital of Zigong, Zigong, China (Changguo He); The First People's Hospital of Yibin, Yibin, China (Yao Luo); The First People's Hospital of Kashgar Prefecture, Kashgar, China (Li Li); The First People's Hospital of Anning, Kunming, China (Bingkun Zhang); Jiaxing First Hospital, Jiaxing, China (Liangfeng Zhu); Lishui People's Hospital, Lishui, China (Ning Zhang); Zaozhuang Municipal Hospital, Zaozhuang, China (Chunmei Yu); Dalian Central Hospital, Dalian, China (Feng Xu); Yuncheng Central Hospital, Yuncheng, China (Tong Chen); Taizhou First People's Hospital, Taizhou, China (Binliang Wang); The First People's Hospital of Yinchuan, Yinchuan, China (Lijun Chen); Jiande First People's Hospital, Jiande, China (Zhonghua Fang); Zhongshan People's Hospital, Guangdong, China (Chizhuai Liu); Yongcheng Central Hospital, Henan, China (Liang Zhu); Xindu Hospital of Traditional Chinese Medicine, Chengdu, China (Huan Yan); Yan'an Hospital of Kunming City, Kunming, China (Cilin Han); and The Affiliated Hospital of North Sichuan Medical College, Sichuan, China (Li Jiang).
